# A hybrid deconvolution approach for estimation of *in vivo* non-displaceable binding for brain PET targets without a reference region

**DOI:** 10.1371/journal.pone.0176636

**Published:** 2017-05-01

**Authors:** Francesca Zanderigo, J. John Mann, R. Todd Ogden

**Affiliations:** 1 Molecular Imaging and Neuropathology Division, New York State Psychiatric Institute, New York, New York, United States of America; 2 Department of Psychiatry, Columbia University, New York, New York, United States of America; 3 Department of Radiology, Columbia University, New York, New York, United States of America; 4 Department of Biostatistics, Columbia University, Mailman School of Public Health, New York, New York, United States of America; University of Manchester, UNITED KINGDOM

## Abstract

**Background and aim:**

Estimation of a PET tracer’s non-displaceable distribution volume (V_ND_) is required for quantification of specific binding to its target of interest. V_ND_ is generally assumed to be comparable brain-wide and is determined either from a reference region devoid of the target, often not available for many tracers and targets, or by imaging each subject before and after blocking the target with another molecule that has high affinity for the target, which is cumbersome and involves additional radiation exposure. Here we propose, and validate for the tracers [^11^C]DASB and [^11^C]CUMI-101, a new data-driven hybrid deconvolution approach (HYDECA) that determines V_ND_ at the individual level without requiring either a reference region or a blocking study.

**Methods:**

HYDECA requires the tracer metabolite-corrected concentration curve in blood plasma and uses a singular value decomposition to estimate the impulse response function across several brain regions from measured time activity curves. HYDECA decomposes each region’s impulse response function into the sum of a parametric non-displaceable component, which is a function of V_ND_, assumed common across regions, and a nonparametric specific component. These two components differentially contribute to each impulse response function. Different regions show different contributions of the two components, and HYDECA examines data across regions to find a suitable common V_ND_. HYDECA implementation requires determination of two tuning parameters, and we propose two strategies for objectively selecting these parameters for a given tracer: using data from blocking studies, and realistic simulations of the tracer. Using available test-retest data, we compare HYDECA estimates of V_ND_ and binding potentials to those obtained based on V_ND_ estimated using a purported reference region.

**Results:**

For [^11^C]DASB and [^11^C]CUMI-101, we find that regardless of the strategy used to optimize the tuning parameters, HYDECA provides considerably less biased estimates of V_ND_ than those obtained, as is commonly done, using a non-ideal reference region. HYDECA test-retest reproducibility is comparable to that obtained using a V_ND_ determined from a non-ideal reference region, when considering the binding potentials BP_P_ and BP_ND_.

**Conclusions:**

HYDECA can provide subject-specific estimates of V_ND_ without requiring a blocking study for tracers and targets for which a valid reference region does not exist.

## Introduction

Positron Emission Tomography (PET) in the brain involves administration of a tracer dose of a radioactively labeled molecule (i.e., tracer) that binds to a specific target [1]. The tracer signal in the tissue combines signal from tracer “specifically” bound to the target and tracer “non-specifically” bound to other macromolecules or free in tissue water. Estimation of tracer non-displaceable uptake allows quantification of the specific binding potential between tracer and target [2, 3]. The tracer non-displaceable distribution volume (V_ND_), corresponding to “non-specifically” bound and free tracer, is commonly estimated using either the tracer binding level in a reference region that is devoid of the target [2, 3], or a blocking study which involves a baseline PET scan and a second scan with a blocking drug administered just before the tracer [4, 5].

In a valid reference region, the tracer is either free or only “non-specifically” bound, and its volume of distribution (V_T_) in such a region (V_T-RR_) is typically assumed to represent the brain-wide V_ND_. For many targets this approach is not appropriate because there is no valid reference region, as the target is present throughout the brain [6–16], and thus the signal in any region includes some specific binding. Using an invalid reference region over-estimates V_ND_, causing underestimation of binding potentials [16], and can confound interpretation of results [17–20]. Automatic extraction of a reference region signal using cluster analysis [7, 10, 12] of the brain PET data is often not successful, or greatly depends on the data used to train the clustering algorithm [21].

Alternatively, a blocking study with tracer injections before and after a saturating dose of an antagonist with high affinity for the same target of interest allows estimation of brain-wide V_ND_ using a Lassen plot [4, 5]. However, performing a blocking study in each subject is cumbersome, costly, doubles the radiation exposure, can involve side effects related to the blocking agent, and is therefore generally avoided in clinical research.

A parametric pseudo-reference tissue model was proposed [22] for tracers that have no ideal reference region, which provides estimates only for the binding potential BP_ND_ [2] and not for V_ND_ and thus not for binding potentials BP_P_ and BP_F_ [2], and assumes that BP_ND_ in the pseudo reference region can be estimated from additional competition data. A genomic plot was also recently proposed, which provides V_ND_ estimates only at the population level and requires that the brain maps of messenger RNA transcripts of the specific target of interest be available from the Allen Brain Atlas [23].

Based on compartment models (CMs) [24], we proposed to perform at the individual subject level simultaneous estimation of a common V_ND_ across regions [25] when no valid reference region is available. However, for some tracers such as [^11^C]DASB (target: serotonin transporter), the simultaneous estimation of V_ND_ across regions often fails to give a unique solution. Separately, we also showed [26] that nonparametric deconvolution is an alternative quantification approach for PET data, which computes binding potentials comparable to estimates by CMs, and for some tracers, shows superior test-retest performance than quantification by CMs [26].

We now propose a new hybrid deconvolution approach (HYDECA) that combines deconvolution and simultaneous search across regions to calculate a brain-wide V_ND_ when arterial blood data are available but a valid reference region is not. HYDECA is validated for [^11^C]DASB and [^11^C]CUMI-101 (target: serotonin 1A receptor) using simulations and blocking studies [11, 27], and evaluated in test-retest datasets [10, 28].

## Materials and methods

### Human subjects and animal studies

Data from published blocking studies in baboons [27] and humans [11], and test-retest datasets in humans [10, 28] were used. Human studies were performed in accordance with the 1964 Declaration of Helsinki and its later amendments and approved by The Institutional Review Boards of Columbia University Medical Center (CUMC) and New York State Psychiatric Institute (NYSPI). Animal studies were performed with the approval of the CUMC and NYSPI Institutional Animal Care and Use Committees, according to all applicable regulations governing the use of animals in research.

### Nonparametric quantification

According to the extended indicator dilution theory [26, 29], the tracer signal in tissue in a brain region i, C_Ti_(t), after correction for the presence of tracer in vasculature, is a scaled convolution between the metabolite-corrected input function in the arterial plasma, C_P_(t), and the so-called tissue residue function, R_i_(t):

$$C_{T_{i}}\left(t\right)=K_{i}\left(C_{P}⊗R_{i}\right)\left(t\right)$$ (1)

While K_i_ [mL⋅cm^-3^⋅min^-1^] is a proportionality constant, R_i_(t) is defined in the theory of the indicator-dilution method as the fraction of indicator that remains in the tissue after an idealized bolus input concentration at time zero. Initially, the residue must be unity (R_i_(0) = 1) and from there it decreases (or at least does not increase) with time (refer to [29] for details).

Among many nonparametric approaches that can be used to estimate the impulse response function (IRF) in each region i, *IRF*_*i*_(*t*) = *K*_*i*_*R*_*i*_(*t*), from known C_P_(t) and C_Ti_(t), we proposed using singular value decomposition (SVD) with data-driven selection of the threshold that we described elsewhere [26].

### Hybrid deconvolution approach

In the context of PET reversible radiotracers, R_i_(t) can be interpreted as the fraction of tracer molecules remaining in the tissue over time, and these molecules can be specifically bound to the target, free in water or bound to other molecules. HYDECA decomposes each region R_i_(t) into the sum of a parametric non-displaceable component, which is approximated as a mono-exponential function depending on V_ND_, assumed common across regions (see details below and comments on the validity of this approximation in the Discussion), and a nonparametric specific component. For any choice of V_ND_ defining the non-displaceable component, the nonparametric specific component can be estimated by subtraction.

**Fig 1:**
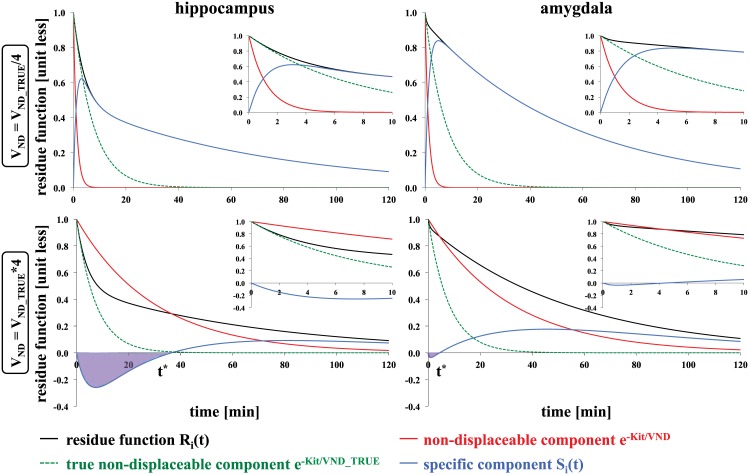
Illustration of the idea behind the algorithm in the hybrid deconvolution approach. R_i_(t) curves (black lines) calculated using the IRF of the 2TCM and values of the kinetic rates derived from a study with [^11^C]DASB for 2 representative regions. Red solid lines indicate the non-displaceable component calculated with a V_ND_ that is 1/4 the size of (top) and 4 times higher than (bottom) the true underlying V_ND_; green dotted lines indicate the non-displaceable component calculated with the true underlying V_ND_; blue lines indicate the corresponding specific component. Inset plots are added to allow closer inspection of the first 10 minutes after tracer injection. The time t* is derived from the data as the time point after which the specific component has consistently positive values. IRF: impulse response function; 2TCM: two-tissue compartment model; V_ND_: non-displaceable distribution volume.

Performing such a decomposition for observed PET data can be challenging, but the goal of HYDECA is to objectively ascertain a “reasonable” V_ND_ value by examining data across regions. To illustrate this idea, Fig 1 shows R_i_(t) curves in two representative regions, calculated using a two-tissue CM (2TCM) [24] (see Eq 8) and based on kinetic rates derived from data with [^11^C]DASB [28]. Non-displaceable component curves based on two “unreasonable” choices of V_ND_ (a value that is 1/4 and 4 times the magnitude of the true V_ND_, respectively) are compared to the non-displaceable component calculated with the true V_ND_ (the “most reasonable” choice). The non-displaceable component and the corresponding specific component differentially contribute to R_i_(t) and two effects can be observed. The first effect is that, at time zero, the difference between the slope of R_i_(t) and that of the non-displaceable component is small, if the V_ND_ value is close to the true V_ND_. The second effect is that, when the V_ND_ value used for the non-displaceable component is larger than the true V_ND_, the corresponding specific component results in negative values, violating its positivity constraints. Different regions show different contributions of non-displaceable and specific component to R_i_(t). HYDECA is based on finding a V_ND_ value that, across regions, provides the best compromise between these two effects.

To do so, HYDECA requires as input C_P_(t) and C_Ti_(t) curves from a pre-determined set of N brain regions, once corrected for the presence of vasculature. In our implementation we assumed a brain-wide blood volume of 5%. HYDECA estimates V_ND_ as follows:

IRF_i_(t) is estimated in each region i from C_Ti_(t) and C_P_(t) using SVD as described [26]; K_i_ is obtained as the value of IRF_i_(t) at time zero (R_i_(0) = 1 by definition for an idealized bolus input; see “Implementation” section below for comments); R_i_(t) is then obtained dividing IRF_i_(t) by the K_i_ estimate;

R_i_(t) is expressed in each region i as the sum of a parametric non-displaceable component (corresponding to an ideal one-tissue CM with distribution volume of V_ND_), R_ND_(t), and a nonparametric specific component, S_i_(t):

$$R_{i}\left(t\right)=R_{ND}\left(t\right)+S_{i}\left(t\right)=e^{-tK_{i}/V_{ND}}+S_{i}\left(t\right)$$ (2)

Assuming a mono-exponential for R_ND_(t) represents an approximation (if a 2TCM is needed to describe the data in a given region, R_ND_(t) would be described by two exponentials [24]), whose validity varies across regions (see comments on the validity of this approximation in the Discussion); HYDECA examines data across regions to find a suitable common V_ND_. The property expressed in Eq (2) can be derived from CMs as follows:

$$V_{T}=V_{ND}+V_{S}=V_{ND}+BP_{P}$$ (3)

$$V_{T}=\int_{0}^{+\infty }K_{i}R_{i}(\tau )d\tau =K_{i}\int_{0}^{+\infty }R_{ND}\left(\tau \right)d\tau +K_{i}\int_{0}^{+\infty }S_{i}\left(\tau \right)d\tau$$ (4)

$$R_{i}\left(t\right)=R_{ND}\left(t\right)+S_{i}\left(t\right)=e^{-tK_{i}/V_{ND}}+S_{i}\left(t\right)$$ (5)

with binding potential BP_P_ as in [2]; HYDECA expresses parametrically only R_ND_(t).

For fixed values of the tuning parameters β and γ, the following cost function is minimized over V_ND_ using all N regions:

$$\psi \left(V_{ND}\right)=\Sigma_{i=1}^{N}\left\{\Sigma_{t_{j}<\gamma }{\left[R_{i}\left(t_{j}\right)-e^{-t_{j}K_{i}/V_{ND}}\right]}^{2}+\beta \int_{0}^{t^{*}}\left[R_{i}\left(\tau \right)-e^{-\tau K_{i}/V_{ND}}\right]d\tau \right\}$$ (6)

Minimization of the first term in Eq (6), which represents the residual sum of squares between R_i_(t) and R_ND_(t), calculated up to time γ after tracer injection, identifies V_ND_ values that provide R_ND_(t) curves with a slope close to the slope of R_i_(t) at time zero. Given the difficulty in accurately estimating slopes from noisy data, the difference in the slope is approximated as the residual sum of squares between the two curves. The tuning parameter γ controls the number of data points considered for this calculation. Minimization of the second term, which represents the negative area of the curve of the corresponding S_i_(t), in the case that a portion of S_i_(t) assumes negative values, penalizes V_ND_ values that lead to unphysiological S_i_(t) values (Fig 1). If S_i_(t) is everywhere positive then there is no contribution of the second term. If S_i_(t) has negative values, then the time t* is data-derived as the time point after which S_i_(t) has consistently positive values. The tuning parameter β weights the contribution of the second term relatively to the first term. We propose and compare two strategies for setting optimal values for the tuning parameters β and γ for a given tracer.

### Tuning with simulations

One strategy involves simulating data that imitate characteristics of real data for the tracer at hand, letting β and γ vary over a grid of possible values, and identifying optimal β and γ as those values that allow HYDECA to generate an estimate of V_ND_ that, on average across all simulated instances, is closest to the true simulated V_ND_ (V_ND_TRUE_).

We considered a metabolite-corrected input function C_P_(t) and kinetic rate values in the same brain regions we considered in previous publications [25, 26] based on available data [10, 28]: cerebellar gray matter (CGM), temporal lobe (TEM), hippocampus (HIP), dorsal caudate (DCA), amygdala (AMY), and ventral striatum (VST), for [^11^C]DASB; CGM, HIP, TEM and occipital lobe (OCC), and cingulate (CIN) for [^11^C]CUMI-101. Noise-free C_Ti_(t) curves were generated for each region using a 2TCM [3, 24]:

$$C_{T_{i}}\left(t\right)=K_{1i}\left(C_{P}⊗IRF_{i}\right)\left(t\right)$$ (7)

$$IRF_{i}\left(t\right)=\frac{k_{3i}+k_{4i}-\alpha_{1i}}{\alpha_{2i}-\alpha_{1i}}e^{-t\alpha_{1i}}+\frac{\alpha_{2i}-k_{3i}-k_{4i}}{\alpha_{2i}-\alpha_{1i}}e^{-t\alpha_{2i}}$$ (8)

$$\{\begin{array}{c} \alpha_{1i}=\frac{k_{2i}+k_{3i}+k_{4i}-\sqrt{{\left(k_{2i}+k_{3i}+k_{4i}\right)}^{2}-4k_{2i}k_{4i}}}{2}\\ \alpha_{2i}=\frac{k_{2i}+k_{3i}+k_{4i}+\sqrt{{\left(k_{2i}+k_{3i}+k_{4i}\right)}^{2}-4k_{2i}k_{4i}}}{2} \end{array}$$ (9)

where K_1i_, k_2i_, k_3i_, and k_4i_ are the values for the kinetic rate parameters of region i. Table 1 lists the kinetic rate values used in each of two simulated cases per tracer: 1) common V_ND_TRUE_ is 3, and 50% of the tracer V_T-RR_ is specific binding (cerebellar grey matter V_T_ ~6); 2) common V_ND_TRUE_ is 5, and ~17% of the tracer V_T-RR_ is specific binding (cerebellar grey matter V_T_ ~6).

**Table 1:** List of kinetic rate values used in the simulations.

region	K_1_ [mL·cm^-3^·min^-1^]	k_2_ [min^-1^]	k_3_ [min^-1^]	k_4_ [min^-1^]	V_T_ [mL·cm^-3^]
**[**^**11**^**C]DASB**					
**V**_**ND**_ **= 3**					
cerebellar grey matter	0.540	0.180	0.540	0.550	5.96
temporal lobe	0.463	0.154	0.505	0.168	12.00
hippocampus	0.404	0.135	0.103	0.026	14.92
dorsal caudate	0.509	0.170	2.900	0.615	17.14
amygdala	0.380	0.127	1.510	0.270	19.78
ventral striatum	0.476	0.159	3.452	0.357	32.02
**V**_**ND**_ **= 5**					
cerebellar grey matter	0.542	0.108	0.542	2.816	5.96
temporal lobe	0.463	0.093	0.505	0.361	12.00
hippocampus	0.404	0.081	0.103	0.052	14.92
dorsal caudate	0.509	0.102	2.900	1.194	17.14
amygdala	0.380	0.076	1.510	0.511	19.78
ventral striatum	0.476	0.095	3.452	0.639	32.02
**[**^**11**^**C]CUMI-101**					
**V**_**ND**_ **= 3**					
cerebellar grey matter	0.450	0.150	0.050	0.050	6.00
hippocampus	0.360	0.120	0.130	0.030	16.00
temporal lobe	0.300	0.100	0.100	0.050	9.00
occipital lobe	0.450	0.150	0.080	0.050	7.80
cingulate	0.330	0.110	0.090	0.050	8.40
**V**_**ND**_ **= 5**					
cerebellar grey matter	0.450	0.090	0.050	0.250	6.00
hippocampus	0.360	0.072	0.130	0.059	16.00
temporal lobe	0.300	0.060	0.100	0.125	9.00
occipital lobe	0.450	0.090	0.080	0.143	7.80
cingulate	0.330	0.066	0.090	0.132	8.40

In all cases, we simulated Gaussian noise with zero mean. To ensure realistic noise characteristics, the variance-covariance matrix used to generate simulated noise was estimated from a matrix of residuals, standardized across time points, from the fits for the considered regions using available data [10, 28]. In all cases, we simulated 1000 C_Ti_(t) curves for each region.

For each tracer and V_ND_TRUE_ case, we then: 1) considered a grid of β (0.5 to 14; step: 0.5) and γ values (1 to 30 minutes after tracer injection; step: 1); 2) calculated the cost function (Eq 6) corresponding to all combinations of β and γ within the grids, and over a grid of V_ND_ values (0.1 to 7; step: 0.1), in each of the simulated instances; 3) considered the average cost function (across instances) corresponding to each of the combinations of β and γ; 4) estimated V_ND_ as the value that minimizes each of these average cost functions; and 5) calculated the corresponding absolute estimation error as |V_ND_TRUE_−V_ND_|. After obtaining the association between each combination of β and γ within the grids and the corresponding bias of the V_ND_ estimate, we selected as optimal β and γ derived via simulations (β_opt-S_, γ_opt-S_) the values providing the smallest bias.

### Tuning with blocking studies

Another strategy involves using blocking studies, if available, letting β and γ vary over a grid of possible values, and identifying optimal β and γ as those values in correspondence of which HYDECA provides a V_ND_ that is, on average across all subjects in the dataset, the closest to the V_ND_ estimated using both scans before and after blocking and Lassen plot [5] (V_ND_LASSEN_).

We examined 13 healthy controls imaged with [^11^C]DASB before and after administration of sertraline [11], and 8 pairs of scans performed on healthy baboons with [^11^C]CUMI-101 before and after either WAY100635 or 8-OH-DPAT [27].

In each pair, we computed V_ND_LASSEN_ using both scans before and after blocking and the same regions considered in simulation. We then: 1) considered the same grids for β and γ as in tuning with simulations; 2) calculated the HYDECA cost function corresponding to all combinations of β and γ within the grids, and over a grid of V_ND_ values (0.1 to 30; step: 0.1), using in each pair only the scan before blocking and the same regions considered in simulation; 3) estimated V_ND_ as the value that minimizes each of these cost functions; 4) calculated the corresponding absolute estimation error as |V_ND_LASSEN_−V_ND_|; and 5) calculated the average (across all subjects within a tracer) estimation error obtained for each combination of β and γ. After obtaining the association between each combination of β and γ within the grids and the corresponding bias in the V_ND_ estimate, we selected as optimal β and γ derived via blocking studies (β_opt-B_, γ_opt-B_) the values providing the smallest bias.

In each scan before blocking, we calculated the percent difference (PD_VND_) between V_ND_ estimated using HYDECA (with tuning parameters set with either strategy) and the corresponding V_ND_LASSEN_, as PD_VND_ = 100⋅|V_ND_LASSEN_−V_ND_|/ V_ND_LASSEN_.

### Implementation

HYDECA, implemented in Matlab R2012b (www.mathworks.com/), is a fast algorithm that runs in ~14 seconds for one subject on an iMac machine, 3.5 GHz Intel Core i7 Processor, once β and γ are determined. The most computationally demanding component is the data-driven selection of SVD threshold [26]. The computational time required to optimize the tuning parameters initially for a given tracer depends on the selected strategy. If this is done using simulations, this can take up to a few hours. Using blocking studies, the computation is complete within a few minutes.

Only with an idealized bolus input does R_i_(t) reach its maximum at time zero, and in such a case, K_i_ could be derived from the value of the reconstructed *IRF*_*i*_(t) = *K*_*i*_*R*_*i*_(*t*) at time zero. With a realistic bolus infusion of the tracer, R_i_(t) reaches its maximum at some time t > 0, and implementation that estimates K_i_ as the maximum of the reconstructed IRF_i_(t) is preferable. Furthermore, in our implementation, all deconvolved R_i_(t) curves are first shifted to have their maximum value correspond to time zero before calculating the HYDECA cost function in Eq (6). We do not perform any correction for a physiological delay between C_Ti_(t) in the different regions and C_P_(t).

### Estimation using the non-ideal reference region

To investigate the bias of HYDECA V_ND_ estimates relative to estimates measured using the Lassen plot, and in comparison to the common practice of setting V_ND_ equal to V_T-RR_ even when the reference region is known not to be valid, we utilized only the scans before blocking in the two available datasets to calculate V_T_ in CGM starting from C_P_(t) and C_Ti_(t), using both a 2TCM [24] and Likelihood Estimation in Graphical Analysis (LEGA) [30]. CGM was chosen as reference region as it has the lowest V_T_ [10, 28] and least displacement of all regions examined in our blocking studies [11, 27]. LEGA provides the best test-retest reproducibility over analysis with CMs and other graphical approaches for estimates with both tracers [10, 28]. PD_VND_ with respect to V_ND_LASSEN_ was also calculated for V_T-RR_ obtained with both 2TCM (V_T-RR,2TCM_) and LEGA (V_T-RR,LEGA_).

### Application to test-retest data

As V_ND_ is estimated in order to calculate binding potentials, we considered two available test-retest datasets with [^11^C]DASB [28] and [^11^C]CUMI-101 [10] and investigated the reproducibility of binding potentials derived using HYDECA versus using the purported reference region (CGM). Both test-retest datasets included only healthy controls, who were imaged with the radiotracer in question twice in one day (once in the morning, once in the afternoon) in a test-retest study design. In all scans, we calculated V_ND_ (using HYDECA with optimal β and γ set with either strategy, and considering the same regions used in simulation), V_T-RR,2TCM_, and V_T-RR,LEGA_. For each test-retest pair and region, we calculated the percent difference PD_VND-TRT_ as $100\frac{\left|V_{TEST}-V_{RETEST}\right|}{\left(V_{TEST}+V_{RETEST}\right)/2}$, where V_TEST_ is the V_ND_ or V_T-RR_ estimate in the test scan, and V_RETEST_ the V_ND_ or V_T-RR_ estimate in the retest scan. We compared PD_VND-TRT_ values obtained from the different methods using a two-tailed paired t-test, considering all possible pairwise combinations of methods.

In all scans and regions, we then calculated the binding potentials BP_P_ and BP_ND_ [2] based on: 1) V_ND_ by HYDECA (V_ND_ (HYDECA)), as BP_P-HYBRID_ = V_T_ (LEGA)–V_ND_ (HYDECA) and BP_ND-HYBRID_ = BP_P-HYBRID_/V_ND_ (HYDECA), where V_T_ (LEGA) is the V_T_ obtained in each target region using LEGA; 2) V_T-RR,LEGA_, as BP_P-RR,LEGA_ = V_T_ (LEGA)–V_T-RR,LEGA_ and BP_ND-RR,LEGA_ = BP_P-RR,LEGA_/V_T-RR,LEGA_; 3) V_T-RR,2TCM_, as BP_P-RR,2TCM_ = V_T_ (2TCM)–V_T-RR,2TCM_ and BP_ND-RR,2TCM_ = BP_P-RR,2TCM_/V_T-RR,2TCM_, with V_T_ (2TCM) the V_T_ obtained in each target region using 2TCM; and 4) direct calculation from 2TCM kinetic rates, as BP_P-direct,2TCM_ = K_1_k_3_/k_2_k_4_ and BP_ND-direct,2TCM_ = k_3_/k_4_.

For each test-retest pair and region, we calculated the percent difference for the binding potentials (PD_BPP_ and PD_BPND_) as $100\frac{\left|BP_{T}-BP_{RT}\right|}{\left(BP_{T}+BP_{RT}\right)/2}$, (BP_T_: test estimate; BP_RT_: re-test estimate), and computed average and standard deviation (SD) (across subjects within a tracer) of PD_BPP_ and PD_BPND_ values in each region. We compared PD_BPP_ and PD_BPND_ values obtained from the different methods using a two-tailed paired t-test, region by region, considering all possible pairwise combinations of methods.

## Results

### Simulation studies

#### Tuning parameters optimization

**Fig 2:**
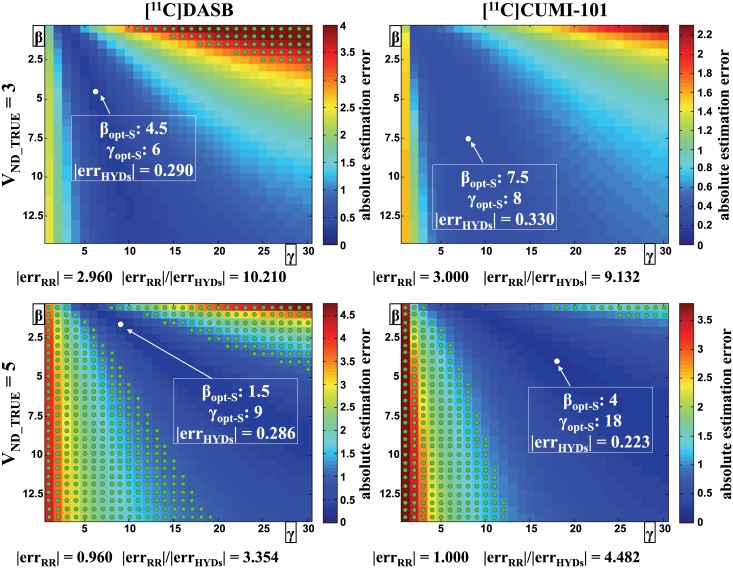
Optimization of tuning parameters β and γ using simulations. Average absolute error in the estimation of V_ND_ as a function of the values for the tuning parameters β and γ, for all simulated cases (V_ND_TRUE_ = 3, top; V_ND_TRUE_ = 5, bottom) and tracers. Each point in the matrices correspond to a specific combination of β (vertical axis) and γ (horizontal axis) values in the selected grids, and represents the average (across simulated instances) absolute distance between V_ND_ estimate obtained by HYDECA, using the corresponding combination of β and γ, and V_ND_TRUE._ The white circle indicates the optimal combination of the tuning parameters (β_opt-S_, γ_opt-S_) derived using simulations in each case, and the average absolute error in the estimation of V_ND_ in correspondence of the optimal tuning parameters is reported (|err_HYDs_|). Green circles indicate the combinations of β and γ for which HYDECA provides an average absolute error in the estimation of V_ND_ that is higher than the absolute error committed by assuming V_ND_ equal to the V_T_ in the non-ideal reference region (CGM) (|err_RR_|), and the ratio between |err_RR_| and |err_HYDs_| in correspondence of the optimal β and γ is reported. V_ND_: non-displaceable distribution volume; V_ND_TRUE_: true simulated V_ND_; V_T_: tracer total volume of distribution; CGM: cerebellum grey matter.

Optimization of β and γ using simulations and effects of β and γ values on V_ND_ estimates obtained by HYDECA are shown in Fig 2. As γ (number of points considered for the first term in Eq (6)) increases, β needs to correspondingly increase to weight more the second term in Eq (6)), in order to minimize bias in V_ND_ estimation.

For each tracer and V_ND_TRUE_ case, we selected the optimal β and γ values in correspondence of which HYDECA provides the least biased estimation of V_ND_ (Fig 2, white circles): for [^11^C]DASB, β_opt-S_ = 4.5 and γ_opt-S_ = 6 (V_ND_TRUE_ = 3), β_opt-S_ = 1.5 and γ_opt-S_ = 9 (V_ND_TRUE_ = 5); for [^11^C]CUMI-101, β_opt-S_ = 7.5 and γ_opt-S_ = 8 (V_ND_TRUE_ = 3), β_opt-S_ = 4 and γ_opt-S_ = 18 (V_ND_TRUE_ = 5).

When 50% of V_T-RR_ is specific binding (V_ND_TRUE_ = 3), for [^11^C]CUMI-101 HYDECA provides an average error in the V_ND_ estimation smaller than using V_T-RR_ with any other combination of β and γ. The error committed using a non-ideal reference region (|err_RR_|) is nine times greater than the error committed using HYDECA (|err_HYDs_|) (|err_RR_|/|err_HYDs_| = 9.132). With [^11^C]DASB, HYDECA provides an average estimation error smaller than using V_T-RR_ in correspondence of most combinations of β and γ, with exception of a subset of values (green circles in Fig 2) (|err_RR_|/|err_HYDs_| = 10.210).

When only 17% of the V_T_ in the non-ideal reference region is specific binding (V_ND_TRUE_ = 5), for both tracers the number of combinations of β and γ in correspondence of which HYDECA provides an average estimation error smaller than using V_T-RR_ is reduced. However, HYDECA with optimized β and γ still generated a robustly more accurate estimate of V_ND_ than V_T-RR_ ([^11^C]CUMI-101: |err_RR_|/|err_HYDs_| = 4.482; [^11^C]DASB: |err_RR_|/|err_HYDs_| = 3.354).

#### Cost functions and estimation bias with optimized tuning parameters

**Fig 3:**
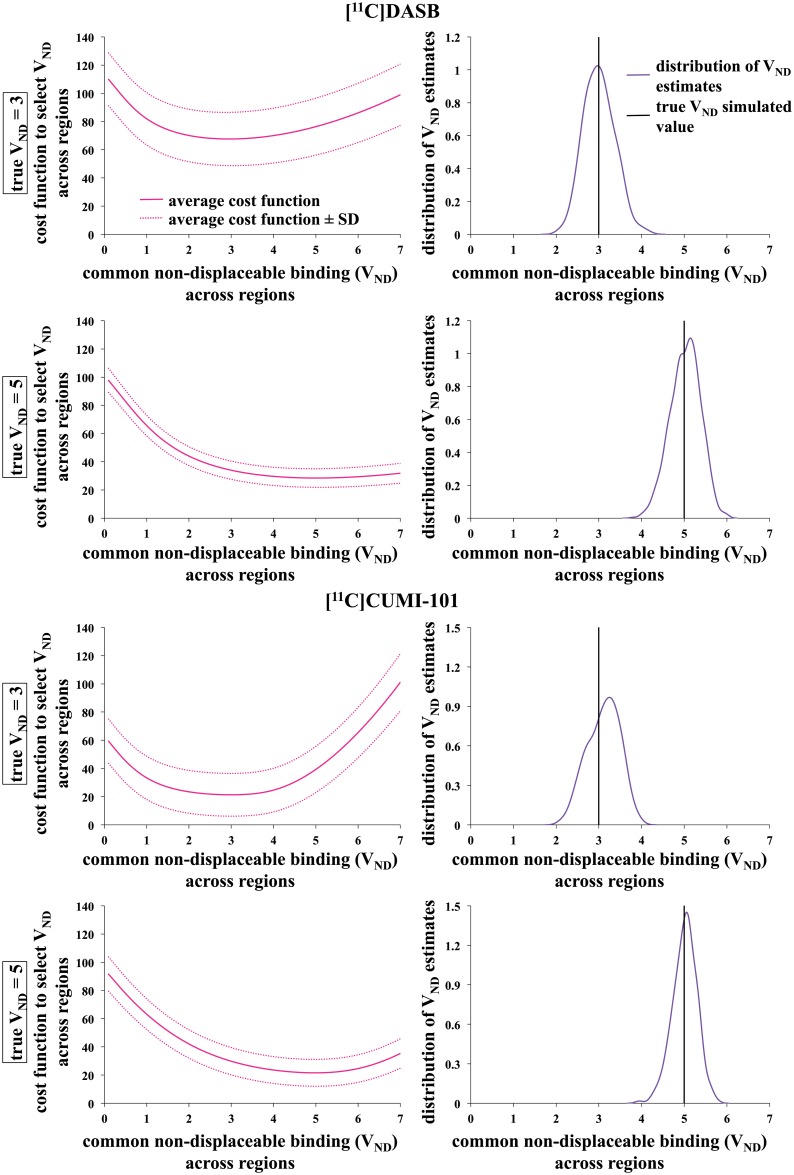
Cost functions and estimation bias with tuning parameters β and γ optimized using simulations. HYDECA cost functions (average across 1000 instances, plus and minus one standard deviation) and corresponding distribution of V_ND_ estimates obtained in simulations with V_ND_TRUE_ = 3 and V_ND_TRUE_ = 5 with [^11^C]DASB (top) and [^11^C]CUMI-101 (bottom), using the optimal tuning parameters (β_opt-S_, γ_opt-S_) derived using simulations in each case. V_ND_: non-displaceable distribution volume; V_ND_TRUE_: true simulated V_ND_.

HYDECA cost function curves (Eq 6) using (β_opt-S_, γ_opt-S_) as determined using simulations are convex and unimodal (Fig 3). The corresponding distributions of V_ND_ estimates show a bias, calculated as the average of (V_ND_TRUE_−V_ND_) across instances, of -0.008 (V_ND_TRUE_ = 3) and -0.024 (V_ND_TRUE_ = 5) ([^11^C]DASB), and -0.108 (V_ND_TRUE_ = 3) and -0.010 (V_ND_TRUE_ = 5) ([^11^C]CUMI-101). The variance of the estimates is 0.133 (V_ND_TRUE_ = 3) and 0.126 (V_ND_TRUE_ = 5) ([^11^C]DASB), and 0.144 (V_ND_TRUE_ = 3) and 0.082 (V_ND_TRUE_ = 5) ([^11^C]CUMI-101).

### Blocking studies

#### Tuning parameters optimization

**Fig 4:**
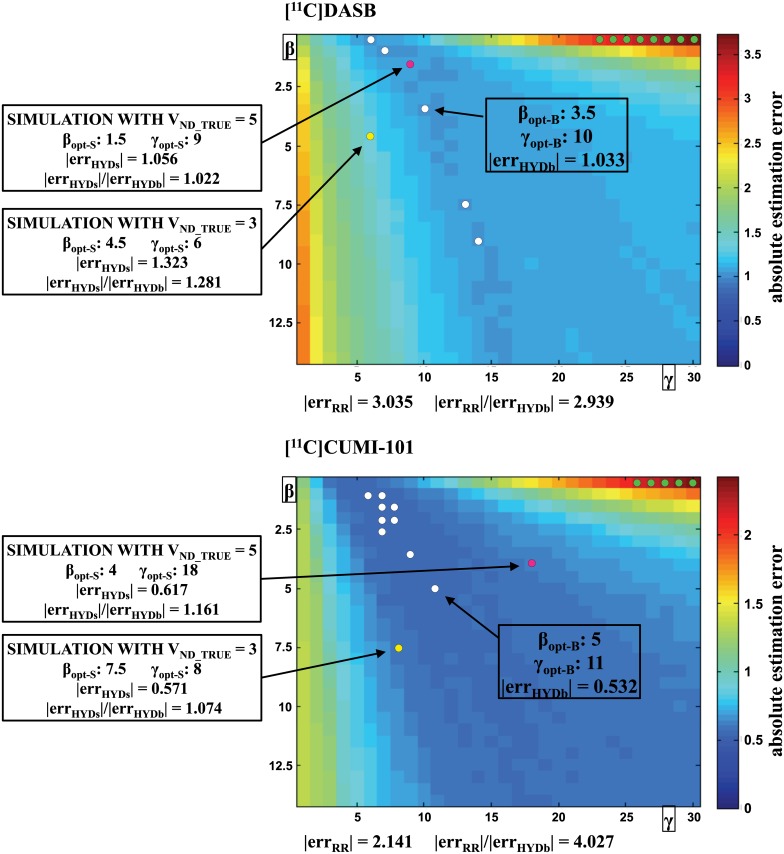
Optimization of tuning parameters β and γ using blocking studies. Average absolute error in the estimation of V_ND_ as a function of the values for the tuning parameters β and γ for both tracers. Each point in the matrices correspond to a combination of β and γ values in the selected grids, and represents the average (across scans within the same tracer) absolute distance between the V_ND_ estimated by HYDECA, using the corresponding combination of β and γ, and V_ND_LASSEN_. White circles indicate the optimal combinations of the tuning parameters (β_opt-B_, γ_opt-B_) derived using the blocking studies, and the average absolute error in the estimation of V_ND_ in correspondence of (β_opt-B_, γ_opt-B_) is reported (|err_HYDb_|). Green circles indicate the combinations of β and γ for which HYDECA provides an average absolute error in the estimation of V_ND_ that is higher than the absolute error committed by using the V_T_ in the CGM as an estimation of V_ND_ (|err_RR_|), and the ratio between |err_RR_| and |err_HYDb_| in correspondence of the optimal β and γ is reported. The yellow and pink circle indicates the optimal combination of the tuning parameters (β_opt-S_, γ_opt-S_) derived using simulation with V_ND_TRUE_ = 3 and V_ND_TRUE_ = 5, respectively, and the ratio between |err_HYDs_| (in correspondence of β_opt-S_ and γ_opt-S_) and |err_HYDb_| (in correspondence of β_opt-B_ and γ_opt-B_) is reported. V_ND_: non-displaceable distribution volume; V_ND_LASSEN_: V_ND_ estimated using both scans before and after blocking and Lassen plot; V_T_: tracer total volume of distribution; CGM: cerebellum grey matter.

Optimization of β and γ using blocking studies, and effect of β and γ values on HYDECA estimates of V_ND_, are shown in Fig 4. With our data, we found five combinations of β and γ with [^11^C]DASB and nine with [^11^C]CUMI-101 that provide the same minimum absolute error in the V_ND_ estimation (Fig 4, white circles); any of these combinations could be regarded as the optimal β and γ. We selected: β_opt-B_ = 3.5, γ_opt-B_ = 10 for [^11^C]DASB; β_opt-B_ = 5, γ_opt-B_ = 11 for [^11^C]CUMI-101.

With both tracers, only very few combinations of β and γ (Fig 4, green circles) provide an average absolute error in the V_ND_ estimation (|err_HYDb_|) that is larger than the error using V_T-RR_ (|err_RR_|). The |err_RR_|/|err_HYDb_| ratio is 2.939 for [^11^C]DASB, and 4.027 for [^11^C]CUMI-101.

For [^11^C]DASB, HYDECA with (β_opt-S_, γ_opt-S_) derived using simulation with V_ND_TRUE_ = 5 (Fig 4, pink circles) provides the closest results to HYDECA with (β_opt-B_, γ_opt-B_) derived using blocking studies (|err_HYDs_|/|err_HYDb_| = 1.022). A V_ND_TRUE_ value of 5 is closer to average V_ND_ values obtained using Lassen plot in the available [^11^C]DASB blocking studies. For [^11^C]CUMI-101, HYDECA with (β_opt-S_, γ_opt-S_) derived using simulation with V_ND_TRUE_ = 3 (Fig 4, yellow circles) provides the closest results to HYDECA with (β_opt-B_, γ_opt-B_) derived using blocking studies (|err_HYDs_|/|err_HYDb_| = 1.074). A V_ND_TRUE_ value of 3 is closer to average V_ND_ values obtained using Lassen plot and the available [^11^C]CUMI-101 blocking studies. In the results that follow, we refer to (β_opt-S_, γ_opt-S_) for [^11^C]DASB and [^11^C]CUMI-101 as those obtained with the simulation studies with V_ND_TRUE_ = 5 and V_ND_TRUE_ = 3, respectively.

#### Estimation bias with optimized tuning parameters

**Fig 5:**
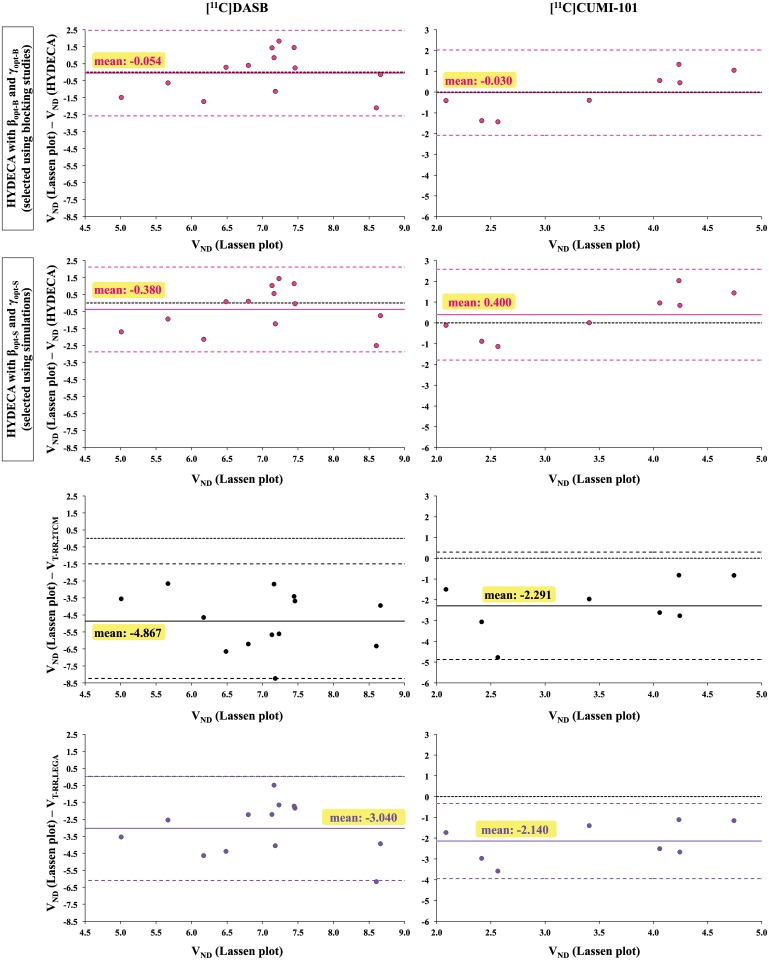
Estimation bias with tuning parameters β and γ optimized using blocking studies. Difference between V_ND_LASSEN_ and V_ND_ estimates obtained by HYDECA with (β_opt-B_, γ_opt-B_) set using blocking studies (y-axis; first row), between V_ND_LASSEN_ and V_ND_ estimates obtained by HYDECA with (β_opt-S_, γ_opt-S_) set using simulations (y-axis; second row), between V_ND_LASSEN_ and V_ND_ estimated as the V_T_ in the CGM using 2TCM (y-axis; third row), and between V_ND_LASSEN_ and V_ND_ estimated as the V_T_ in the CGM using LEGA (y-axis; bottom), as a function of V_ND_LASSEN_ (x-axis) in individual scans in the blocking study with [^11^C]DASB (left) and [^11^C]CUMI-101 (right). Solid lines indicate the average error; dotted lines indicate average error ± 1.96 standard deviation. The zero line is the dotted black line. V_ND_: non-displaceable distribution volume; V_ND_LASSEN_: V_ND_ estimated using both scans before and after blocking and Lassen plot; V_ND_ (HYDECA): V_ND_ estimated using HYDECA; V_T-RR,2TCM_: distribution volume in the non-ideal reference region calculated using 2TCM; V_T-RR,LEGA_: distribution volume in the non-ideal reference region calculated using LEGA; V_T_: tracer total volume of distribution; CGM: cerebellum grey matter; 2TCM: two-tissue compartment model; LEGA: Likelihood Estimation in Graphical Analysis.

Application of HYDECA to individual scans in the blocking studies, with β and γ optimized using either strategies, in comparison to the use of V_T-RR_ is shown in Fig 5. V_ND_ estimates by HYDECA with either sets of tuning parameters are considerably less biased, relative to V_ND_ estimates from the Lassen plot, than those using V_T-RR_. Estimation of V_ND_ using 2TCM in the non-ideal reference region is more biased than that obtained by HYDECA and LEGA for both considered tracers. Average (± SD) PD_VND_ values across subjects are: 15.48% (± 9.82) using HYDECA with (β_opt-B_, γ_opt-B_), 15.40% (± 11.65) using HYDECA with (β_opt-S_, γ_opt-S_), 44.16% (± 22.52) using V_T-RR,LEGA_, and 70.04% (± 24.00) using V_T-RR,2TCM_ ([^11^C]DASB); 27.81% (± 19.03) using HYDECA with (β_opt-B_, γ_opt-B_), 26.08% (± 17.24) using HYDECA with (β_opt-S_, γ_opt-S_), 70.26% (± 42.82) using V_T-RR,LEGA_, and 76.10% (± 56.03) using V_T-RR,2TCM_ ([^11^C]CUMI-101). All V_ND_ and V_T-RR_ estimates for all approaches and both blocking datasets are reported in Table 2. For both tracers, average (across subjects within each tracer) V_ND_ estimates by HYDECA, with β and γ optimized using either strategies, are closer than both LEGA and 2TCM to average values calculated using Lassen plot, which is considered standard in the field for *in vivo* estimation of V_ND_, and SD values are overall lower than those for LEGA and 2TCM.

**Table 2:** V_ND_ and V_T-RR_ estimates for all approaches in the available blocking datasets and their summary statistics.

V_ND_ ESTIMATES in BLOCKING DATASETS					
	HYDECA (β_opt-S_, γ_opt-S_) (baseline scan)	HYDECA (β_opt-B_, γ_opt-B_) (baseline scan)	V_T-RR,2TCM_ (baseline scan)	V_T-RR,LEGA_ (baseline scan)	LASSEN (baseline and block scan)
**[**^**11**^**C]DASB**					
sub 1	6.4	6.2	13.1	10.9	6.5
sub 2	6.6	6.3	8.3	8.2	5.7
sub 3	7.5	7.2	11.1	9.3	7.5
sub 4	6.7	6.4	13.0	9.0	6.8
sub 5	11.1	10.7	14.9	14.8	8.6
sub 6	8.4	8.3	15.4	11.2	7.2
sub 7	6.6	6.3	9.8	7.7	7.2
sub 8	9.4	8.8	12.6	12.6	8.7
sub 9	5.8	5.4	12.8	8.9	7.2
sub 10	6.3	6	10.9	9.2	7.4
sub 11	6.1	5.7	12.8	9.4	7.1
sub 12	8.3	7.9	10.8	10.8	6.2
sub 13	6.7	6.5	8.6	8.6	5.0
**mean**	**7.4**	**7.1**	**11.9**	**10.0**	**7.0**
**SD**	**1.5**	**1.5**	**2.2**	**2.0**	**1.0**
**[**^**11**^**C]CUMI-101**					
scan 1	3.1	3.5	6.7	6.6	4.1
scan 2	2.2	2.5	3.6	3.8	2.1
scan 3	3.4	3.8	7.0	6.9	4.2
scan 4	2.2	2.9	5.1	5.3	4.2
scan 5	3.3	3.7	5.6	5.9	4.7
scan 6	3.4	3.8	5.4	4.8	3.4
scan 7	3.7	4	7.3	6.1	2.6
scan 8	3.3	3.8	5.5	5.4	2.4
**mean**	**3.1**	**3.5**	**5.8**	**5.6**	**3.5**
**SD**	**0.6**	**0.5**	**1.2**	**1.0**	**1.0**

### Test-retest studies

Average (across subjects within each tracer) estimates of V_ND_ and V_T-RR_ in the test-retest datasets (Table 3) are consistent with corresponding values found in the blocking datasets (Table 2), although in the case of [^11^C]CUMI-101 the two datasets are in different species. V_ND_ values we obtain with HYDECA average 27% of total binding in ventral striatum for [^11^C]DASB, and 22% of the total binding in hippocampus for [^11^C]CUMI-101, which is generally in line with reports for other PET tracers [31, 32].

**Table 3:** V_ND_ and V_T-RR_ estimates for all approaches in the available test-retest datasets and their summary statistics.

V_ND_ ESTIMATES in TEST-RETEST DATASETS								
	HYDECA (β_opt-S_, γ_opt-S_)		HYDECA (β_opt-B_, γ_opt-B_)		V_T-RR,2TCM_		V_T-RR,LEGA_	
	TEST	RETEST	TEST	RETEST	TEST	RETEST	TEST	RETEST
**[**^**11**^**C]DASB**								
sub 1	10.1	10.6	9.5	10.3	14.9	15.2	14.9	15.2
sub 2	7.2	8.2	7	7.8	10.8	10.9	10.8	10.9
sub 3	6.5	6.9	6.1	6.7	8.2	8.3	8.2	8.3
sub 4	9.8	8	9.5	7.6	12.1	11.9	12.1	11.7
sub 5	5.6	5.1	5.2	4.7	9.0	9.1	9.0	9.1
sub 6	7.7	8.4	7.3	8.1	11.1	9.5	9.3	9.5
sub 7	11.6	10.2	11.3	10	14.8	14.4	14.8	13.2
sub 8	8.6	8.3	8.4	8	15.4	11.6	11.2	11.6
sub 9	10.1	10.8	9.8	10.1	12.6	12.8	12.6	12.8
sub 10	6.9	7.8	6.8	7.4	13.6	9.7	9.2	9.7
**mean**	**8.4**	**8.4**	**8.1**	**8.1**	**12.3**	**11.3**	**11.2**	**11.2**
**SD**	**1.9**	**1.7**	**1.9**	**1.7**	**2.5**	**2.3**	**2.4**	**2.1**
**[**^**11**^**C]CUMI-101**								
sub 1	2.5	2.7	3.1	3.7	4.8	5.1	5.0	5.2
sub 2	2.6	2.3	2.9	2.7	4.8	4.8	4.9	4.8
sub 3	2.4	2.4	3.1	2.9	4.9	4.6	5.1	4.8
sub 4	2.5	3.1	3.4	3.6	5.6	5.5	5.7	5.8
sub 5	3.1	2.6	3.6	3.7	6.1	7.2	6.1	6.7
sub 6	1.6	2.4	2.6	3.5	5.5	5.9	5.8	6.1
**mean**	**2.5**	**2.6**	**3.1**	**3.4**	**5.3**	**5.5**	**5.4**	**5.5**
**SD**	**0.5**	**0.3**	**0.4**	**0.4**	**0.5**	**1.0**	**0.5**	**0.8**

Test-retest PD_VND-TRT_ values for V_ND_ (Table 4) from the different methods are not statistically significantly different from each other, with the exception of [^11^C]DASB, in which case PD_VND-TRT_ values obtained by HYDECA are statistically significantly higher (indicating worse reproducibility) than those derived by LEGA (p-values: 0.003 with β and γ set via simulation; 0.002 with β and γ set via blocking study). See Discussion for factors affecting the reproducibility of V_ND_ by HYDECA.

**Table 4:** Test-retest PD_VND_ percent difference values for HYDECA V_ND_, V_T-RR,LEGA_ and V_T-RR,2TCM_.

V_ND_ TEST-RETEST PERCENT DIFFERENCE				
	HYDECA (β_opt-S_, γ_opt-S_)	HYDECA (β_opt-B_, γ_opt-B_)	2TCM	LEGA
**[**^**11**^**C]DASB**				
pair #1	4.83%	8.08%	1.65%	1.68%
pair #2	12.99%	10.81%	0.47%	0.47%
pair #3	5.97%	9.38%	0.58%	0.58%
pair #4	20.22%	22.22%	1.30%	3.01%
pair #5	9.35%	10.10%	1.54%	1.10%
pair #6	8.70%	10.39%	15.39%	2.47%
pair #7	12.84%	12.21%	2.82%	11.49%
pair #8	3.55%	4.88%	28.55%	2.82%
pair #9	6.70%	3.02%	1.89%	1.72%
pair #10	12.24%	8.45%	33.27%	5.78%
**mean**	**9.74%**	**9.95%**	**8.75%**	**3.11%**
**SD**	**4.98%**	**5.13%**	**12.53%**	**3.32%**
**[**^**11**^**C]CUMI-101**				
pair #1	7.69%	17.65%	5.07%	3.91%
pair #2	12.24%	7.14%	0.37%	2.27%
pair #3	0.00%	6.67%	7.05%	5.99%
pair #4	21.43%	5.71%	2.90%	1.58%
pair #5	17.54%	2.74%	16.86%	9.42%
pair #6	40.00%	29.51%	6.79%	3.62%
**mean**	**16.48%**	**11.57%**	**6.51%**	**4.46%**
**SD**	**13.75%**	**10.15%**	**5.66%**	**2.86%**

**Fig 6:**
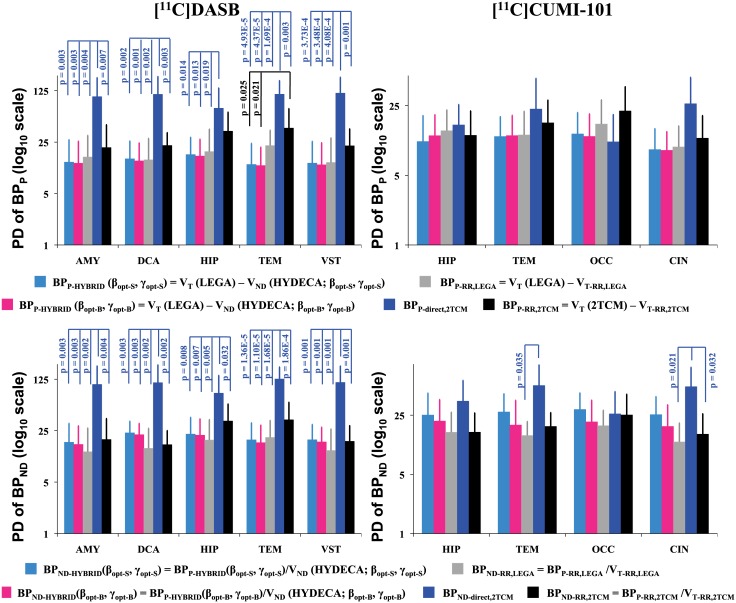
Reproducibility of binding potentials estimated using HYDECA, LEGA, and 2TCM. Average plus standard deviation (across test-retest pairs of scans within each tracer) test-retest percent difference PD_BPP_ values calculated in each of the considered region for [^11^C]DASB (left) and [^11^C]CUMI-101 (right), using BP_P_ based on V_ND_ from HYDECA, BP_P_ based on V_T-RR,LEGA_, BP_P_ calculated directly from the 2TCM kinetic rates, and BP_P_ based on V_T-RR,2TCM_ (top). Corresponding values for the test-retest percent difference PD_BPND_ (bottom). Vertical axes are reported in logarithmic scale to allow for easier visualization of the direct 2TCM results based on 2TCM kinetic rates. Statistically significant comparisons (p-value ≤ 0.05) are indicated. AMY: amygdala; CIN: cingulate; DCA: dorsal caudate; HIP: hippocampus; OCC: occipital lobe; TEM: temporal lobe; VST: ventral striatum; PD_BPP_: percent difference for BP_P_; PD_BPND_: percent difference for BP_ND_; V_ND_: non-displaceable distribution volume; V_T-RR,LEGA_: tracer total distribution volume in the non-ideal reference region estimated using LEGA; LEGA: Likelihood Estimation in Graphical Analysis; 2TCM: two-tissue compartment model; V_T-RR,2TCM_: racer total distribution volume in the non-ideal reference region estimated using 2TCM.

Reproducibility of the binding potentials estimated using HYDECA V_ND_, with β and γ optimized using either strategies, is compared to that of binding potentials based on V_T-RR,LEGA_, V_T-RR,2TCM_, or direct estimation by 2TCM in Fig 6. PD_BPP_ values obtained using HYDECA with either sets of optimized tuning parameters are close to each other and comparable to values obtained using V_T-RR,LEGA_. PD_BPP_ values from the different methods are not statistically significantly different from each other, with the exception of 2TCM direct estimation, where PD_BPP_ values are statistically significantly higher (indicating worse reproducibility) than those of all other methods in the case of [^11^C]DASB in all brain regions except HIP (range of p-values: 4.37E-5 to 0.019), and of 2TCM indirect estimation in the case of [^11^C]DASB in TEM, for which PD_BPP_ values are statistically significantly higher than those derived by HYDECA (p-values: 0.025 with β and γ set via simulation; 0.021 with β and γ set via blocking study).

Similarly, PD_BPND_ values from the different methods are not statistically significantly different from each other, with the exception of 2TCM direct estimation in the case of [^11^C]DASB in all regions (PD_BPND_ values statistically significantly higher than those of all other methods; range of p-values: 1.10E-5 to 0.032), and 2TCM direct estimation in the case of [^11^C]CUMI-101 in TEM and CIN, for which PD_BPND_ values are statistically significantly higher than those derived by LEGA (p-values: 0.035 in TEM; 0.021 in CIN) and by 2TCM indirect estimation (p-value: 0.032 in CIN).

Overall, the test-retest reproducibility of binding potentials obtained using all methods reported in Fig 6 are comparable, with the exception of 2TCM direct estimation in the case of [^11^C]DASB.

## Discussion

HYDECA is a data-driven approach that estimates V_ND_ for each individual based on his/her PET data from multiple brain regions. HYDECA is intended for tracers and targets for which a valid reference region does not exist. If a valid reference region does in fact exist, then binding potentials based on V_T-RR_ or on reference region approaches are likely to be more accurate than those based on HYDECA.

### Tuning parameters

HYDECA implementation requires determination of two tuning parameters, herein denoted β and γ, and we propose two possible strategies to make this choice for a given tracer: using data from blocking studies, or realistic simulations of the tracer in question. It should be noted that using the same tuning parameters across subjects imaged with the same tracer does not result in estimating the same V_ND_ in each subject.

Of the two strategies, the one using blocking studies involves less subjective judgment. When blocking study data are not available, simulations can be used, but simulated V_ND_ values, kinetic rates, and measurement errors should be chosen carefully to obtain realistic representation of the data with the tracer in question. For established tracers, simulations can be set up using kinetic rate values derived from available data or from the literature. For a new tracer, both simulations and validation with blocking studies are recommended.

For [^11^C]DASB and [^11^C]CUMI-101, our results suggest that, regardless of the strategy used to optimize the tuning parameters, HYDECA estimates of V_ND_ are considerably less biased than those obtained based on V_T-RR_. Even with a “sub-optimal” choice of the tuning parameters, HYDECA estimates of V_ND_ are generally less biased than using a non-ideal reference region (Figs 2 and 4). Although the selection strategies can provide different values for β and γ, the resulting bias in the estimation of V_ND_ is similar (|errHYDs|/|errHYDb| ratios in Fig 4).

If we were to optimize β and γ individually for each subject in the blocking datasets, we would observe quite large inter-subject variability in the optimal β and γ: β = 8.85 ± 4.64, γ = 19.77 ± 11.80 ([^11^C]DASB); β = 9.31 ± 5.04, γ = 18.38 ± 12.37 ([^11^C]CUMI-101). For subjects in the dataset that is used for tuning parameter selection, using individually optimized β and γ instead of values optimized on average across subjects, as we suggest, would lead to an even less biased V_ND_ estimation. However, the question of which β and γ values to use when applying HYDECA to a subject imaged with the same tracer, but for which a blocking scan is not available, would remain. Individually optimized β and γ values are not obtainable in standard practice.

### Utility

HYDECA is a workable algorithm that can be applied to estimate individual V_ND_ in absence of a reference region or individual blocking data, and could therefore be extremely useful in both clinical and research settings. If the target selected for a given PET application lacks a valid reference region, there is no way to accurately estimate V_ND_ (and consequently specific binding to the target), unless one performs a blocking scan for each subject. HYDECA can provide an alternative convenient quantification approach. For tracers for which HYDECA tuning parameters have already been determined, the published optimized tuning parameters can be used. Otherwise, published blocking studies for the tracer in question would constitute the basis to either tune HYDECA directly (if data are accessible) or to set up a simulation.

### Reproducibility

HYDECA estimates of V_ND_ (with either strategy to set tuning parameters) lead to binding potentials estimates with test-retest reproducibility that are comparable to estimates based on V_T-RR_. Note that average PD_BPP_ values are overall lower when based on HYDECA compared with values based on V_T-RR_, and not merely because V_ND_ estimates by HYDECA are consistently lower than corresponding V_T-RR_. Detailed related information is provided in the Supplementary Materials (S3 and S4 Figs, S2 Text). We observe on average worse reproducibility of the estimates based on HYDECA when considering BP_ND_ compared to BP_P_ estimates. Because of the nature of the outcome measure and performance metric used here, BP_ND_ values and their corresponding test-retest performance are more sensitive than BP_P_ to values and changes (in between test and retest scan) in the V_ND_, which appears at the denominator in the indirect definition of BP_ND_. When using the V_T_ from an invalid reference region to estimate V_ND_, reproducibility of that measure depends on, among other factors, how much the tissue time activity curve from that region changes between the test and the retest scans. HYDECA, instead, uses tissue time activity curves from multiple regions to determine V_ND_, and therefore its test-retest performance is affected, among other factors, by how much the tissue time activity curves from all of these regions change between the test and the retest scans. The test-retest percent difference values for HYDECA V_ND_ (Table 4) are on average worse than those for V_T-RR_ calculated using 2TCM and LEGA, especially in the case of [^11^C]CUMI-101. Reproducibility performance should be considered when deciding which approach to use in longitudinal studies, while the bias of the approach is more important in group comparisons and cross-sectional studies.

### Alternative strategies

**Fig 7:**
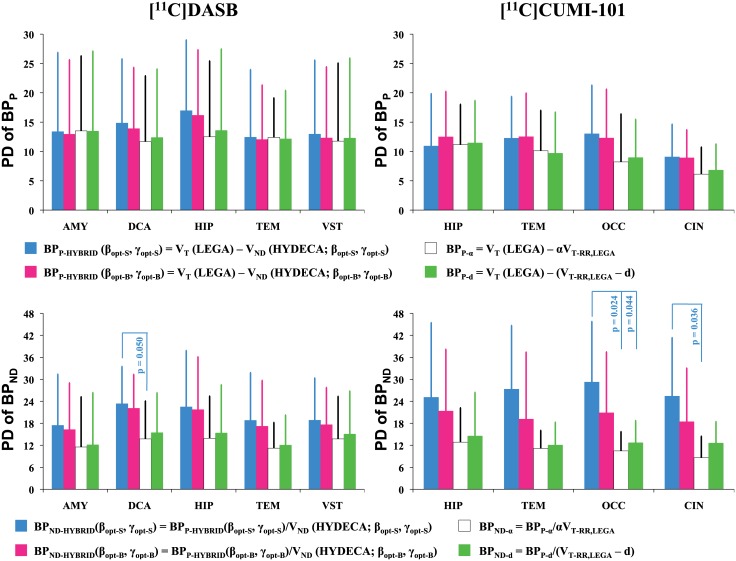
Reproducibility of binding potentials estimated using HYDECA and alternative strategies based on blocking studies. Average plus standard deviation (across test-retest pairs of scans within each tracer) test-retest percent difference PD_BPP_ values calculated in each of the considered region for [^11^C]DASB (left) and [^11^C]CUMI-101 (right), using BP_P_ based on V_ND_ from HYDECA, BP_P_ based on scaled V_T-RR,LEGA_, and BP_P_ based on average distance d (top). Corresponding values for test-retest percent difference PD_BPND_ (bottom). Statistically significant comparisons (p-value ≤ 0.05) are indicated. AMY: amygdala; CIN: cingulate; DCA: dorsal caudate; HIP: hippocampus; OCC: occipital lobe; TEM: temporal lobe; VST: ventral striatum; PD_BPP_: percent difference for BP_P_; PD_BPND_: percent difference for BP_ND_; V_ND_: non-displaceable distribution volume; V_T-RR,LEGA_: tracer total distribution volume in the non-ideal reference region estimated using LEGA; LEGA: Likelihood Estimation in Graphical Analysis.

If blocking scans are available for a certain tracer, they could be used to estimate a population-based α = V_ND_LASSEN_/V_T-RR_ ratio, which could then be used for studies with the same tracer to scale each subject V_T-RR_ in the non-ideal reference region to estimate V_ND_. We applied such approach to the two available blocking datasets. We found the following V_ND_LASSEN_/V_T-RR,LEGA_ average (± SD) α ratios: 0.710 (± 0.114) for [^11^C]DASB, and 0.619 (± 0.145) for [^11^C]CUMI-101. We applied such ratios to the subjects in the available test-retest datasets to calculate a scaled V_T-RR,LEGA_, and then calculated the corresponding BP_P-α_ = V_T_(LEGA)—αV_T-RR,LEGA_ and BP_ND-α_ = BP_P-α_/αV_T-RR,LEGA_ values and their test-retest percent difference (Fig 7). Test-retest percent differences values obtained using the different methods reported in Fig 7 are not statistically significantly different from each other in the case of BP_P_, nor in the case of BP_ND_, with the exception of [^11^C]DASB BP_ND_ in DCA, and [^11^C]CUMI-101 BP_ND_ in OCC and CIN, for which percent differences values obtained using a population-based α ratio are statistically significantly lower (indicating better reproducibility) than those based on HYDECA with β and γ set via simulation (p-value: 0.050, 0.024, and 0.036, respectively). Also see comments on BP_ND_ reproducibility in “Reproducibility” section above.

From a compartment modeling point of view, however, if there is specific binding in the non-ideal reference region, this would correspond to an additional compartment, which would require a subtraction (rather than a multiplicative adjustment) from the total V_T_ in the region, in order to be properly accounted for. A population-based distance d = V_T-RR_−V_ND_LASSEN_ can be derived if blocking scans are available for a certain tracer as in the case of the scaled V_T-RR_. In the two available blocking studies, we found the following average V_T-RR,LEGA_−V_ND_LASSEN_ distance (± SD) d: 3.04 (± 1.56) for [^11^C]DASB, and 2.14 (± 0.92) for [^11^C]CUMI-101. We applied such average distance values to the subjects in the available test-retest datasets to calculate BP_P-d_ = V_T_(LEGA)–(V_T-RR,LEGA_−d) and BP_ND-d_ = BP_P-d_/(V_T-RR,LEGA_−d), and their test-retest percent difference (Fig 7). Test-retest percent differences values of binding potentials obtained using a population-based distance d are not statistically significantly different from those of the other methods, with the exception of [^11^C]CUMI-101 BP_ND_ in OCC, where they are statistically significantly lower than those obtained based on HYDECA with β and γ set via simulation (p-value: 0.044). The reproducibility performance of HYDECA with tuning parameters set via blocking study is comparable to that of all the other methods for both binding potentials.

A fixed population-based ratio or distance approach, unlike HYDECA, would not take advantage of the information relative to V_ND_ that is implicitly contained in each individual’s PET tissue data across brain regions. Such an approach would rely on blocking studies more heavily than HYDECA, for which tuning parameter selection can alternatively be achieved using simulations.

In the Supplementary Materials (S1 and S2 Figs, S1 Text) we report results obtained on alternative nonparametric binding potentials [26] that can be calculated based on HYDECA, including their test-retest reproducibility and the comparison to 2TCM, LEGA, and alternative strategies.

### Choice of regions

Regions that are simultaneously considered should be carefully chosen in all approaches that either take advantage of simultaneous estimation across regions [25, 33–37], or jointly estimate common parameters of interest across regions, like occupancy and V_ND_ in the Lassen plot. For simultaneous estimation approaches to perform well, the regions that are considered should in general have kinetic behavior as distinct as possible [36]. Including regions with similar kinetic behavior would serve only to increase the dimensionality of the objective function without adding much useful information [36]. The variety in kinetic behavior depends greatly on the tracer at hand. In our previous experience with simultaneous-type estimation with [^11^C]DASB [33] and [^11^C]CUMI-101 [25], we had carefully selected regions to represent a broad range of kinetic behavior, while avoiding regions that tend to be noisy. We had also previously assessed the properties of nonparametric quantification in these regions using both simulated and clinical data [26]. We are therefore using the same regions in this study.

### Choice of deconvolution approach

We used here SVD for its speed and ease of implementation, and have characterized its performance in terms of reproducibility and sensitivity to noise in an earlier publication [26]. SVD can however be sensitive to potential delay and dispersion of the injected bolus [38, 39]. More robust approaches to nonparametric deconvolution [39, 40] or functional principal components analysis [41] may further improve HYDECA performance. Here we provide a framework for HYDECA and comparison between different implementations of the algorithm is beyond our scope.

### Limitations

#### Vascular correction

The tracer signal in the brain tissue can be modeled as in Eq (1) only after correction for intravascular activity. Here, following a practice common in the field, we assumed a brain-wide fractional blood volume (V_B_) of 5%. It is recommended that the V_B_ value be optimized before applying HYDECA (or any other PET quantification approach) if pathological changes in the fractional blood volume are suspected in the population at hand. HYDECA performance, as that of any PET quantification approach, may in fact be affected by an erroneous choice of the V_B_ value used to correct the tissue time activity curves. We ran an additional simulation to investigate the sensitivity of HYDECA estimates of V_ND_ to a potentially erroneous vascular correction of the measured time activity curves (details are reported in the Supplementary Materials, S5–S7 Figs, S3 Text). HYDECA estimates of V_ND_ appear to be robust to erroneous correction of the time activity curves for errors in V_B_ in the range -4% to +5% for [^11^C]DASB, and -4% to +7% for [^11^C]CUMI-101.

As blood volume may vary in the brain, using a brain-wide value may not significantly impact outcome measures such as V_T_ and binding potentials, but may impact the upslope of the tissue signal, and thus the R_i_(t) estimated nonparametrically. If V_B_ varies across regions, a case that is not trivial for any of the quantification approaches used in PET, one potential strategy to account for this within HYDECA could be incorporating the vascular correction component into the impulse response function that is nonparametrically deconvolved in each region. The problem may be treatable from a mathematical point of view, but would require careful comparison of more sophisticated approaches to deconvolution than SVD. Another potential strategy could be exploiting the semiparametric nature of HYDECA and adding V_B_ as a free-parameter to be estimated in each of the regions that are simultaneously considered, but this would require a more complex optimization procedure than the simple grid approach that we proposed for V_ND_. Correction for intravascular activity represents just as much of a problem for other approaches proposed as alternatives to compartment models [42, 43].

#### Assumption of a mono-exponential R_ND_(t) curve

To ensure identifiability of the two components of the residue function curve R(t) (non-displaceable and specific), HYDECA needs to assume a certain shape to describe the non-displaceable component, R_ND_(t). We chose, in part for its simplicity, a mono-exponential function, which would represent the impulse response function in the case of an “ideal” reference region with total distribution volume equal to V_ND_. Assuming a mono-exponential curve for R_ND_(t) represents an approximation: if a 2TCM is needed to describe the data in a given region, the R_ND_(t) curve of the region would be more appropriately described by a two-exponential function (24). We note that a similar assumption is central in the development of the very widely used simplified reference tissue model (SRTM) [44], which assumes that the total (non-displaceable plus specific) impulse response function of the target region (which, as well, would be a two-exponential function) can be reasonably approximated by a mono-exponential curve. The Supplementary Materials (S8 and S9 Figs, S4 Text) report data to evaluate the validity of such approximation for the two tracers considered here. Our evaluation indicates that a mono-exponential approximation for R_ND_(t) would be problematic only in the situation in which k_3_ >> k_4_, which means that more tracer molecules transit in a given amount of time from the non-displaceable binding state into the specific binding state than vice versa. We recommend that the simplifying assumption of a mono-exponential R_ND_(t) curve be evaluated for tracers for which it is suspected that k_3_ >> k_4_. However, we remind the reader: 1) that HYDECA uses data across many regions, for some of which the mono-exponential assumption may hold better than for others, and provides a brain-wide value of V_ND_ that satisfies certain constraints (via the HYDECA cost function) on average across such regions; and 2) that parts of the R_ND_(t) curve that are potentially erroneously determined in a region due to the simplifying mono-exponential assumption are likely to be captured by the corresponding nonparametric R_S_(t) curve, for which there is no assumption besides being positive and monotonic. We want also to stress that the assumption of a common, brain-wide V_ND_ implies that the ratio of the transfer constants (V_ND_ = K_1_/k_2_) is the same everywhere in the brain for non-specific binding. This same assumption is routinely made when using CMs and/or graphical approaches in a reference region to estimate a brain-wide common V_ND_, when constraining the K_1_ and k_2_ parameters in a 2TCM to those of a reference region, or when using SRTM.

#### Applicability to other populations

The two assumptions required to apply HYDECA are that: a) the non-displaceable distribution volume V_ND_ is uniform brain-wide within each subject (which is the same assumption regularly considered in the field when estimating V_ND_ from a reference region, or when using SRTM); and b) the non-displaceable component of the residue function, R_ND_(t), is reasonably described by a mono-exponential function (a similar assumption is considered for both reference and target region when using SRTM). So unless there is a population or group of subjects where it is suspected that these two assumptions are seriously violated, HYDECA can be applied. The presence of altered kinetics in the tissue time activity curves of such a population would be problematic for any of the other PET quantification approaches that are based on the assumptions above.

### Future investigations

Future investigations include developing a method to provide a measure of precision [45] for HYDECA V_ND_ estimates, validating HYDECA across tracers, and assessing whether performing the tuning of β and γ only once for a given tracer will suffice, which should be the case if the noise characteristics and kinetics range of independent data acquired with a tracer for which the HYDECA tuning parameters have been determined will resemble those of the data used in such determination.

## Conclusions

We showed, using two PET radiotracers that, in the absence of a valid reference region, HYDECA can provide individual estimates of a brain-wide V_ND_ without requiring a blocking study, and these estimates are less biased, with respect to estimation with Lassen plot, which is the method of reference, as it represents a standard in the field for *in vivo* estimation of V_ND_ in humans, than those obtained relying on the V_T_ in a non-ideal reference region.

## Supporting information

S1 Fig Reproducibility of alternative nonparametric binding potentials: BP_P_. Average (across test-retest pairs of scans within each tracer) test-retest percent difference PD_BPP_ values calculated in each of the considered region for [^11^C]DASB (left) and [^11^C]CUMI-101 (right), using the two nonparametric definitions of BP_P_: BP_P-END_ (top), and BP_P-NP2_ (bottom) (see definitions in S1 Text). In each plot, grey bars refer to BP_P-RR,LEGA_ = V_T_ (LEGA)–V_T-RR,LEGA_; black bars refer to BP_P-RR,2TCM_ = V_T_ (2TCM)–V_T-RR,2TCM_; white bars refer to BP_P-α_ = V_T_(LEGA)—αV_T_-_RR,LEGA_; and green bars refer to BP_P-d_ = V_T_(LEGA)–(V_T-RR,LEGA_−d). Error bars indicate standard deviation (across test-retest pairs of scans within each tracer). Vertical axes are reported in logarithmic scale to allow for easier visualization of the 2TCM results. AMY: amygdala; CIN: cingulate; DCA: dorsal caudate; HIP: hippocampus; OCC: occipital lobe; TEM: temporal lobe; VST: ventral striatum; PD_BPP_: percent difference for BP_P_. V_T_ (LEGA): tracer total distribution volume (V_T_) estimated using Likelihood Estimation in Graphical Analysis (LEGA); V_T-RR,LEGA_: V_T_ in the purported reference region estimated using LEGA; V_T_ (2TCM): V_T_ estimated using a two-tissue compartment model (2TCM); V_T-RR,2TCM_: V_T_ in the purported reference region estimated using 2TCM; α: population-based ratio of non-displaceable distribution volume (V_ND_) (from available blocking studies) over V_T_ in the purported reference region; d: population-based distance of V_T_ in the purported reference region and corresponding V_ND_ (from available blocking studies).
(PDF)

S2 Fig Reproducibility of alternative nonparametric binding potentials: BP_ND_. Average (across test-retest pairs of scans within each tracer) test-retest percent difference PD_BPND_ values calculated in each of the considered region for [^11^C]DASB (left) and [^11^C]CUMI-101 (right), using the two nonparametric definitions of BP_ND_: BP_ND-END_ (top), and BP_ND-NP2_ (bottom) (see definitions in S1 Text). In each plot, grey bars refer to BP_ND-RR,LEGA_; black bars refer to BP_ND-RR,2TCM_; white bars refer to BP_ND-α_; and green bars refer to BP_ND-d_. Error bars indicate standard deviation (across test-retest pairs of scans within each tracer). Vertical axes are reported in logarithmic scale to allow for easier visualization of the 2TCM results. AMY: amygdala; CIN: cingulate; DCA: dorsal caudate; HIP: hippocampus; OCC: occipital lobe; TEM: temporal lobe; VST: ventral striatum; PD_BPP_: percent difference for BP_P_. BP_ND-RR,LEGA_ = BP_P-RR,LEGA_/V_T-RR,LEGA_ = [V_T_ (LEGA)–V_T-RR,LEGA_]/V_T-RR,LEGA_; BP_ND-RR,2TCM_ = BP_P-RR,2TCM_/V_T-RR,2TCM_ = [V_T_ (2TCM)–V_T-RR,2TCM_]/V_T-RR,2TCM_; BP_ND-α_ = BP_P-α_/αV_T-RR,LEGA_ = [V_T_(LEGA)—αV_T_-_RR,LEGA_]/αV_T-RR,LEGA_; BP_ND-d_ = BP_P-d_/(V_T-RR,LEGA_−d) = [V_T_(LEGA)–(V_T-RR,LEGA_−d)]/(V_T-RR,LEGA_−d). V_T_ (LEGA): tracer total distribution volume (V_T_) estimated using Likelihood Estimation in Graphical Analysis (LEGA); V_T-RR,LEGA_: V_T_ in the purported reference region estimated using LEGA; V_T_ (2TCM): V_T_ estimated using a two-tissue compartment model (2TCM); V_T-RR,2TCM_: V_T_ in the purported reference region estimated using 2TCM; α: population-based ratio of non-displaceable distribution volume (V_ND_) (from available blocking studies) over V_T_ in the purported reference region; d: population-based distance of V_T_ in the purported reference region and corresponding V_ND_ (from available blocking studies).
(PDF)

S3 Fig Comparison of binding potentials and test-retest percent difference values: [^11^C]DASB test-retest dataset. Left: Scatter plots of BP_P-HYBRID_, BP_P-END_, and BP_P-NP2_ (see definitions in S1 and S2 Texts) values versus BP_P-RR,LEGA_ values obtained using the non-ideal reference region and Likelihood Estimation in Graphical Analysis (LEGA). The black solid line is the identity line. Right: Distance between test-retest percent difference (PD) values obtained using BP_P-HYBRID_ and PD values obtained using BP_P-RR,LEGA_ (y axis) versus the corresponding distance between BP_P-HYBRID_ (average of test and re-test) and BP_P-RR,LEGA_ (average of test and re-test). Open circles represent values obtained using the non-displaceable distribution volume (V_ND_) from HYDECA with β_opt-S_ and γ_opt-S_; solid circles represent values obtained using the V_ND_ from HYDECA with β_opt-B_ and γ_opt-B_. BP_P-RR,LEGA_ = V_T_ (LEGA)–V_T-RR,LEGA_; V_T_ (LEGA): tracer total distribution volume (V_T_) estimated using LEGA; V_T-RR,LEGA_: V_T_ in the purported reference region estimated using LEGA.
(PDF)

S4 Fig Comparison of binding potentials and test-retest percent difference values: [^11^C]CUMI-101 test-retest dataset. Left: Scatter plots of BP_P-HYBRID_, BP_P-END_, and BP_P-NP2_ (see definitions in S1 and S2 Texts) values versus BP_P_-_RR,LEGA_ values obtained using the non-ideal reference region and Likelihood Estimation in Graphical Analysis (LEGA). The black solid line is the identity line. Right: Distance between test-retest percent difference (PD) values obtained using BP_P-HYBRID_ and PD values obtained using BP_P-RR,LEGA_ (y axis) versus the corresponding distance between BP_P-HYBRID_ (average of test and re-test) and BP_P-RR,LEGA_ (average of test and re-test). Open circles represent values obtained using the non-displaceable distribution volume (V_ND_) from HYDECA with β_opt-S_ and γ_opt-S_; solid circles represent values obtained using the V_ND_ from HYDECA with β_opt-B_ and γ_opt-B_. BP_P-RR,LEGA_ = V_T_ (LEGA)–V_T-RR,LEGA_; V_T_ (LEGA): tracer total distribution volume (V_T_) estimated using LEGA; V_T-RR,LEGA_: V_T_ in the purported reference region estimated using LEGA.
(PDF)

S5 Fig Sensitivity of HYDECA estimates of the non-displaceable distribution volume (V_ND_) to erroneous vascular correction. Percent difference (PD_errVC_) between the non-displaceable distribution volume (V_ND_) value estimated at each instance of erroneously corrected time activity curves and the V_ND_ value estimated in correspondence of the accurately corrected set of time activity curves (y-axis), as a function of the difference between the true fractional blood volume (V_B_) value and the value adopted for correction (x-axis); dots and error bars indicate average and standard deviation across subjects, respectively, within each tracer. The dotted horizontal lines indicate the +10%, 0%, and -10% mark, respectively.
(PDF)

S6 Fig Residue function curves R(t) and vascular correction: [^11^C]CUMI-101. Residue function curves R(t) in correspondence of different errors and no error in the fractional blood volume value (V_B_), and the corresponding HYDECA cost functions, in a representative subject for [^11^C]CUMI-101. CIN: cingulate; HIP: hippocampus; OCC: occipital lobe; TEM: temporal lobe; CGM: cerebellum grey matter.
(PDF)

S7 Fig Residue function curves R(t) and vascular correction: [^11^C]DASB. Residue function curves R(t) in correspondence of different errors and no error in the fractional blood volume value (V_B_), and the corresponding HYDECA cost functions, in a representative subject for [^11^C]DASB. AMY: amygdala; DCA: dorsal caudate; HIP: hippocampus; TEM: temporal lobe; VST: ventral striatum; CGM: cerebellum grey matter.
(PDF)

S8 Fig Validity of mono-exponential assumption for the residue function non-displaceable component R_ND_(t): [^11^C]DASB. Average (across time points) square distance between the residue function non-displaceable component, R_ND_(t) (see S4 Text), with k_3_ and k_4_ >0, and R_ND_(t) with k_3_ = k_4_ = 0 as k_3_ and k_4_ vary, in 4 cases of (K_1_, k_2_) for [^11^C]DASB. VST: ventral striatum; CGM: cerebellum grey matter. K_1_, k_2_ k_3_ and k_4_: kinetic rate parameters of a two-tissue compartment model.
(PDF)

S9 Fig Validity of mono-exponential assumption for the residue function non-displaceable component R_ND_(t): [^11^C]CUMI-101. Average (across time points) square distance between the residue function non-displaceable component, R_ND_(t) (see S4 Text), with k_3_ and k_4_ >0, and R_ND_(t) with k_3_ = k_4_ = 0 as k_3_ and k_4_ vary, in 4 cases of (K_1_, k_2_) for [^11^C]CUMI-101. HIP: hippocampus; CGM: cerebellum grey matter. K_1_, k_2_ k_3_ and k_4_: kinetic rate parameters of a two-tissue compartment model.
(PDF)

S1 Text Alternative nonparametric binding potentials and their test-retest reproducibility. Supporting information and equations accompanying S1 and S2 Figs.
(PDF)

S2 Text Comparison of binding potentials and test-retest percent difference values. Supporting information and equations accompanying S3 and S4 Figs.
(PDF)

S3 Text Sensitivity to vascular correction. Supporting information and equations accompanying S5–S7 Figs.
(PDF)

S4 Text Assumption of a mono-exponential non-displaceable residue function. Supporting information and equations accompanying S8 and S9 Figs.
(PDF)
