# Supplementary material for: A hybrid deconvolution approach for estimation of in vivo non-displaceable binding for brain PET targets without a reference region
Source: PLoS One. 2017 May 1;12(5):e0176636. doi: 10.1371/journal.pone.0176636 (PMC5411064; doi:10.1371/journal.pone.0176636)
Supplement: S1 Fig — Average (across test-retest pairs of scans within each tracer) test-retest percent difference PDBPP values calculated in each of the considered region for [11C]DASB (left) and [11C]CUMI-101 (right), using the two nonparametric definitions of BPP: BPP-END (top), and BPP-NP2 (bottom) (see definitions in S1 Text). In each plot, grey bars refer to BPP-RR,LEGA = VT (LEGA)–VT-RR,LEGA; black bars refer to BPP-RR,2TCM = VT (2TCM)–VT-RR,2TCM; white bars refer to BPP-α = VT(LEGA)—αVT-RR,LEGA; and green bars refer to BPP-d = VT(LEGA)–(VT-RR,LEGA−d). Error bars indicate standard deviation (across test-retest pairs of scans within each tracer). Vertical axes are reported in logarithmic scale to allow for easier visualization of the 2TCM results. AMY: amygdala; CIN: cingulate; DCA: dorsal caudate; HIP: hippocampus; OCC: occipital lobe; TEM: temporal lobe; VST: ventral striatum; PDBPP: percent difference for BPP. VT (LEGA): tracer total distribution volume (VT) estimated using Likelihood Estimation in Graphical Analysis (LEGA); VT-RR,LEGA: VT in the purported reference region estimated using LEGA; VT (2TCM): VT estimated using a two-tissue compartment model (2TCM); VT-RR,2TCM: VT in the purported reference region estimated using 2TCM; α: population-based ratio of non-displaceable distribution volume (VND) (from available blocking studies) over VT in the purported reference region; d: population-based distance of VT in the purported reference region and corresponding VND (from available blocking studies). (PDF) [file pone.0176636.s001.pdf]

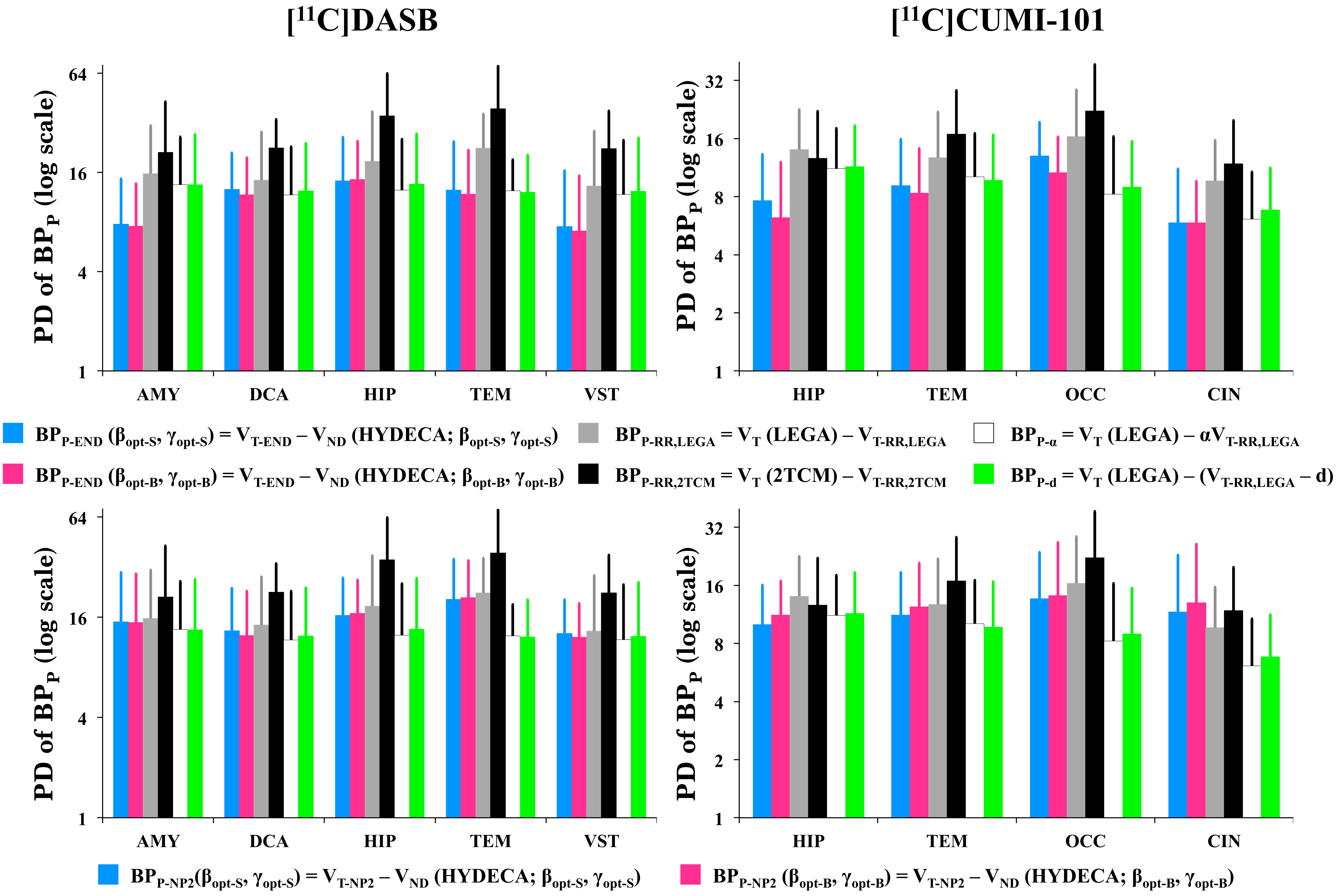
[11C]DASB
[11C]CUMI-101
PD of BP_P (log scale)
64
16
4
1
32
8
2
AMY
DCA
HIP
TEM
VST
OCC
CIN
BP_P-END (β_opt-S, γ_opt-S) = V_T-END – V_ND (HYDECA; β_opt-S, γ_opt-S)
BP_P-END (β_opt-B, γ_opt-B) = V_T-END – V_ND (HYDECA; β_opt-B, γ_opt-B)
BP_P-RR,LEGA = V_T (LEGA) – V_T-RR,LEGA
BP_P-RR,2TCM = V_T (2TCM) – V_T-RR,2TCM
BP_P-α = V_T (LEGA) – αV_T-RR,LEGA
BP_P-d = V_T (LEGA) – (V_T-RR,LEGA – d)
BP_P-NP2(β_opt-S, γ_opt-S) = V_T-NP2 – V_ND (HYDECA; β_opt-S, γ_opt-S)
BP_P-NP2 (β_opt-B, γ_opt-B) = V_T-NP2 – V_ND (HYDECA; β_opt-B, γ_opt-B)
