# Supplementary material for: A hybrid deconvolution approach for estimation of in vivo non-displaceable binding for brain PET targets without a reference region
Source: PLoS One. 2017 May 1;12(5):e0176636. doi: 10.1371/journal.pone.0176636 (PMC5411064; doi:10.1371/journal.pone.0176636)
Supplement: S2 Fig — Average (across test-retest pairs of scans within each tracer) test-retest percent difference PDBPND values calculated in each of the considered region for [11C]DASB (left) and [11C]CUMI-101 (right), using the two nonparametric definitions of BPND: BPND-END (top), and BPND-NP2 (bottom) (see definitions in S1 Text). In each plot, grey bars refer to BPND-RR,LEGA; black bars refer to BPND-RR,2TCM; white bars refer to BPND-α; and green bars refer to BPND-d. Error bars indicate standard deviation (across test-retest pairs of scans within each tracer). Vertical axes are reported in logarithmic scale to allow for easier visualization of the 2TCM results. AMY: amygdala; CIN: cingulate; DCA: dorsal caudate; HIP: hippocampus; OCC: occipital lobe; TEM: temporal lobe; VST: ventral striatum; PDBPP: percent difference for BPP. BPND-RR,LEGA = BPP-RR,LEGA/VT-RR,LEGA = [VT (LEGA)–VT-RR,LEGA]/VT-RR,LEGA; BPND-RR,2TCM = BPP-RR,2TCM/VT-RR,2TCM = [VT (2TCM)–VT-RR,2TCM]/VT-RR,2TCM; BPND-α = BPP-α/αVT-RR,LEGA = [VT(LEGA)—αVT-RR,LEGA]/αVT-RR,LEGA; BPND-d = BPP-d/(VT-RR,LEGA−d) = [VT(LEGA)–(VT-RR,LEGA−d)]/(VT-RR,LEGA−d). VT (LEGA): tracer total distribution volume (VT) estimated using Likelihood Estimation in Graphical Analysis (LEGA); VT-RR,LEGA: VT in the purported reference region estimated using LEGA; VT (2TCM): VT estimated using a two-tissue compartment model (2TCM); VT-RR,2TCM: VT in the purported reference region estimated using 2TCM; α: population-based ratio of non-displaceable distribution volume (VND) (from available blocking studies) over VT in the purported reference region; d: population-based distance of VT in the purported reference region and corresponding VND (from available blocking studies). (PDF) [file pone.0176636.s002.pdf]

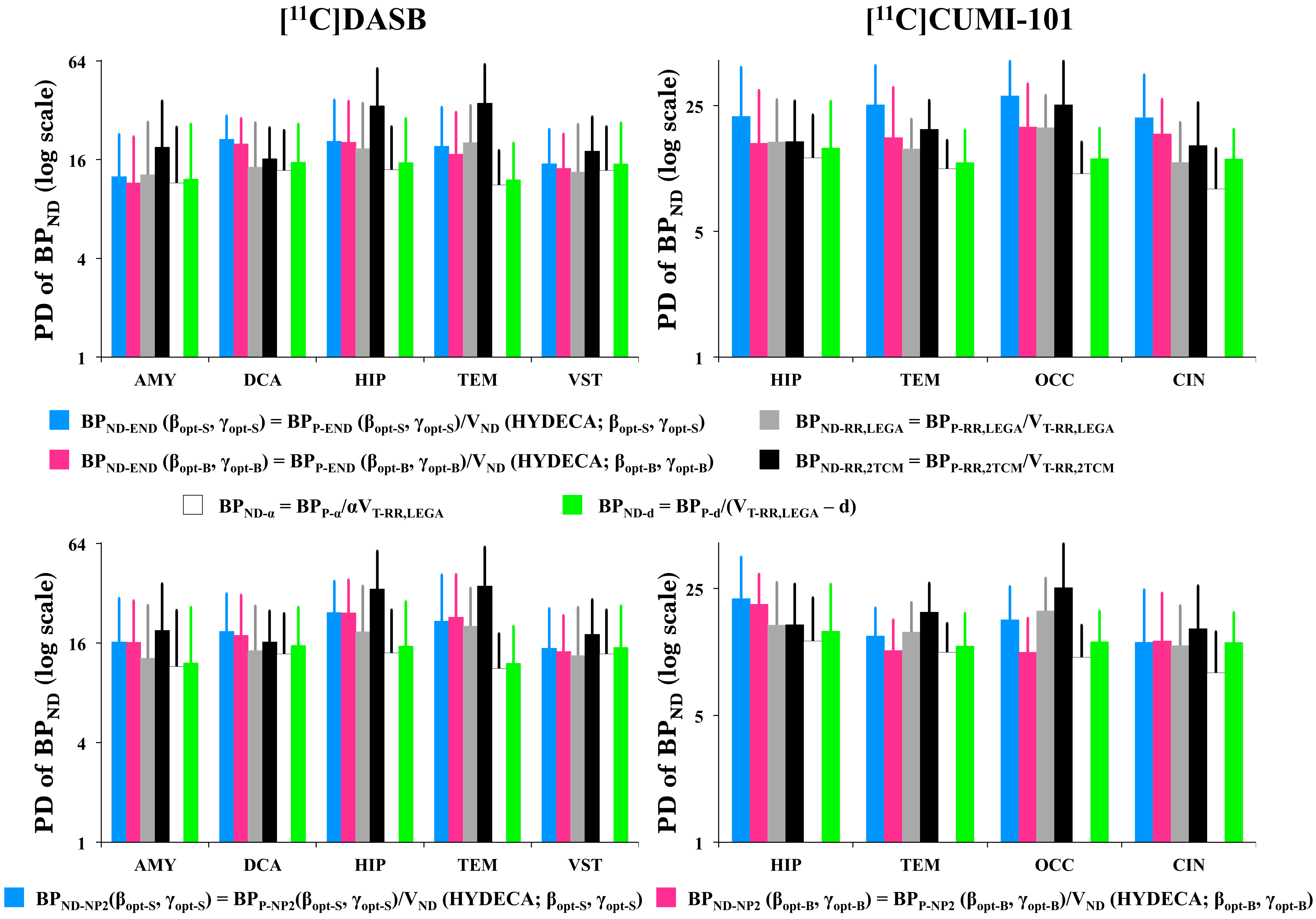

[11C]DASB
[11C]CUMI-101
PD of BP_{ND} (log scale)
64
16
4
1
25
5
1
AMY
DCA
HIP
TEM
VST
HIP
TEM
OCC
CIN
$BP_{ND\text{-}END}$ ($\beta_{opt\text{-}S}$, $\gamma_{opt\text{-}S}$) = $BP_{P\text{-}END}$ ($\beta_{opt\text{-}S}$, $\gamma_{opt\text{-}S}$)/V_{ND} (HYDECA; $\beta_{opt\text{-}S}$, $\gamma_{opt\text{-}S}$)
$BP_{ND\text{-}END}$ ($\beta_{opt\text{-}B}$, $\gamma_{opt\text{-}B}$) = $BP_{P\text{-}END}$ ($\beta_{opt\text{-}B}$, $\gamma_{opt\text{-}B}$)/V_{ND} (HYDECA; $\beta_{opt\text{-}B}$, $\gamma_{opt\text{-}B}$)
$BP_{ND\text{-}RR,LEGA}$ = $BP_{P\text{-}RR,LEGA}$/$V_{T\text{-}RR,LEGA}$
$BP_{ND\text{-}RR,2TCM}$ = $BP_{P\text{-}RR,2TCM}$/$V_{T\text{-}RR,2TCM}$
$BP_{ND\text{-}\alpha}$ = $BP_{P\text{-}\alpha}$/$\alpha V_{T\text{-}RR,LEGA}$
$BP_{ND\text{-}d}$ = $BP_{P\text{-}d}$/($V_{T\text{-}RR,LEGA}$ – d)
64
16
4
1
25
5
1
AMY
DCA
HIP
TEM
VST
HIP
TEM
OCC
CIN
$BP_{ND\text{-}NP2}$($\beta_{opt\text{-}S}$, $\gamma_{opt\text{-}S}$) = $BP_{P\text{-}NP2}$($\beta_{opt\text{-}S}$, $\gamma_{opt\text{-}S}$)/V_{ND} (HYDECA; $\beta_{opt\text{-}S}$, $\gamma_{opt\text{-}S}$)
$BP_{ND\text{-}NP2}$ ($\beta_{opt\text{-}B}$, $\gamma_{opt\text{-}B}$) = $BP_{P\text{-}NP2}$ ($\beta_{opt\text{-}B}$, $\gamma_{opt\text{-}B}$)/V_{ND} (HYDECA; $\beta_{opt\text{-}B}$, $\gamma_{opt\text{-}B}$)
