# Supplementary figures and images for: A hybrid deconvolution approach for estimation of in vivo non-displaceable binding for brain PET targets without a reference region
Source: PLoS One. 2017 May 1;12(5):e0176636. doi: 10.1371/journal.pone.0176636 (PMC5411064; doi:10.1371/journal.pone.0176636)

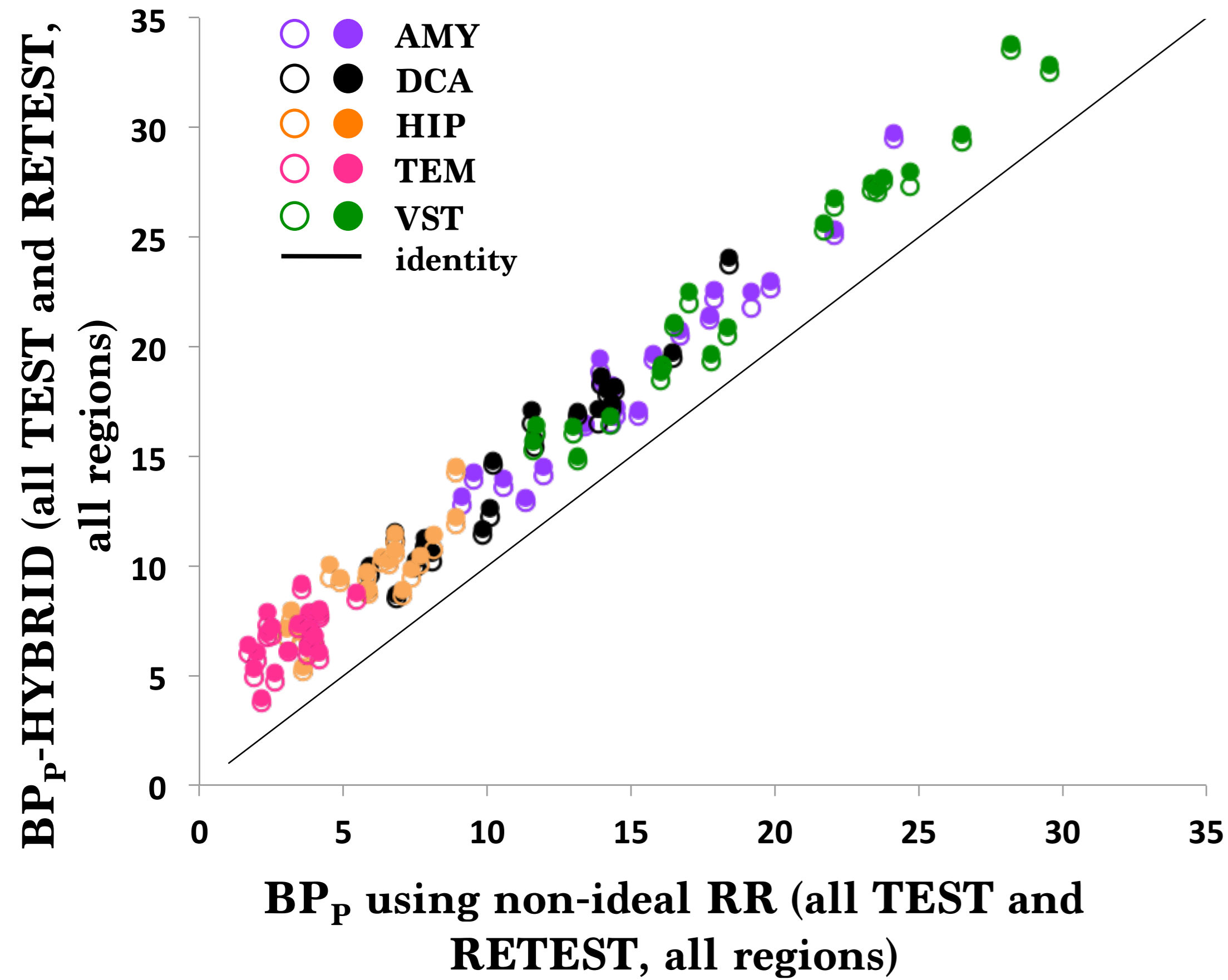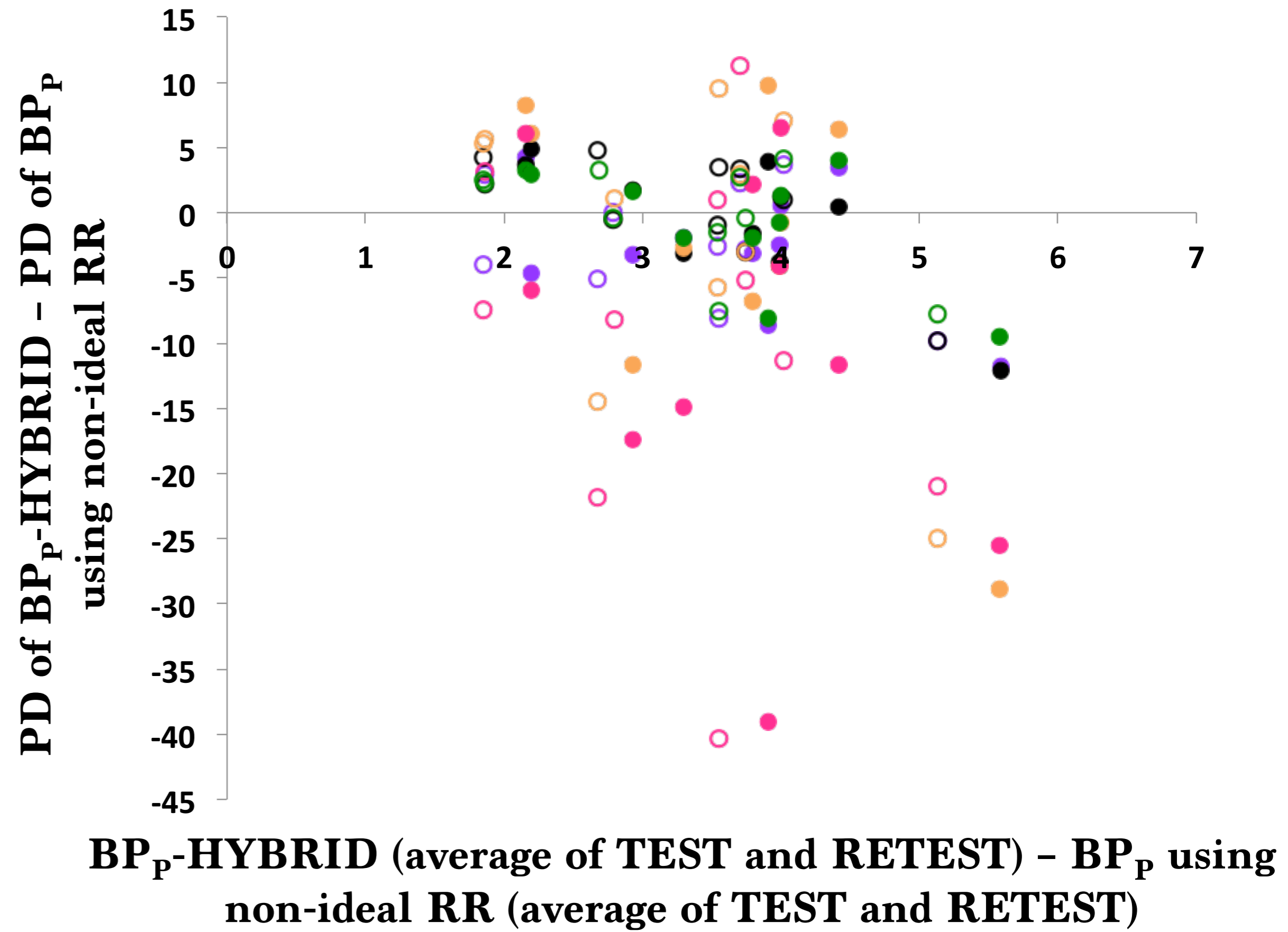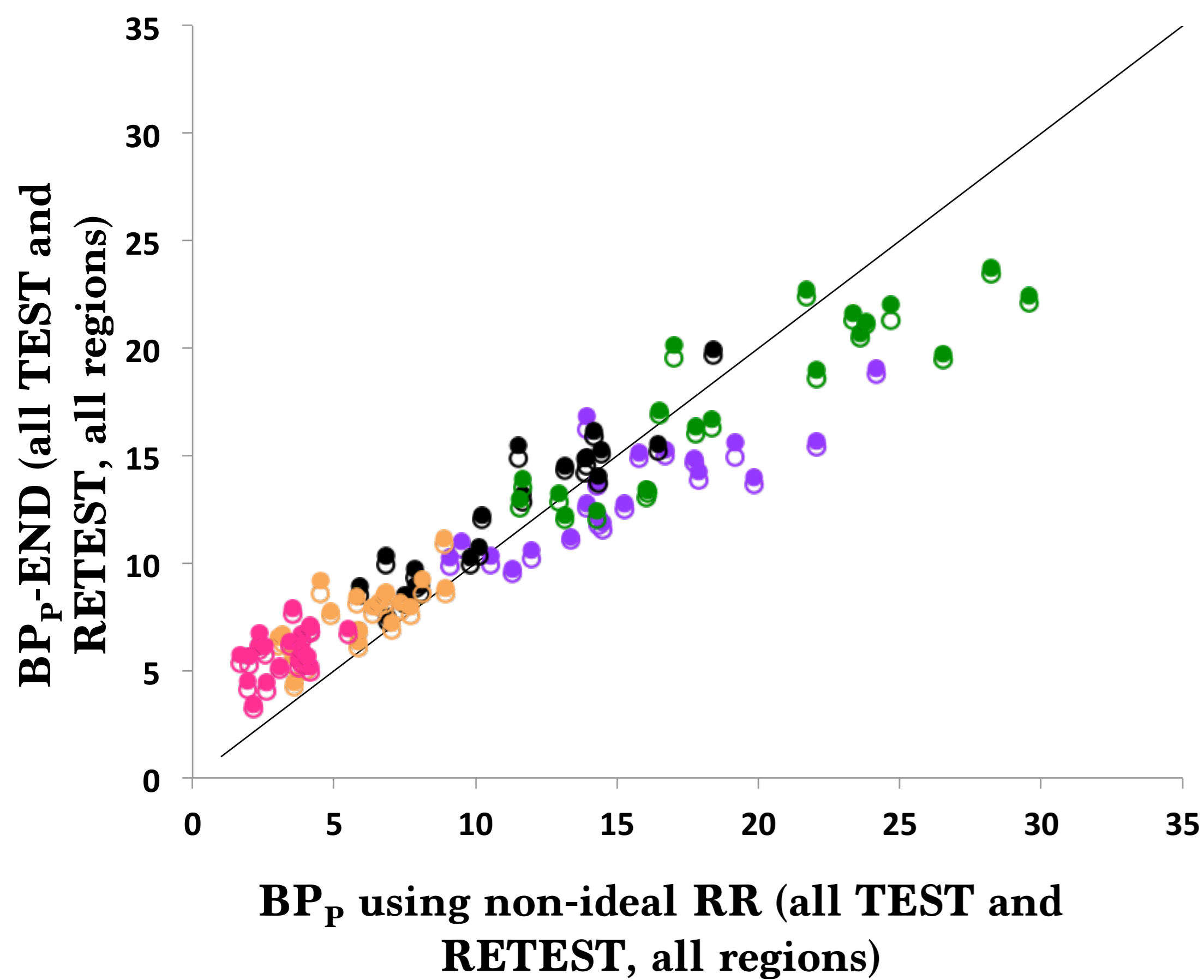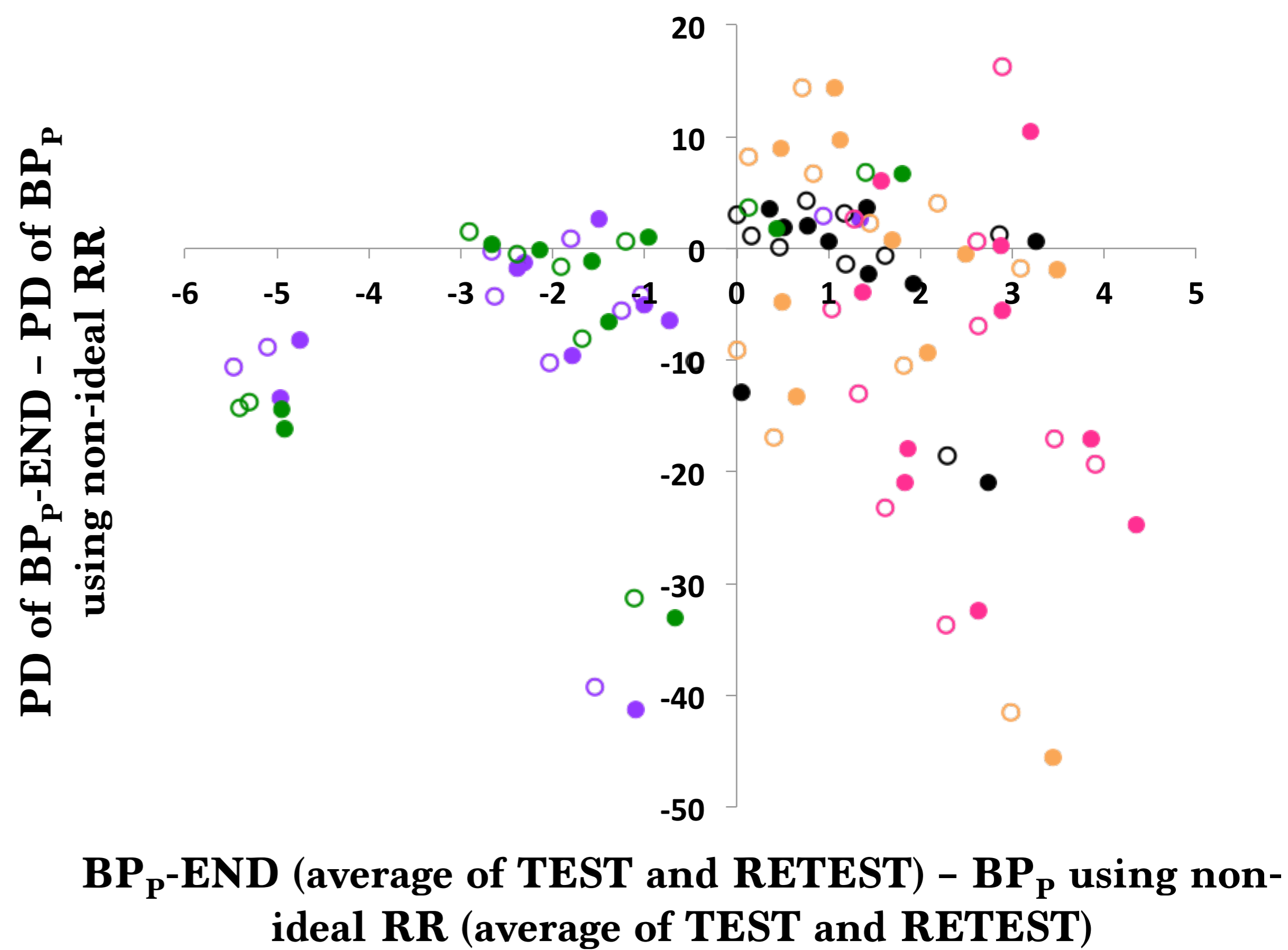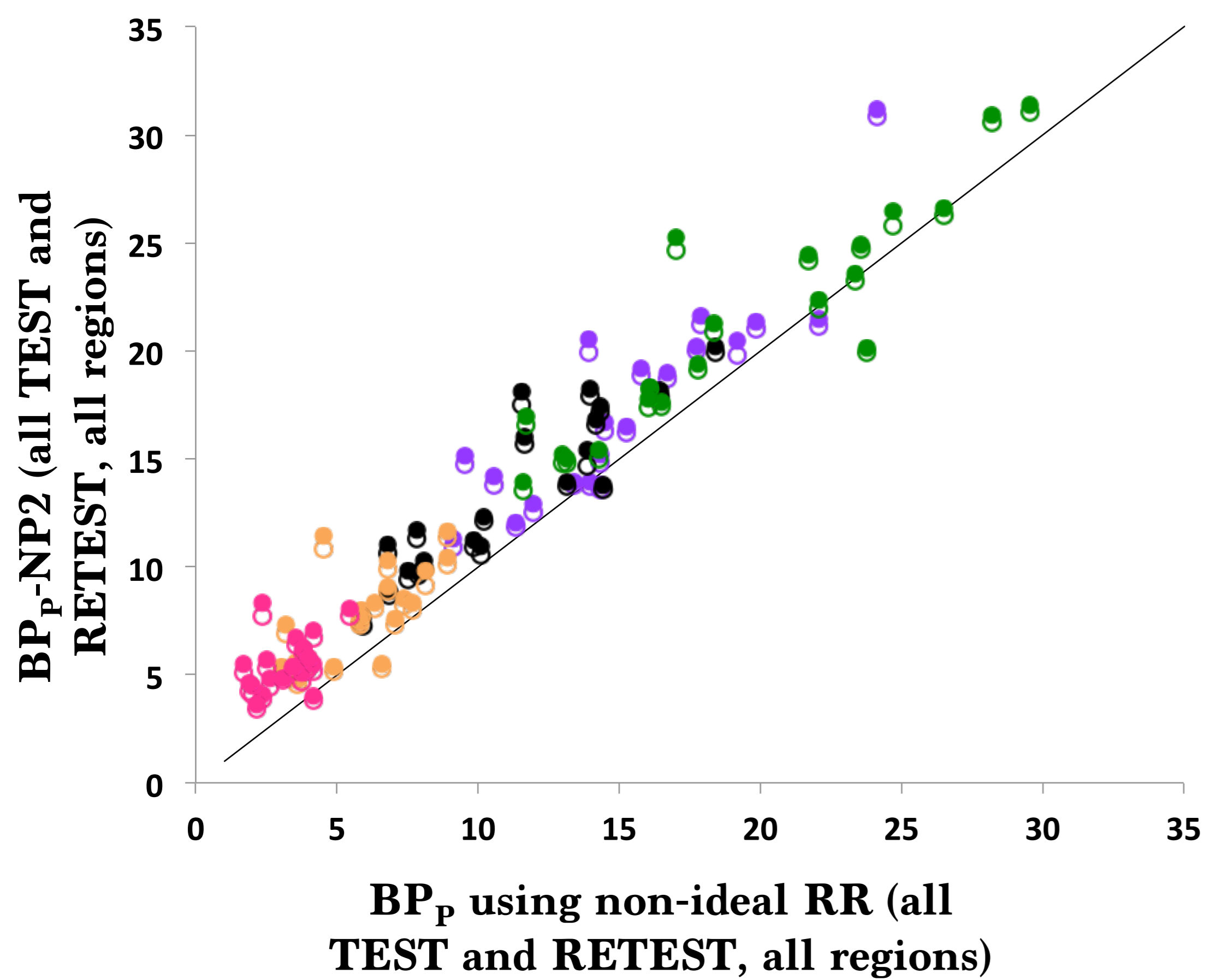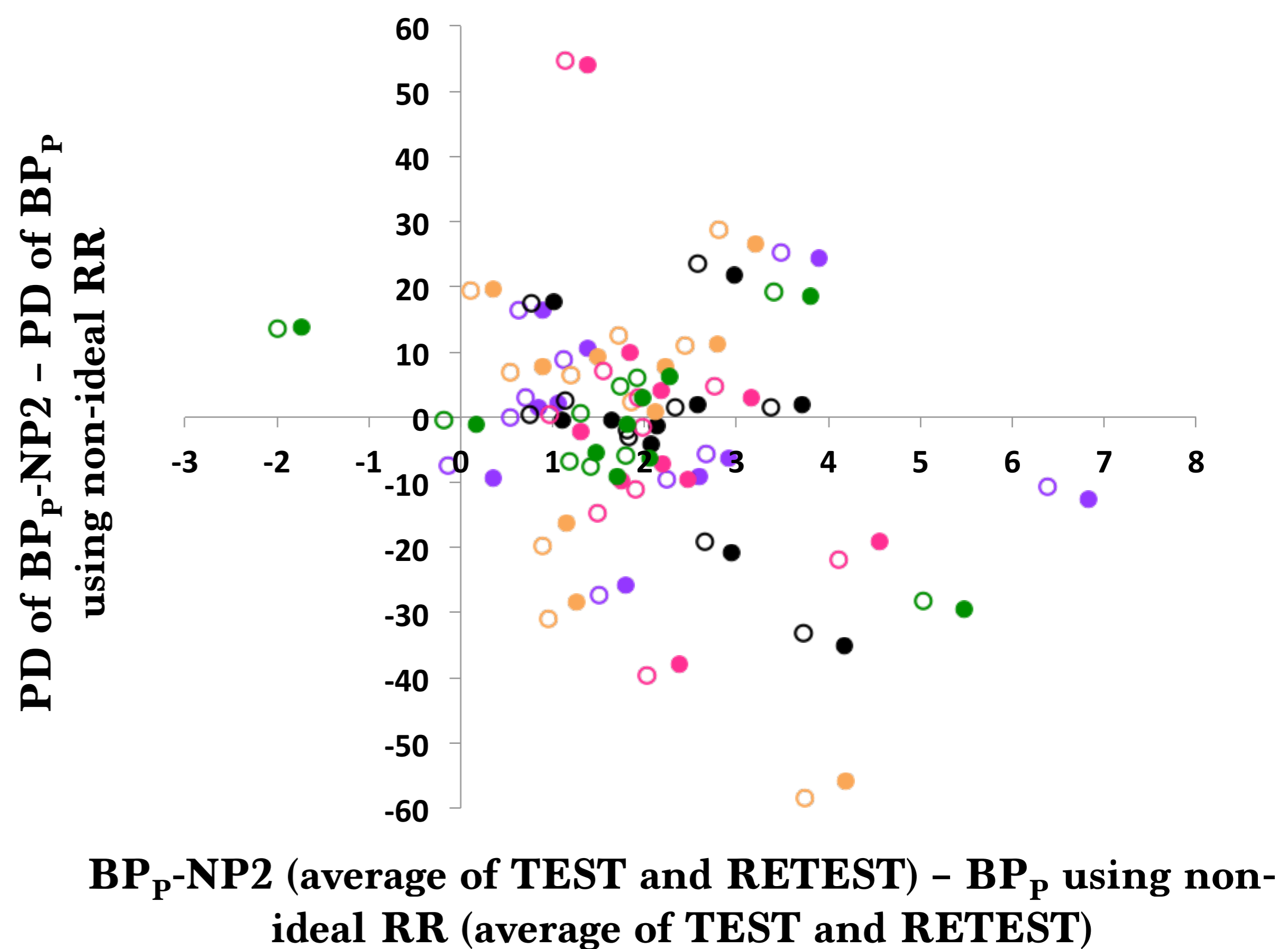

Supplement: S3 Fig — Left: Scatter plots of BPP-HYBRID, BPP-END, and BPP-NP2 (see definitions in S1 and S2 Texts) values versus BPP-RR,LEGA values obtained using the non-ideal reference region and Likelihood Estimation in Graphical Analysis (LEGA). The black solid line is the identity line. Right: Distance between test-retest percent difference (PD) values obtained using BPP-HYBRID and PD values obtained using BPP-RR,LEGA (y axis) versus the corresponding distance between BPP-HYBRID (average of test and re-test) and BPP-RR,LEGA (average of test and re-test). Open circles represent values obtained using the non-displaceable distribution volume (VND) from HYDECA with βopt-S and γopt-S; solid circles represent values obtained using the VND from HYDECA with βopt-B and γopt-B. BPP-RR,LEGA = VT (LEGA)–VT-RR,LEGA; VT (LEGA): tracer total distribution volume (VT) estimated using LEGA; VT-RR,LEGA: VT in the purported reference region estimated using LEGA. (PDF) [file pone.0176636.s003.pdf]

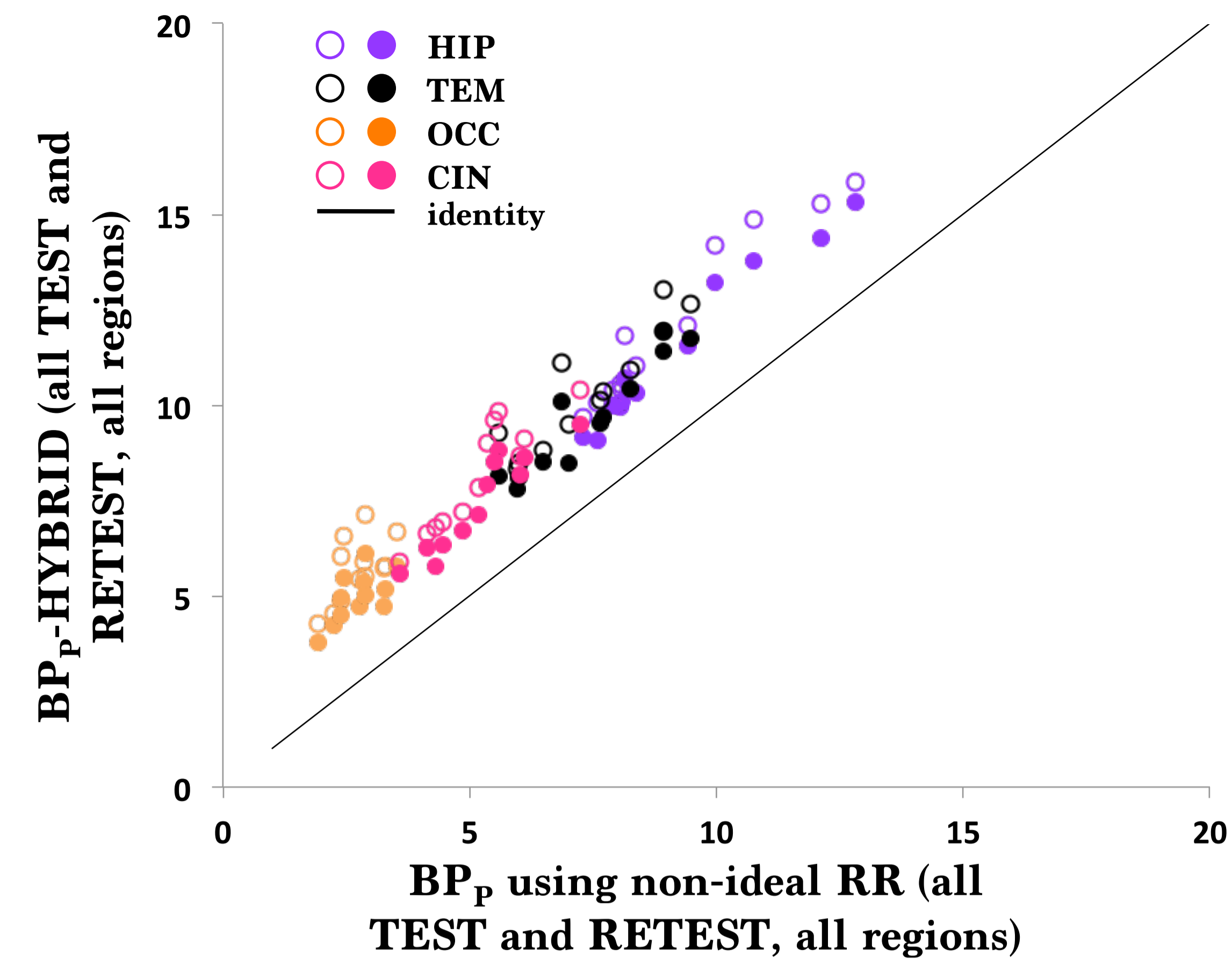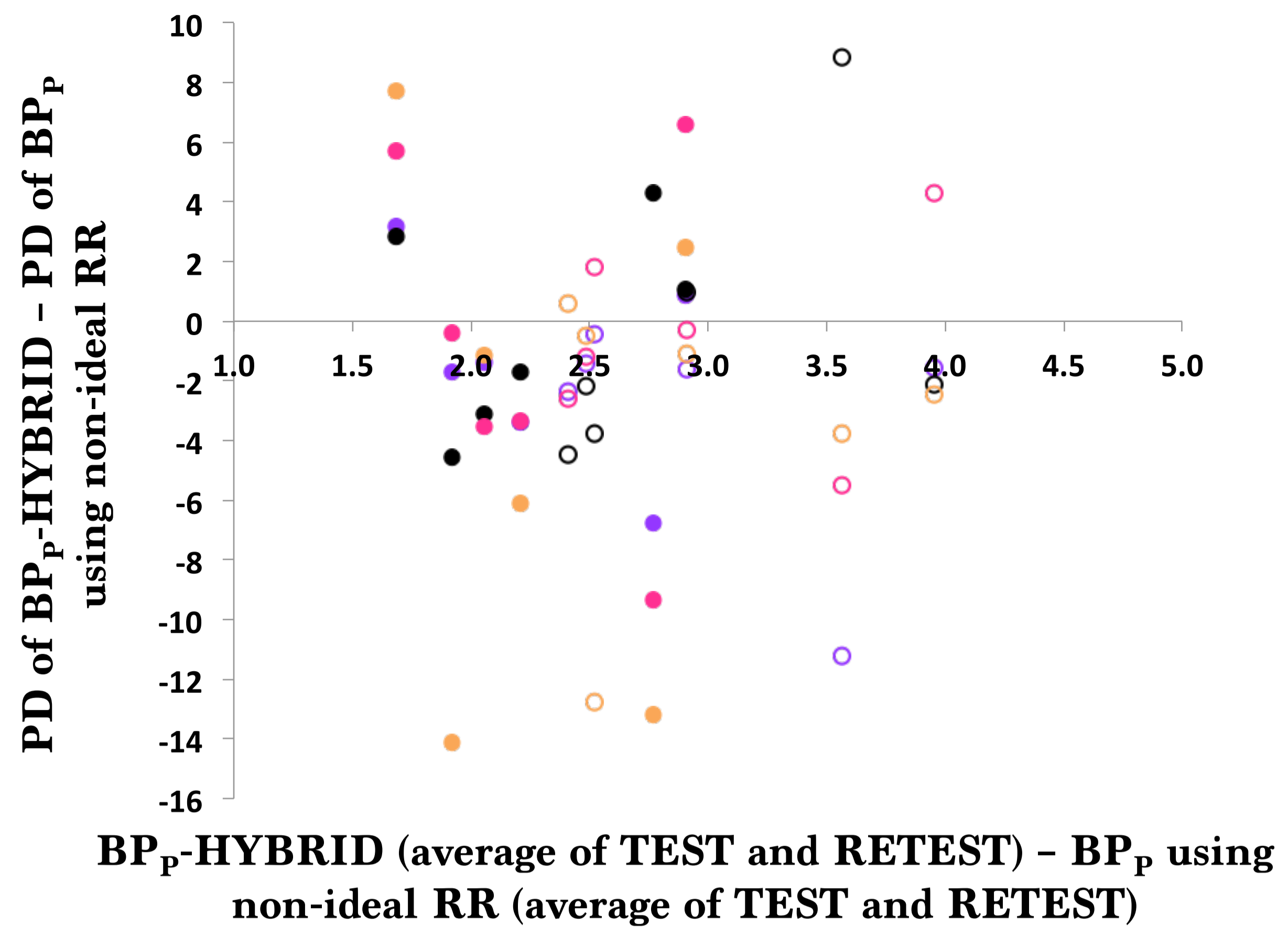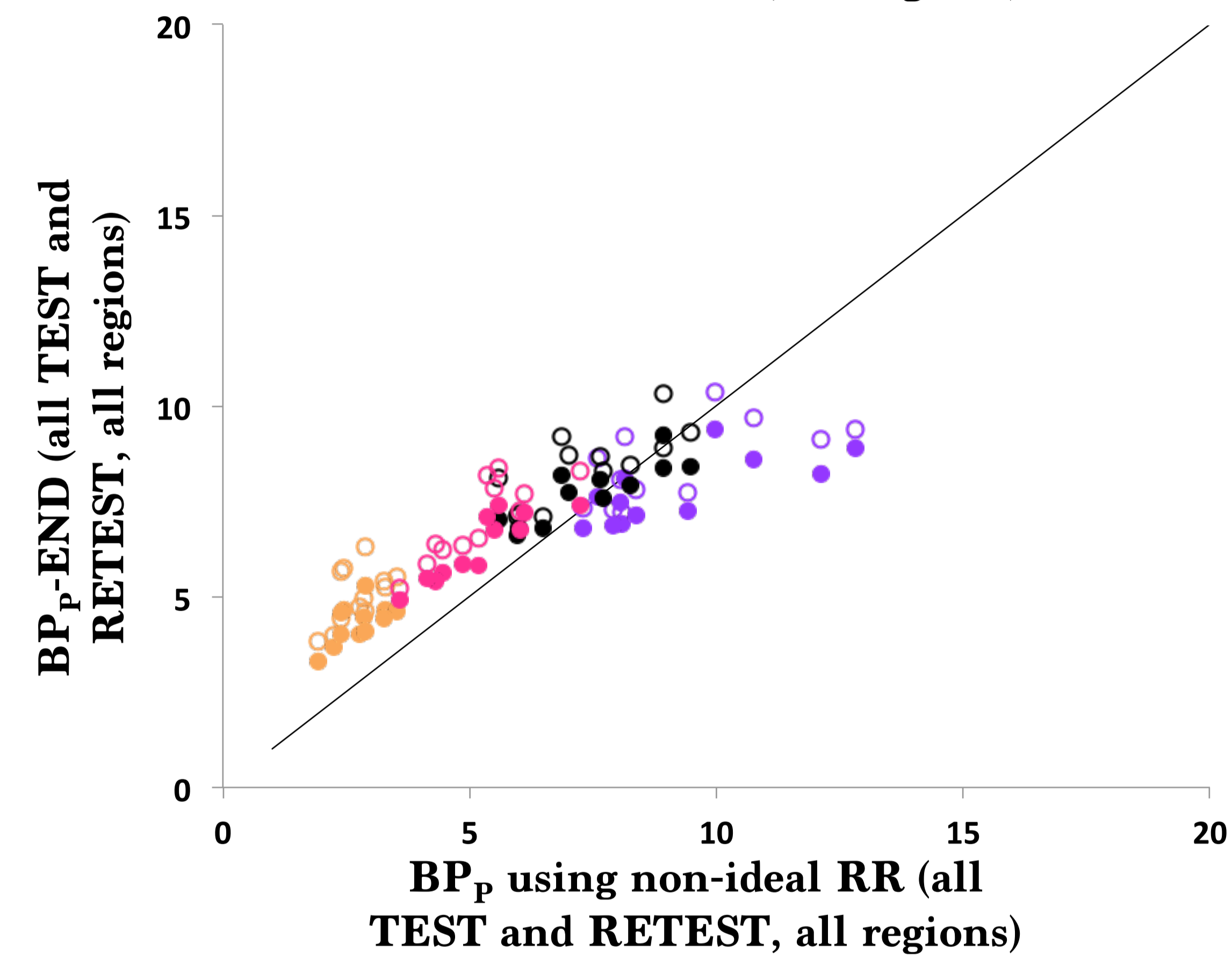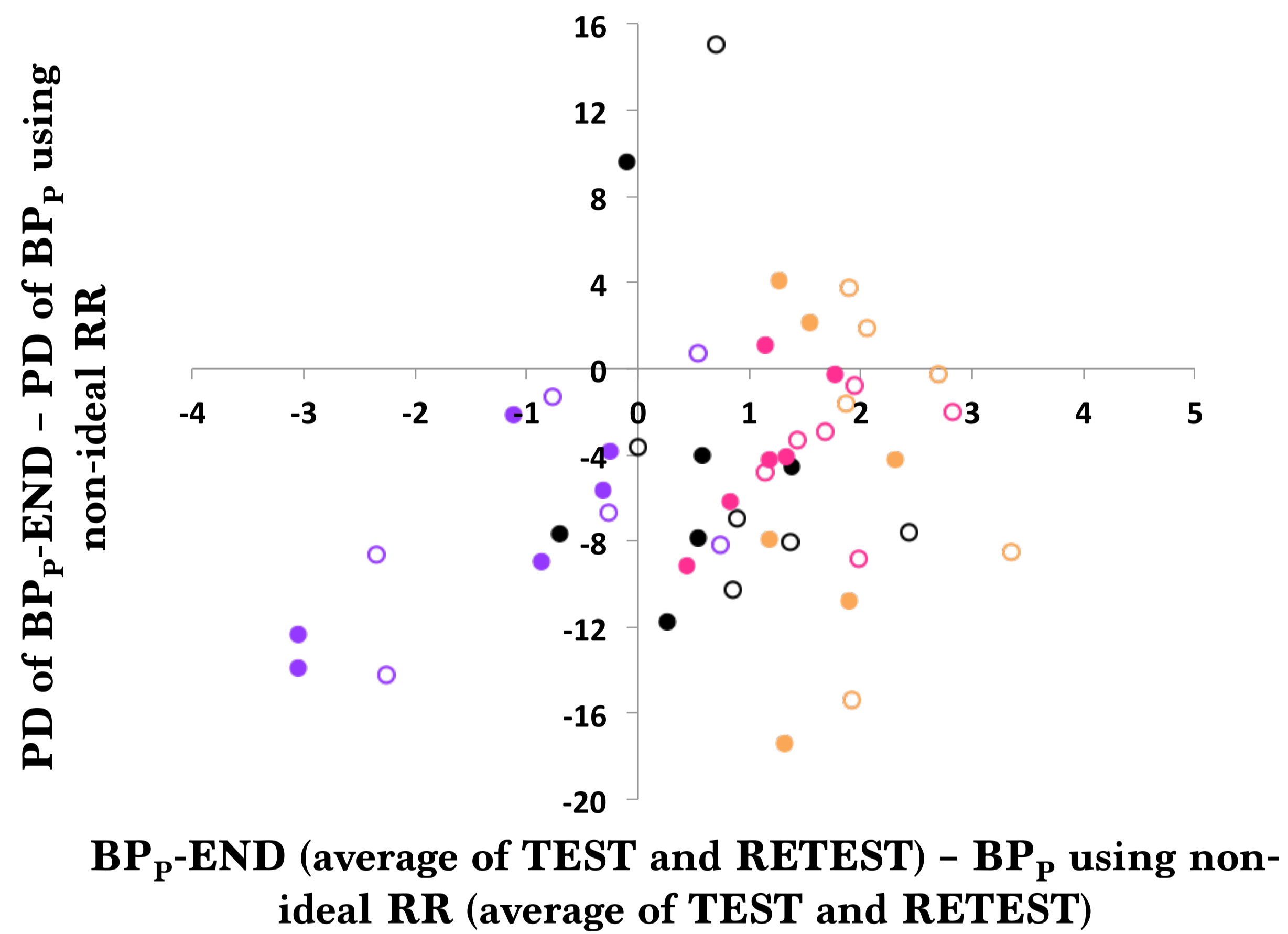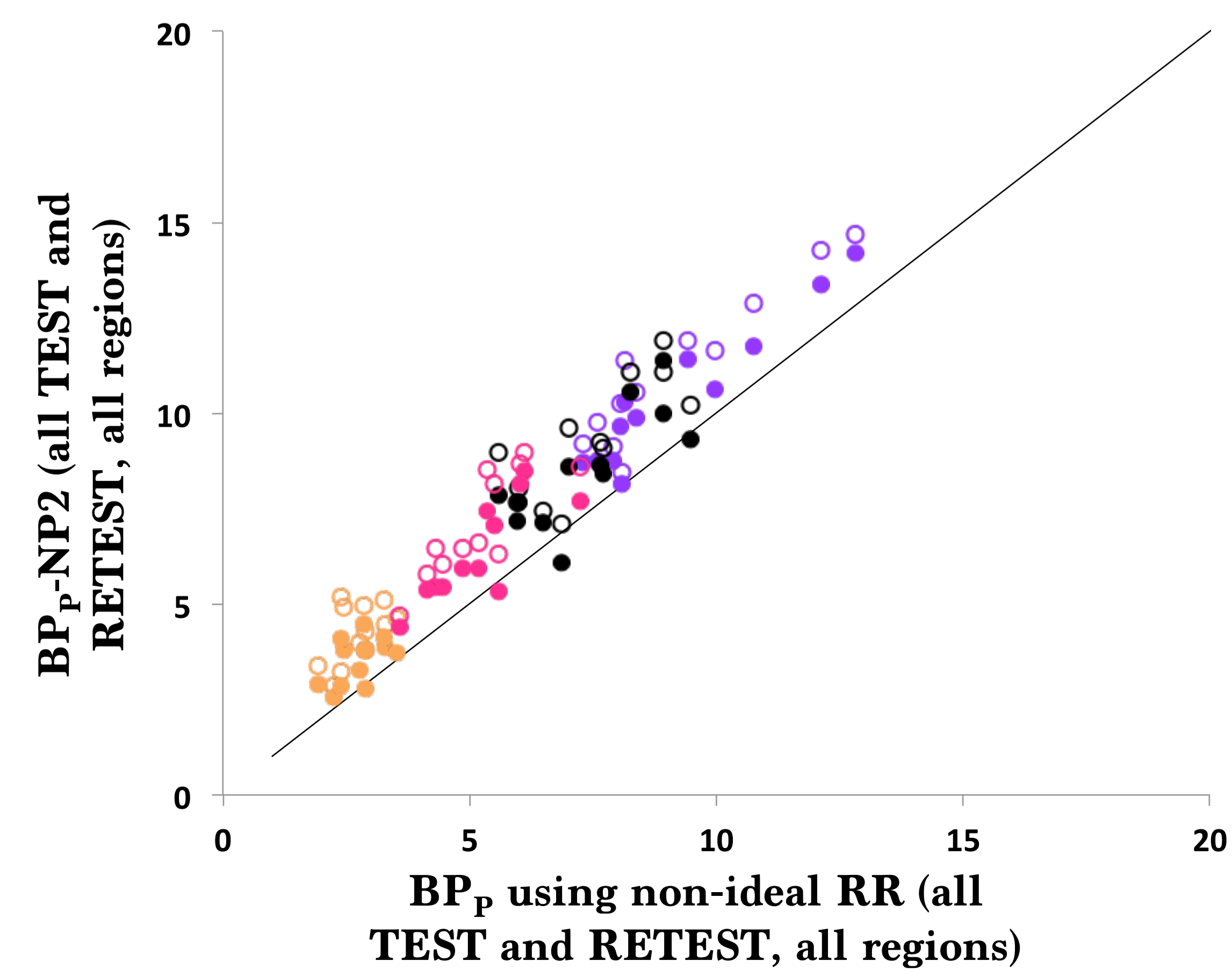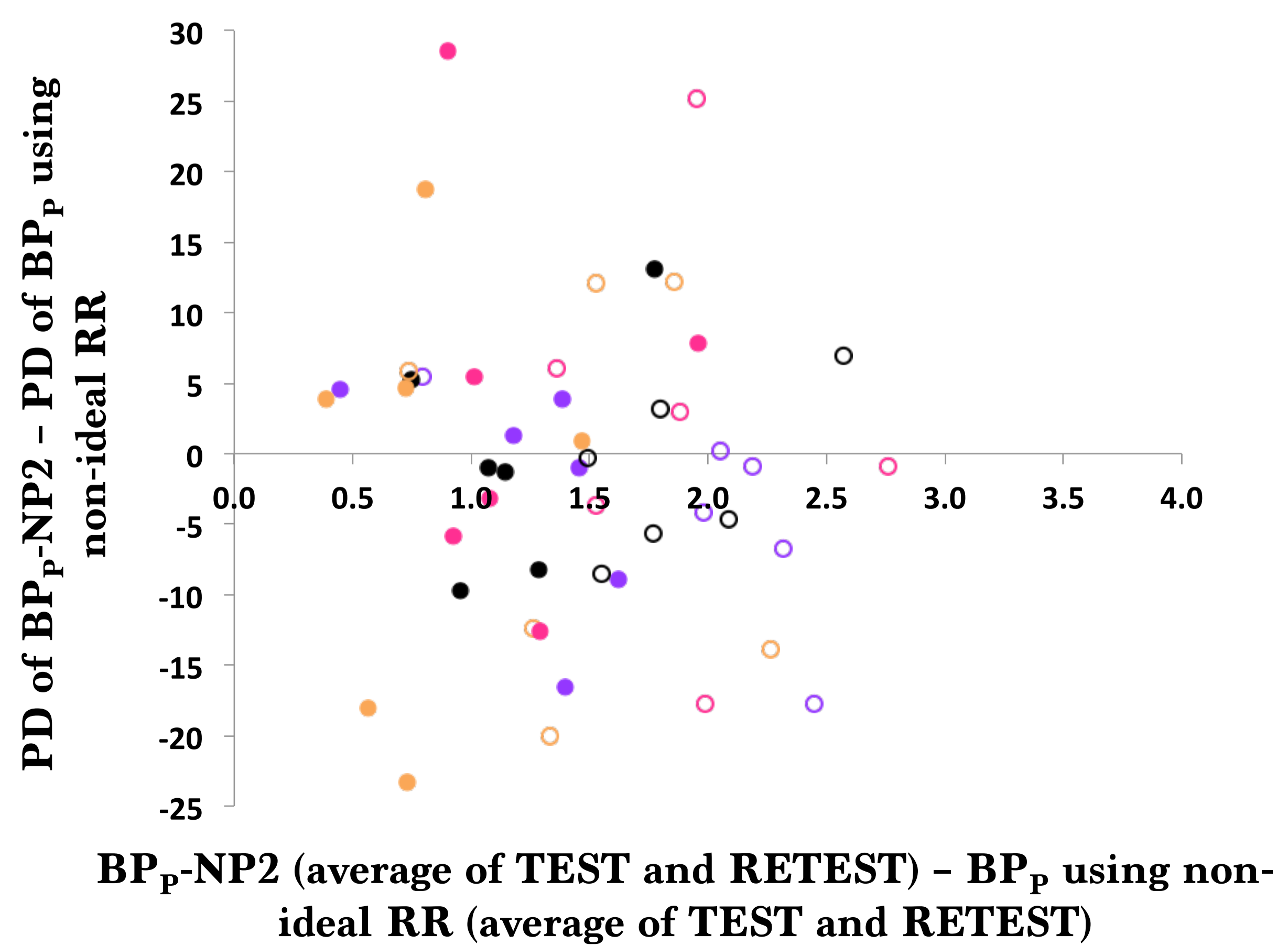

Supplement: S4 Fig — Left: Scatter plots of BPP-HYBRID, BPP-END, and BPP-NP2 (see definitions in S1 and S2 Texts) values versus BPP-RR,LEGA values obtained using the non-ideal reference region and Likelihood Estimation in Graphical Analysis (LEGA). The black solid line is the identity line. Right: Distance between test-retest percent difference (PD) values obtained using BPP-HYBRID and PD values obtained using BPP-RR,LEGA (y axis) versus the corresponding distance between BPP-HYBRID (average of test and re-test) and BPP-RR,LEGA (average of test and re-test). Open circles represent values obtained using the non-displaceable distribution volume (VND) from HYDECA with βopt-S and γopt-S; solid circles represent values obtained using the VND from HYDECA with βopt-B and γopt-B. BPP-RR,LEGA = VT (LEGA)–VT-RR,LEGA; VT (LEGA): tracer total distribution volume (VT) estimated using LEGA; VT-RR,LEGA: VT in the purported reference region estimated using LEGA. (PDF) [file pone.0176636.s004.pdf]

# [<sup>11</sup>C]DASB

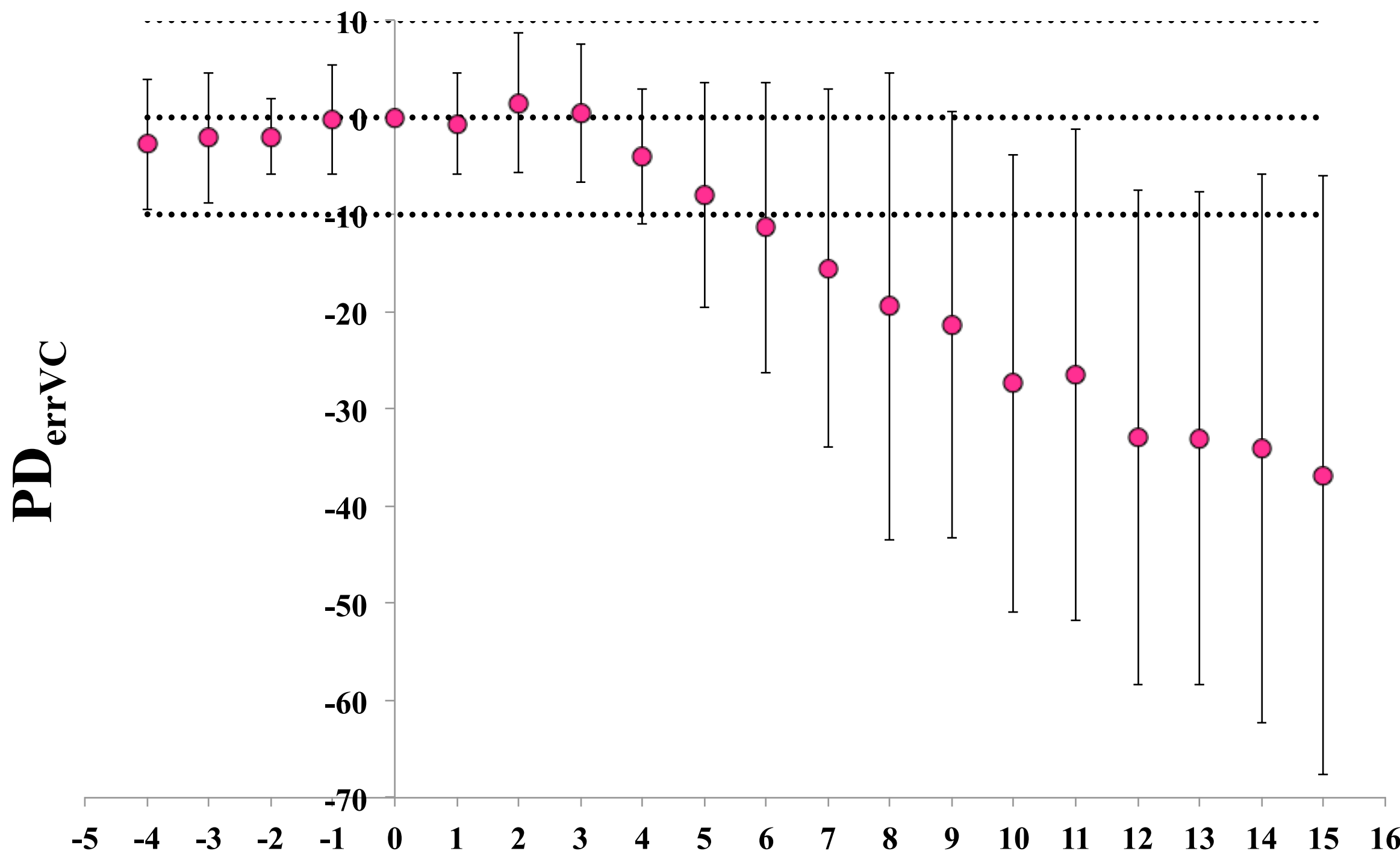

# [<sup>11</sup>C]CUMI-101

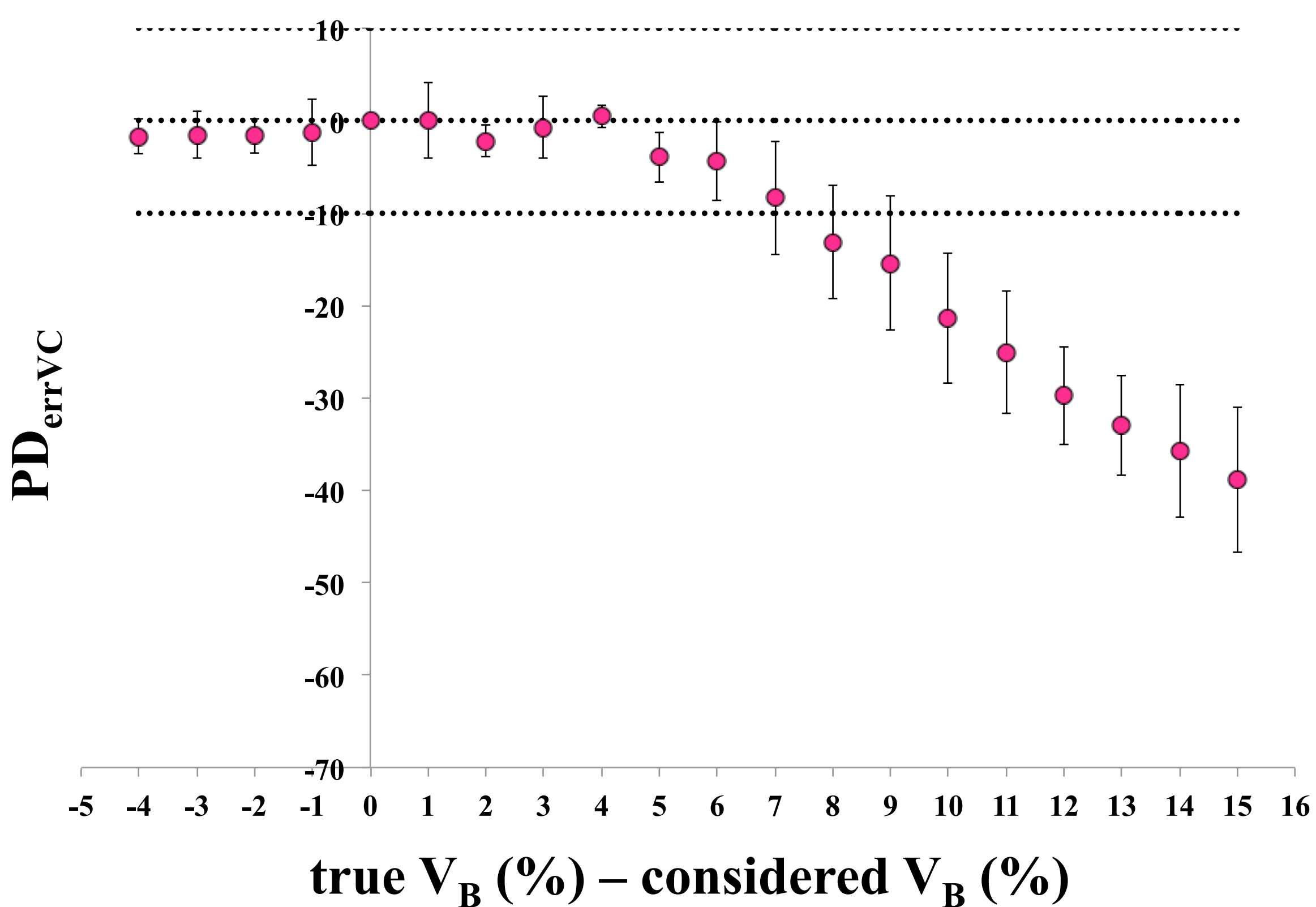

Supplement: S5 Fig — Percent difference (PDerrVC) between the non-displaceable distribution volume (VND) value estimated at each instance of erroneously corrected time activity curves and the VND value estimated in correspondence of the accurately corrected set of time activity curves (y-axis), as a function of the difference between the true fractional blood volume (VB) value and the value adopted for correction (x-axis); dots and error bars indicate average and standard deviation across subjects, respectively, within each tracer. The dotted horizontal lines indicate the +10%, 0%, and -10% mark, respectively. (PDF) [file pone.0176636.s005.pdf]

# [<sup>11</sup>C]CUMI-101

CGM

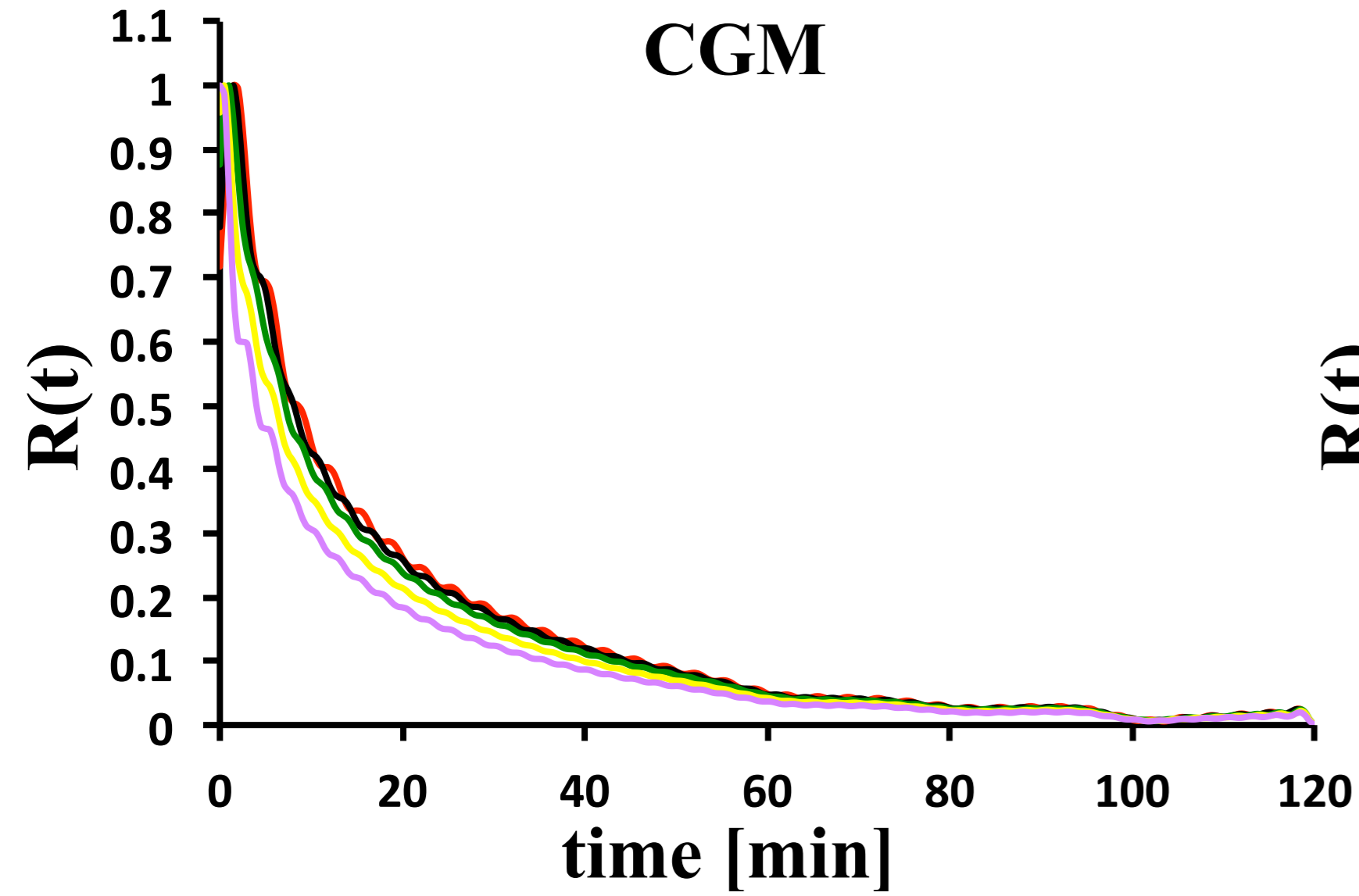

HIP

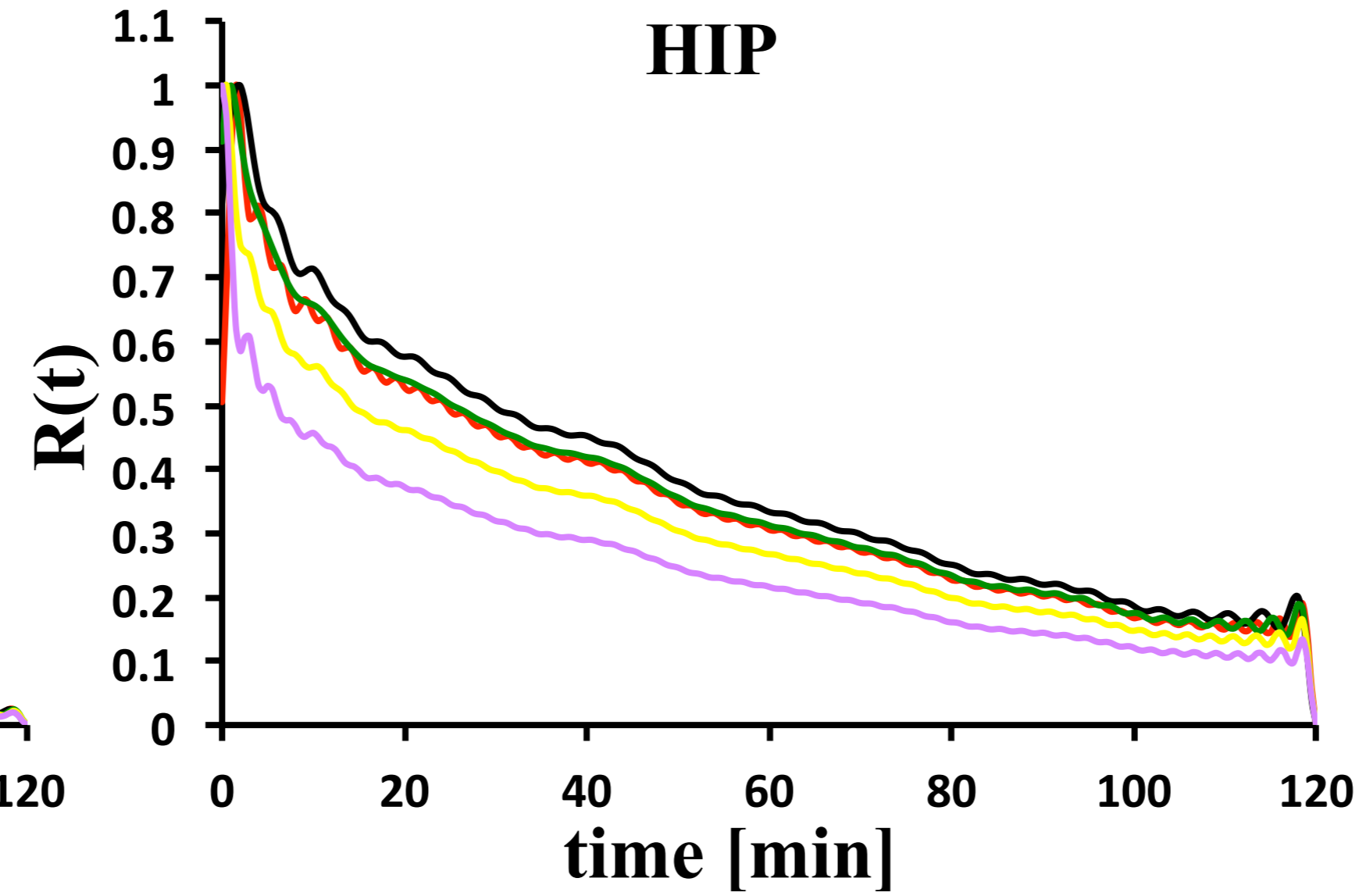

TEM

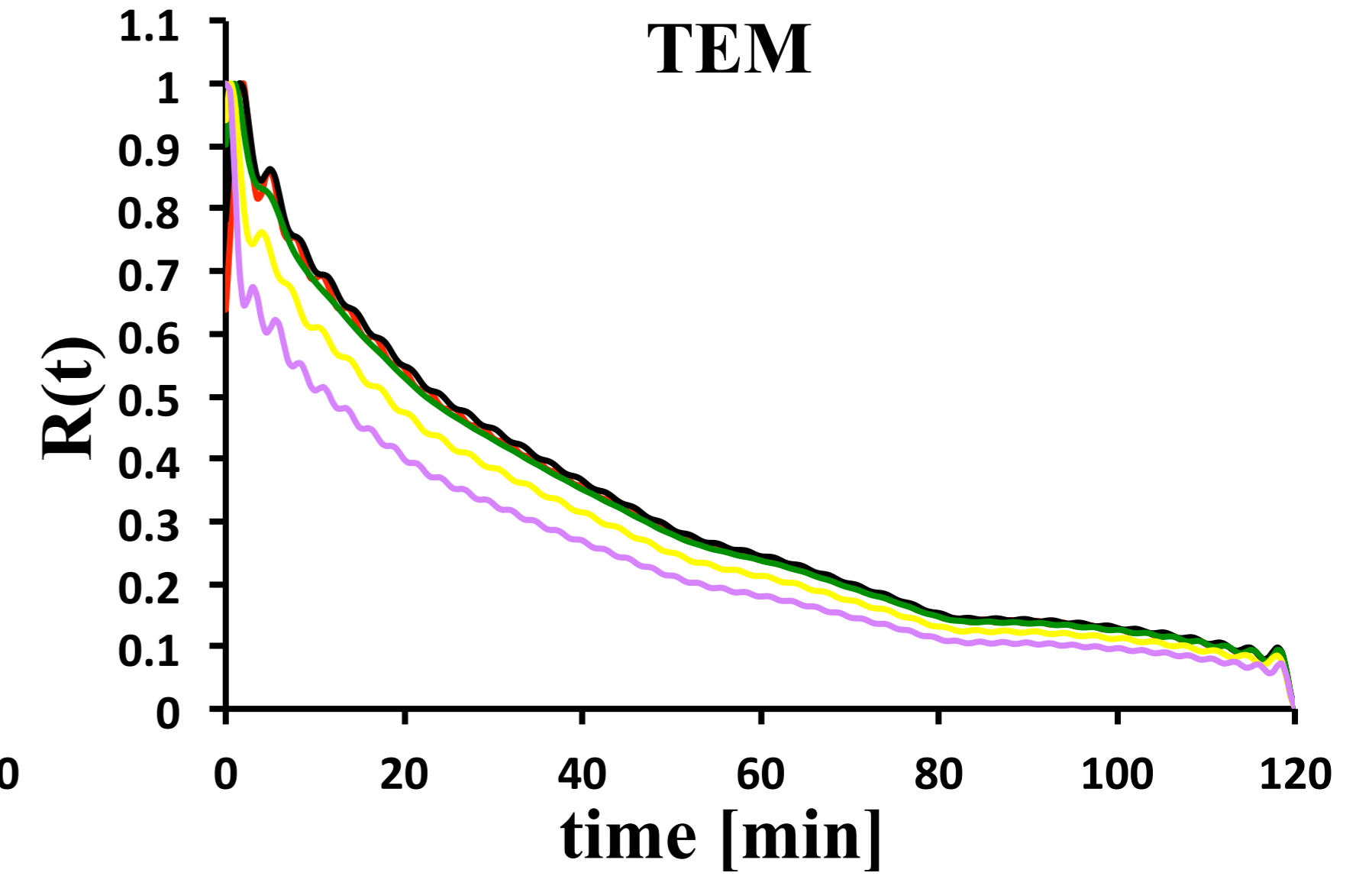

OCC

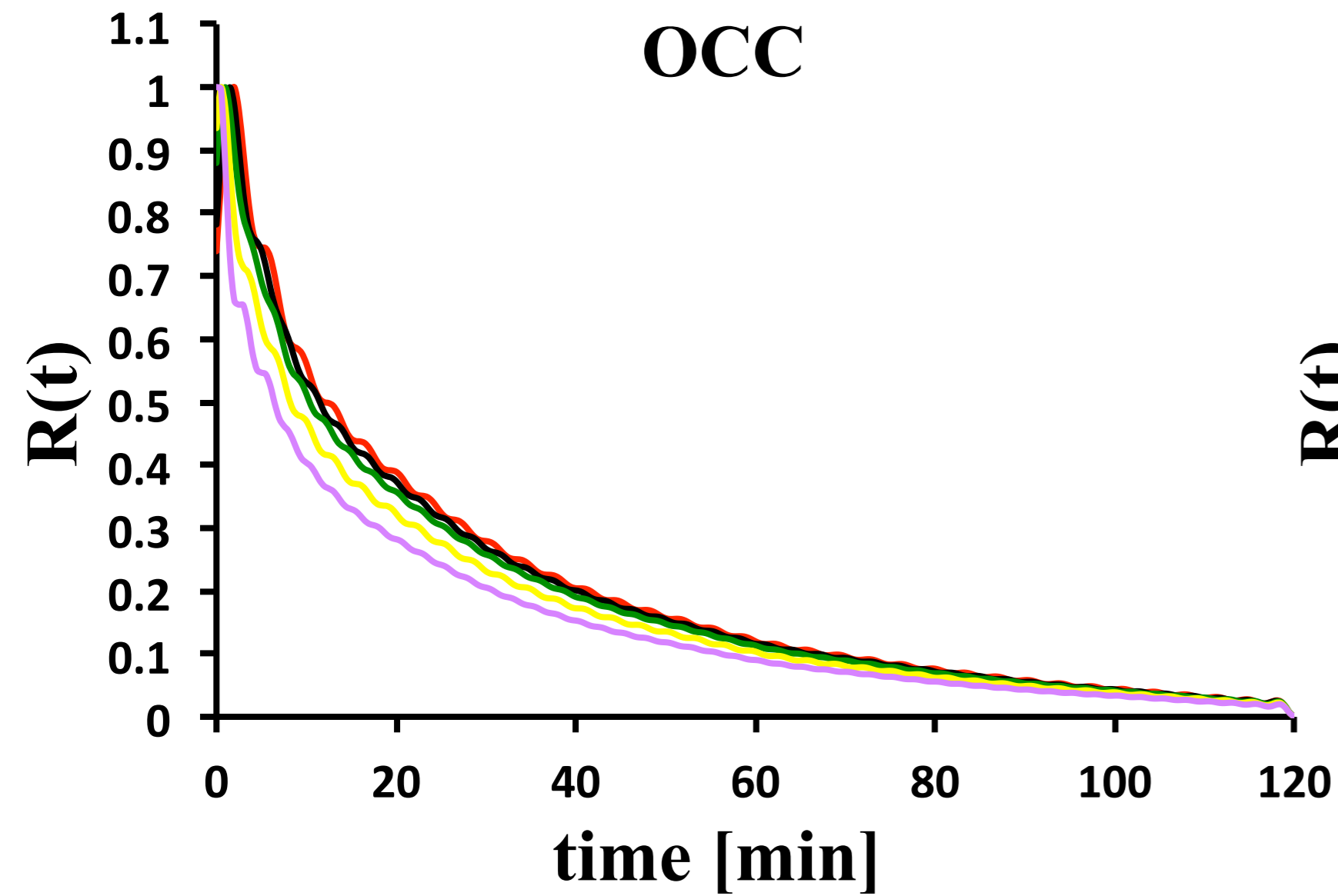

CIN

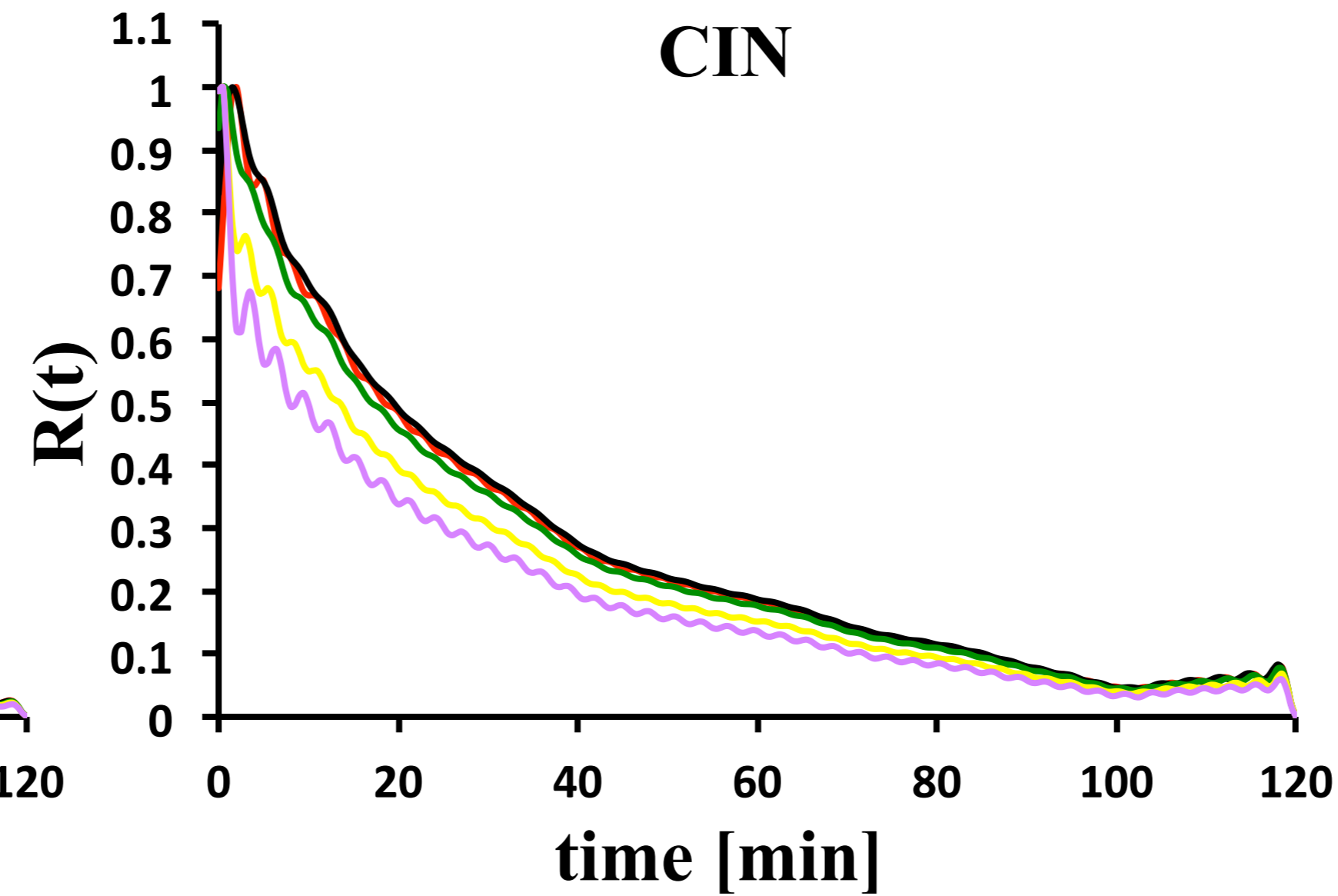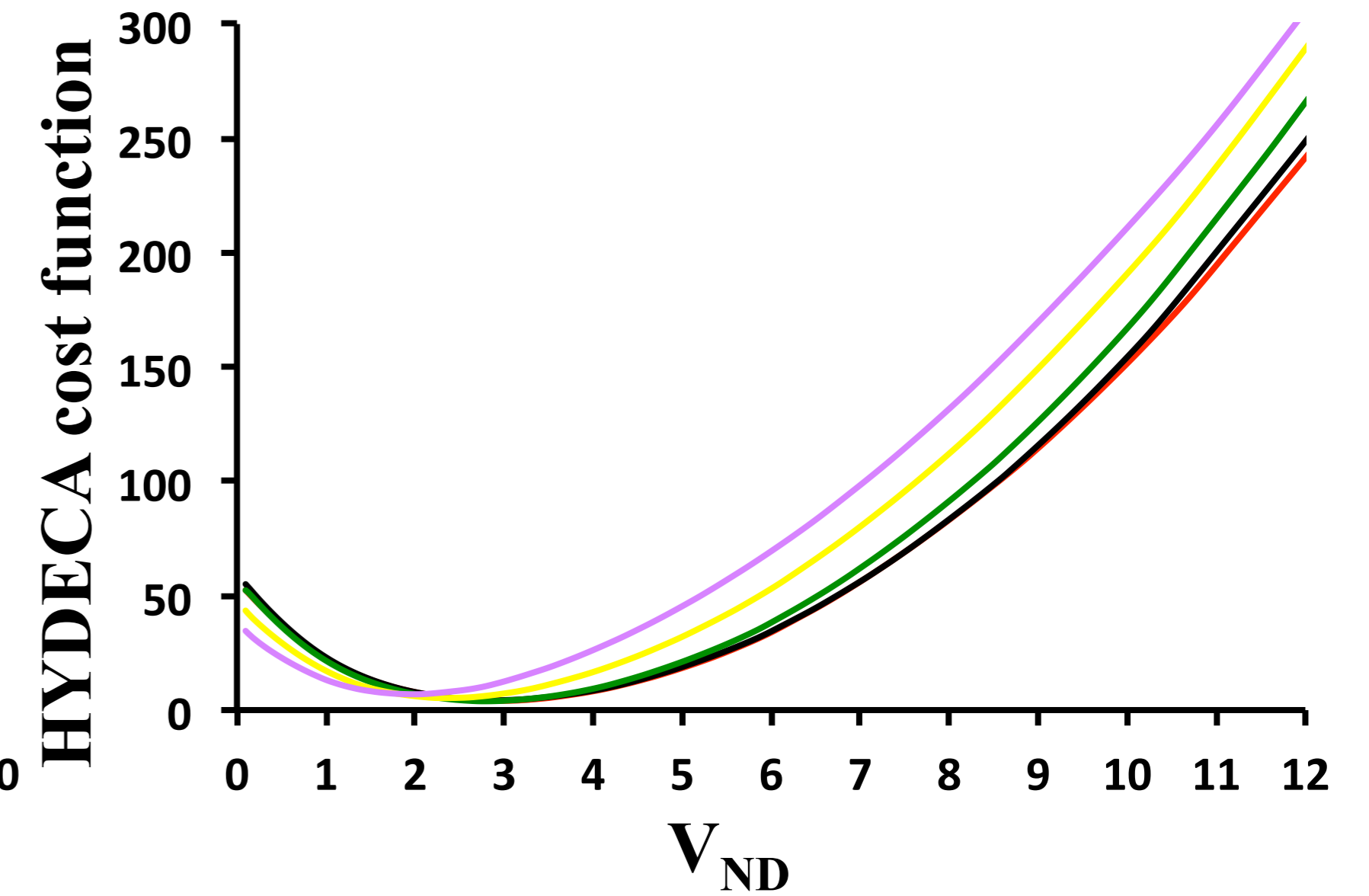

—  $V_B$  error = -4%    —  $V_B$  error = 0%    —  $V_B$  error = 5%    —  $V_B$  error = 10%    —  $V_B$  error = 15%

Supplement: S6 Fig — Residue function curves R(t) in correspondence of different errors and no error in the fractional blood volume value (VB), and the corresponding HYDECA cost functions, in a representative subject for [11C]CUMI-101. CIN: cingulate; HIP: hippocampus; OCC: occipital lobe; TEM: temporal lobe; CGM: cerebellum grey matter. (PDF) [file pone.0176636.s006.pdf]

**[<sup>11</sup>C]DASB**

**CGM –  $V_{\text{ND}} = 3$**

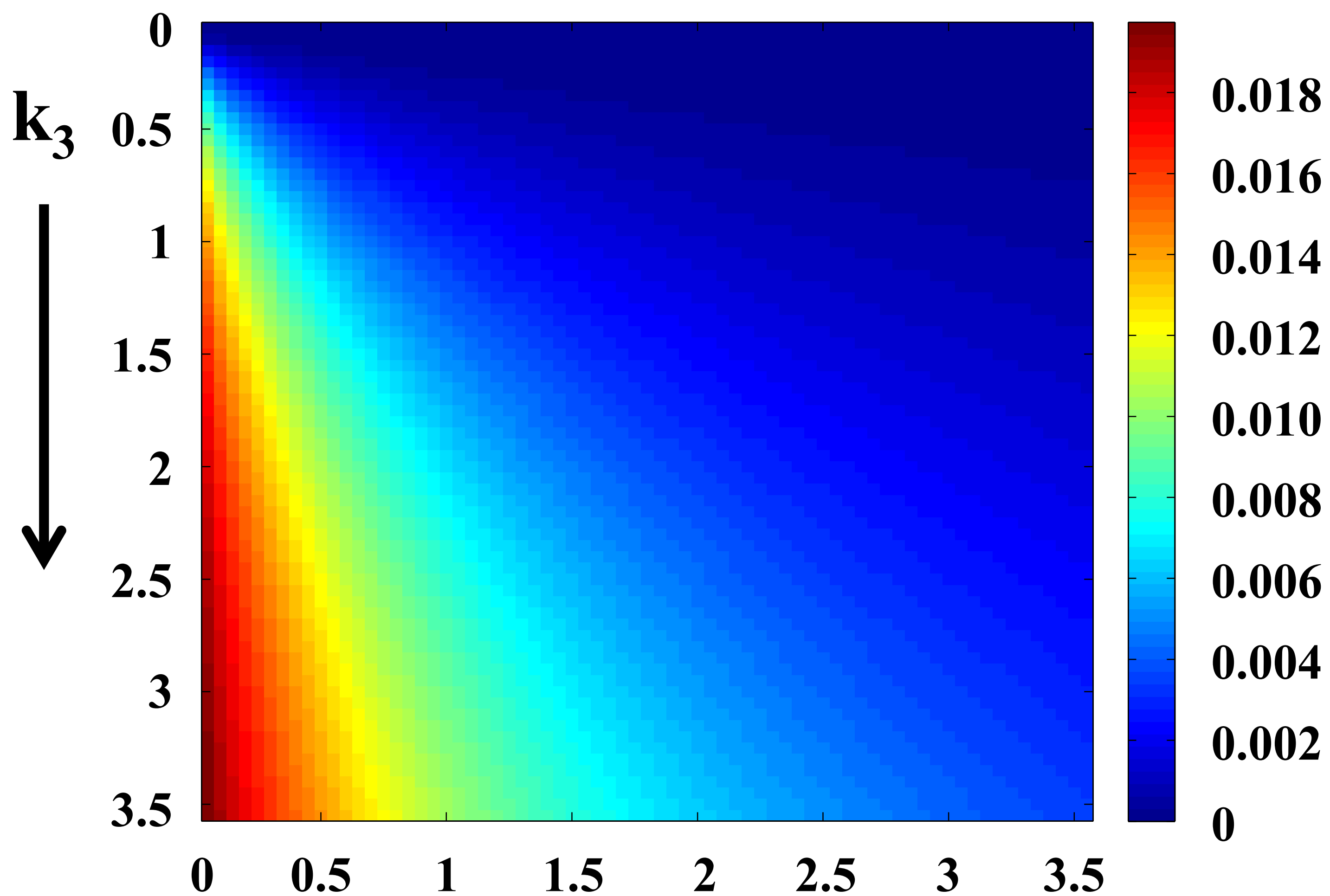

**VST –  $V_{\text{ND}} = 3$**

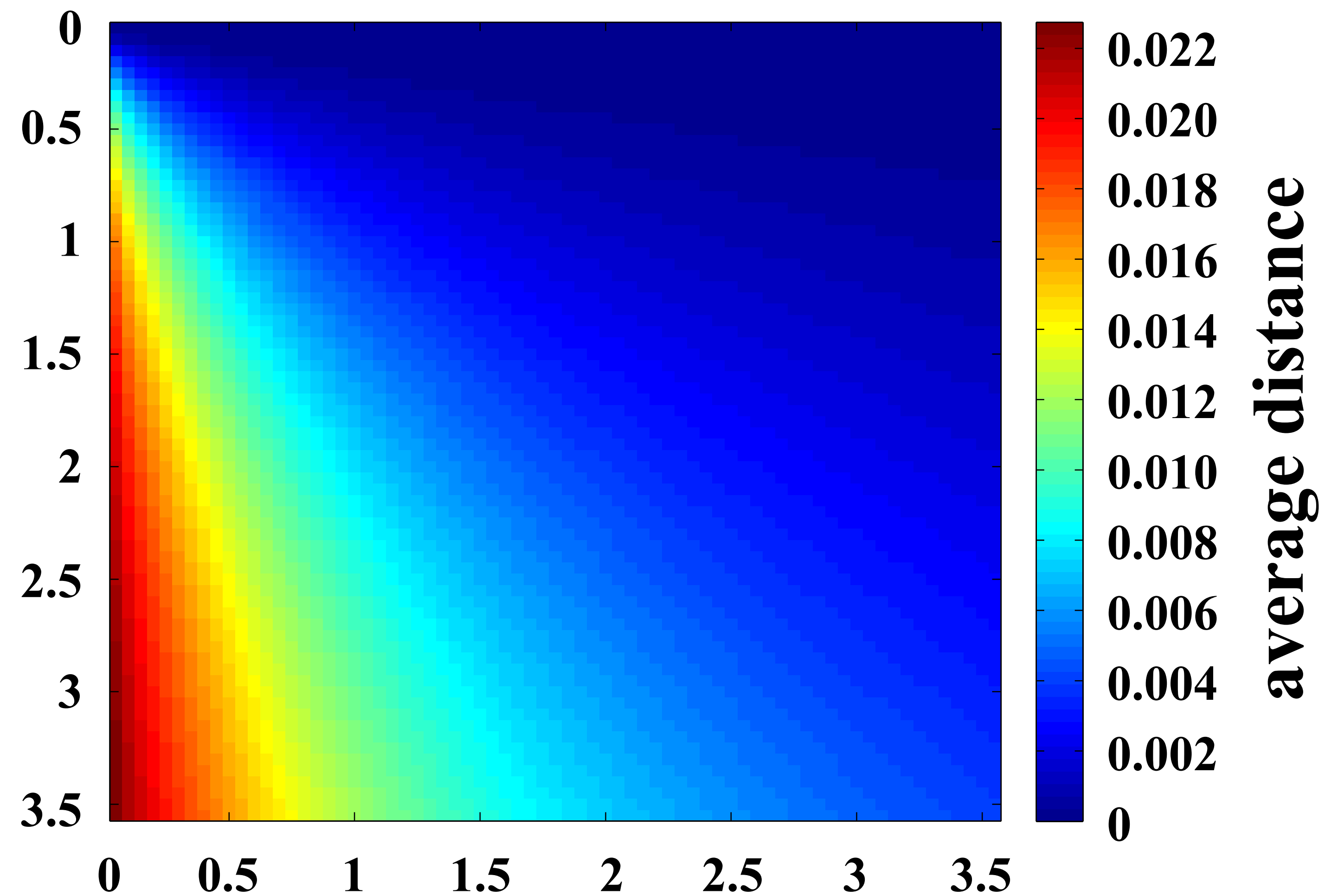

**CGM –  $V_{\text{ND}} = 5$**

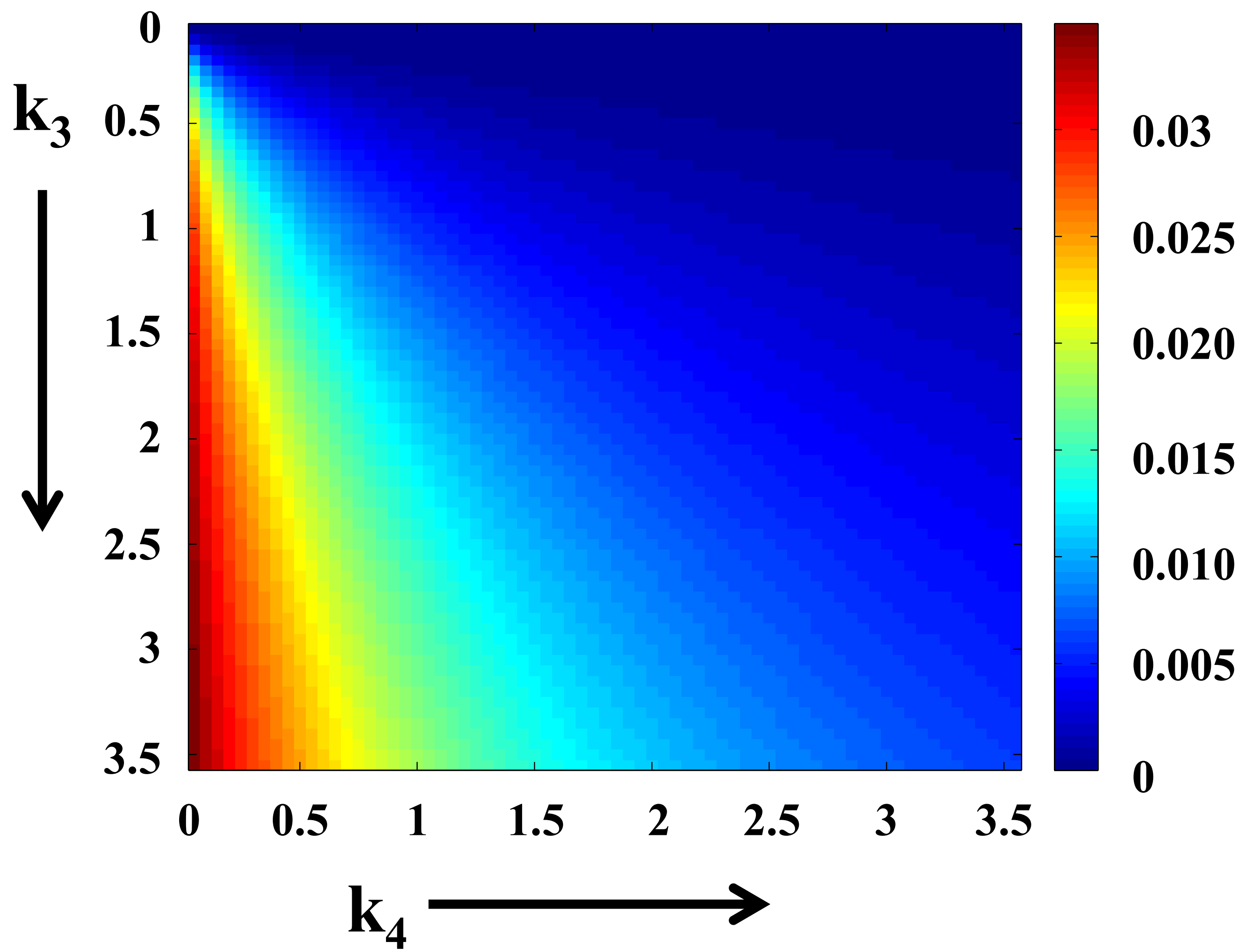

**VST –  $V_{\text{ND}} = 5$**

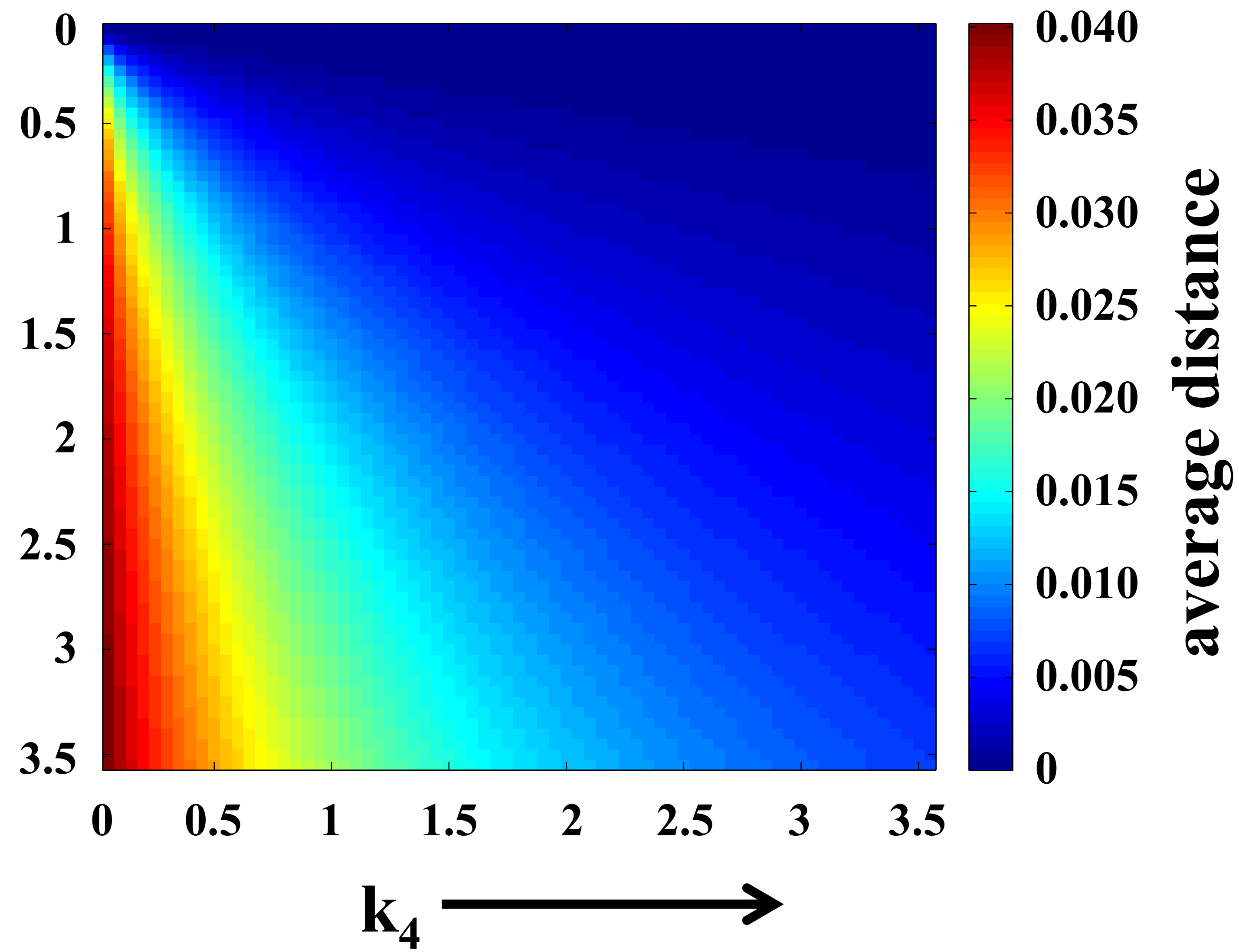

Supplement: S8 Fig — Average (across time points) square distance between the residue function non-displaceable component, RND(t) (see S4 Text), with k3 and k4 >0, and RND(t) with k3 = k4 = 0 as k3 and k4 vary, in 4 cases of (K1, k2) for [11C]DASB. VST: ventral striatum; CGM: cerebellum grey matter. K1, k2 k3 and k4: kinetic rate parameters of a two-tissue compartment model. (PDF) [file pone.0176636.s008.pdf]

**[<sup>11</sup>C]CUMI-101**

**CGM –  $V_{\text{ND}} = 3$**

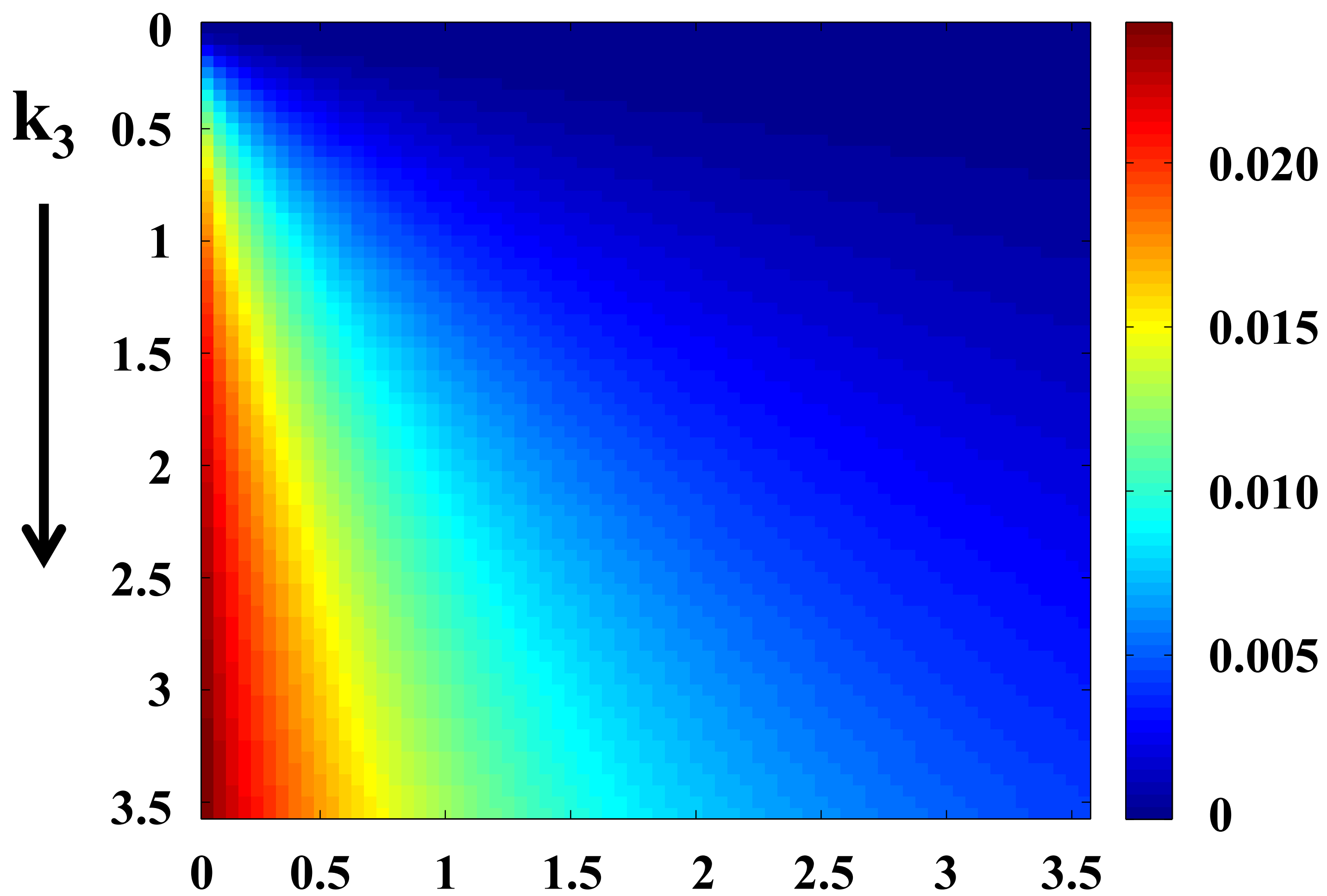

**HIP –  $V_{\text{ND}} = 3$**

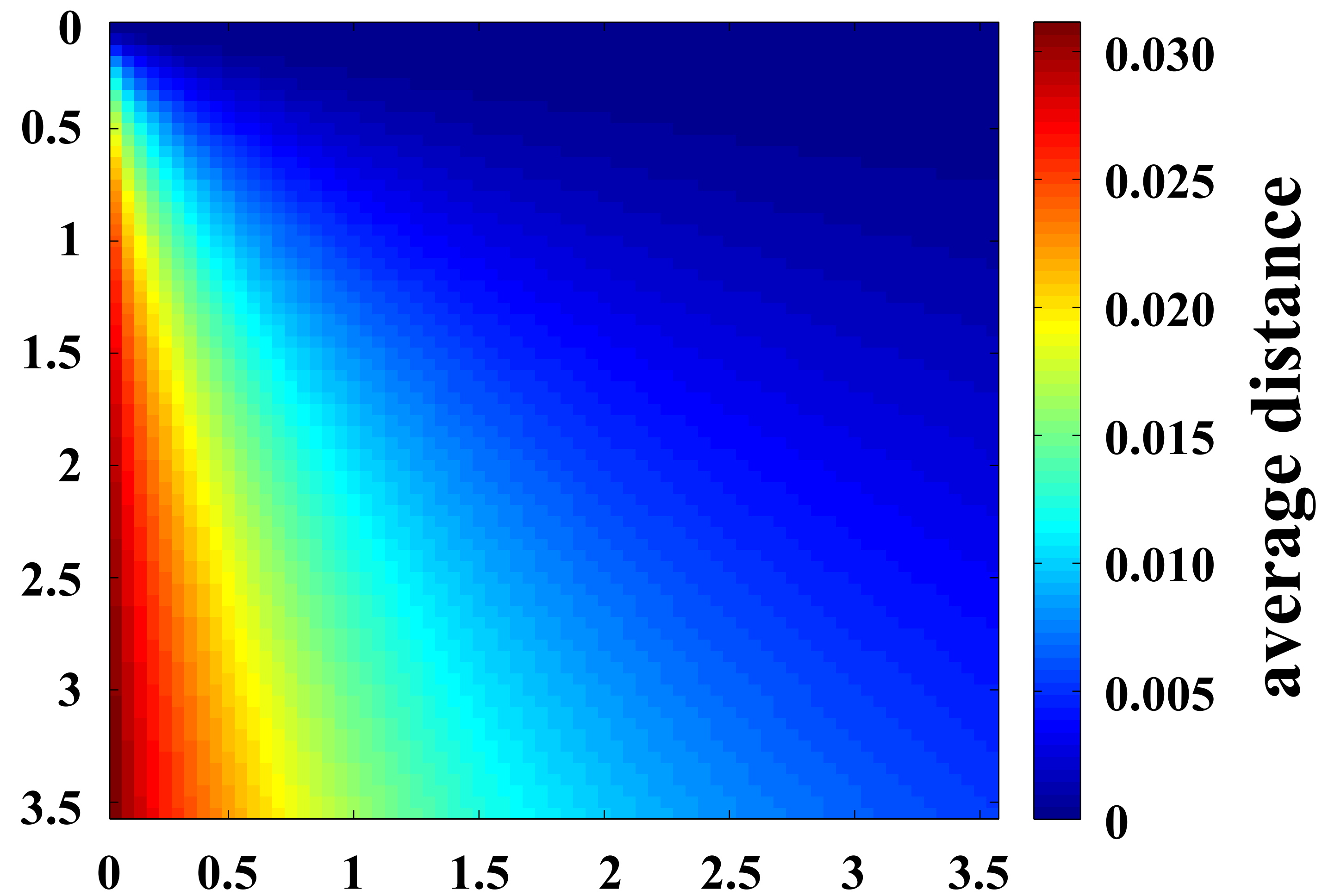

**CGM –  $V_{\text{ND}} = 5$**

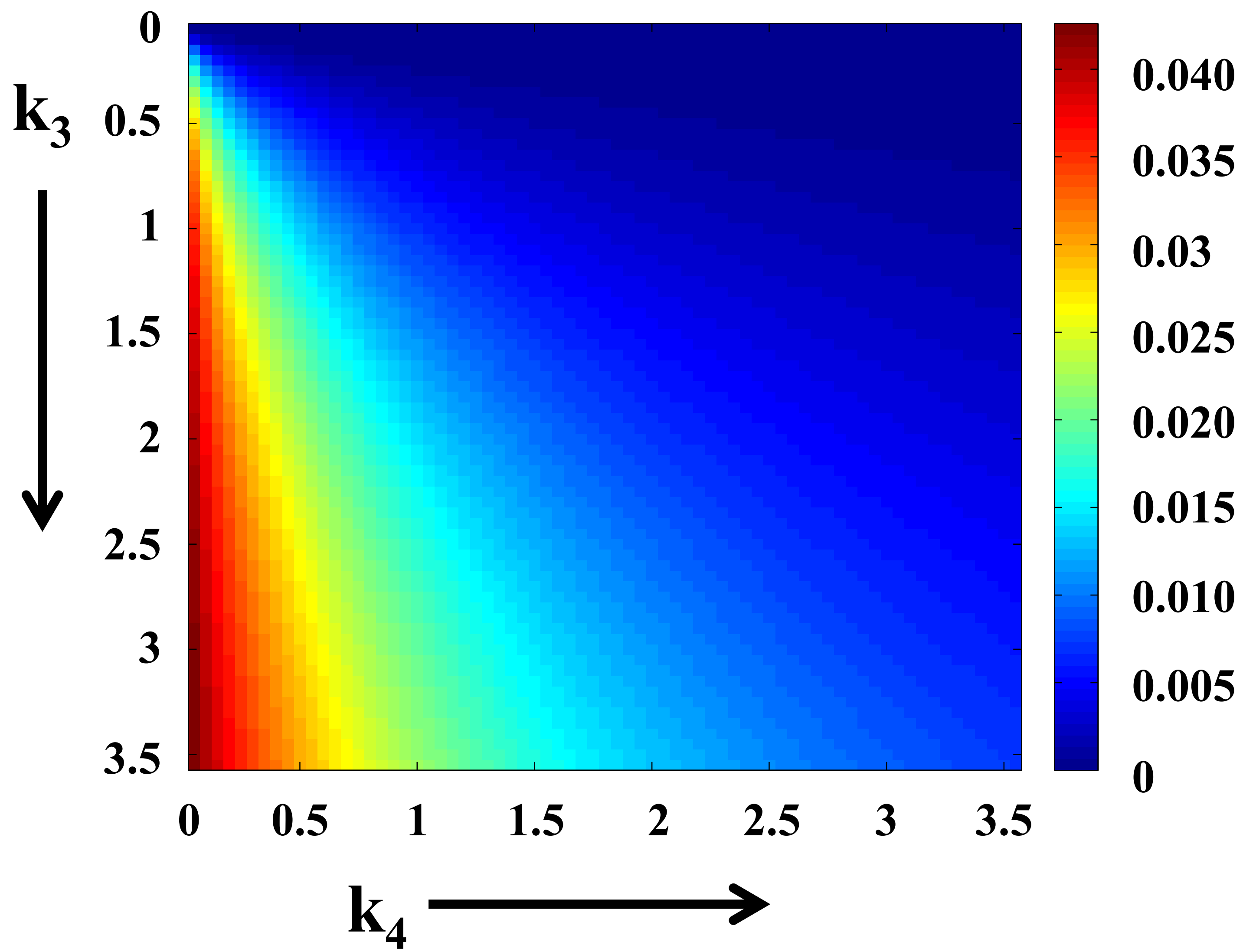

**HIP –  $V_{\text{ND}} = 5$**

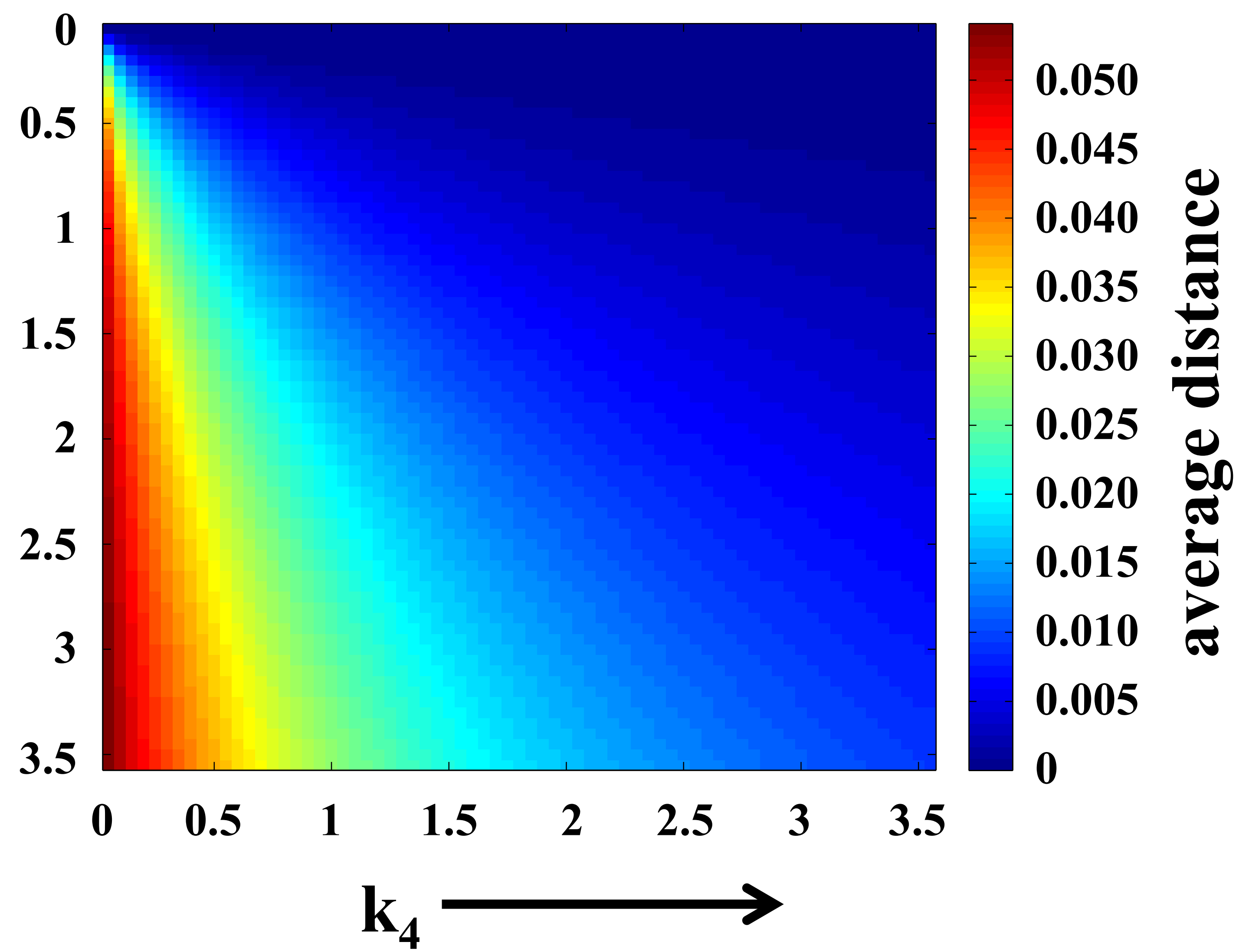

Supplement: S9 Fig — Average (across time points) square distance between the residue function non-displaceable component, RND(t) (see S4 Text), with k3 and k4 >0, and RND(t) with k3 = k4 = 0 as k3 and k4 vary, in 4 cases of (K1, k2) for [11C]CUMI-101. HIP: hippocampus; CGM: cerebellum grey matter. K1, k2 k3 and k4: kinetic rate parameters of a two-tissue compartment model. (PDF) [file pone.0176636.s009.pdf]
