# Supplementary material for: A hybrid deconvolution approach for estimation of in vivo non-displaceable binding for brain PET targets without a reference region
Source: PLoS One. 2017 May 1;12(5):e0176636. doi: 10.1371/journal.pone.0176636 (PMC5411064; doi:10.1371/journal.pone.0176636)
Supplement: S7 Fig — Residue function curves R(t) in correspondence of different errors and no error in the fractional blood volume value (VB), and the corresponding HYDECA cost functions, in a representative subject for [11C]DASB. AMY: amygdala; DCA: dorsal caudate; HIP: hippocampus; TEM: temporal lobe; VST: ventral striatum; CGM: cerebellum grey matter. (PDF) [file pone.0176636.s007.pdf]

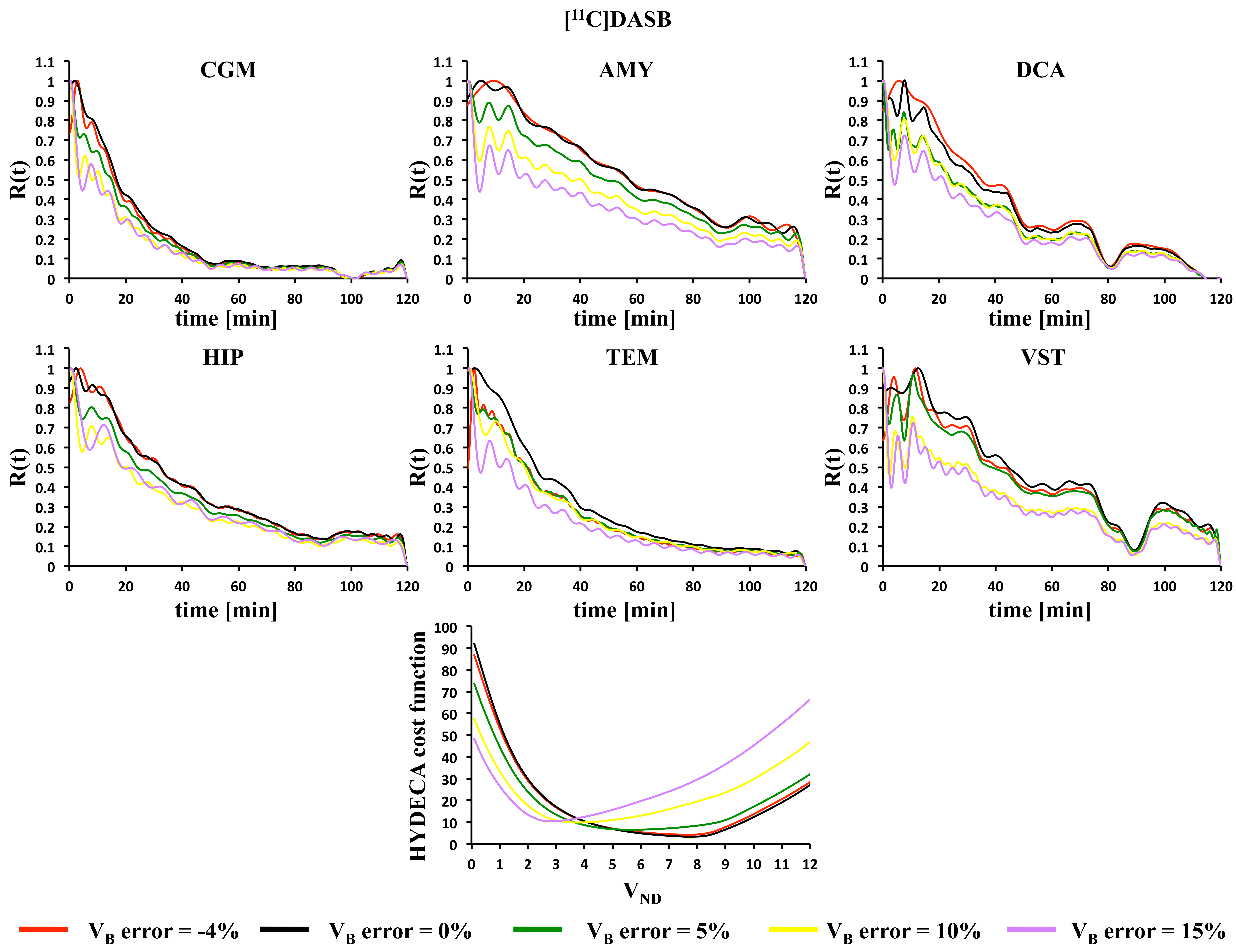

[11C]DASB
CGM
AMY
DCA
HIP
TEM
VST
R(t)
time [min]
HYDECA cost function
V_ND
V_B error = -4%
V_B error = 0%
V_B error = 5%
V_B error = 10%
V_B error = 15%
