# Supplementary material for: A hybrid deconvolution approach for estimation of in vivo non-displaceable binding for brain PET targets without a reference region
Source: PLoS One. 2017 May 1;12(5):e0176636. doi: 10.1371/journal.pone.0176636 (PMC5411064; doi:10.1371/journal.pone.0176636)
Supplement: S1 Text — Supporting information and equations accompanying S1 and S2 Figs. (PDF) [file pone.0176636.s010.pdf]

Alternative nonparametric binding potentials and their test-retest reproducibility

As the non-displaceable distribution volume (V_{ND}) is ultimately estimated in order to calculate the tracer binding potentials to the target of interest, we considered two available test-retest datasets with [^{11}C]DASB [1] (10 subjects, 20 scans) and [^{11}C]CUMI-101 [2] (6 subjects, 12 scans) and checked the reproducibility characteristics of binding potentials derived using HYDECA versus using the purported reference region. While the results related to the binding potentials $BP_{P\text{-}HYBRID}$ and $BP_{ND\text{-}HYBRID}$ [3] are reported in the main manuscript, here we report results related to alternative binding potentials that can be derived when performing nonparametric quantification of the Positron Emission Tomography (PET) data [4, 5].

Specifically, in all scans and regions, we also calculated:

$$BP_{P\text{-}END} = V_{T\text{-}END} - V_{ND}\,(\mathrm{HYDECA}) \qquad \text{(S1 Equation)}$$

where V_{ND} (HYDECA) is the V_{ND} estimated using HYDECA, and $V_{T\text{-}END}$ is the tracer total distribution volume (V_T) obtained in each region using numerical deconvolution (singular value decomposition) and calculated as [4, 5]:

$$V_{T-END} = K_i \int_0^{T_{END}} R_i(\tau)d\tau \qquad \text{(S2 Equation)}$$

with T_{END} the scan end time; and

$$BP_{P\text{-}NP2} = V_{T\text{-}NP2} - V_{ND}\,(\mathrm{HYDECA}) \qquad \text{(S3 Equation)}$$

where $V_{T\text{-}NP2}$ is the V_T obtained in each region using singular value decomposition and calculated as [4]:

$$V_{T-NP2} = \frac{K_i}{N}\sum_{n=N_1}^{n=N_Q} \frac{R_i^{-1}(n)}{log(1/n)} \qquad \text{(S4 Equation)}$$

where $R_i^{-1}(n)$ indicates the nth quantile of the residence density, which is the time at which $R_i(t)$ reaches a nth of its initial value, with $n = N_1, ..., N_Q$; Q is the number of considered quantiles; and the factor log(1/n) ensures that, if the residence density is exponential as in the case of a one-tissue compartment model, $R_i^{-1}(n)/\log(1/n)$ is equal to the mean of the residence distribution.

For each of the test-retest pair and region, we calculated the percent difference PD_{BPP} as $PD_{BPP} = 100\frac{|BP_{P-T}-BP_{P-RT}|}{(BP_{P-T}+BP_{P-RT})/2}$, where $BP_{P\text{-}T}$ indicates the test estimate, and $BP_{P\text{-}RT}$ the re-test estimate. We computed average and standard deviation (SD) (across subjects within the same tracer) of the PD_{BPP} values in each region, and compared them to those obtained for: $BP_{P\text{-}RR,LEGA} = V_T\,(\mathrm{LEGA}) - V_{T\text{-}RR,LEGA}$, which is based on the distribution volume in the purported reference region ($V_{T\text{-}RR,LEGA}$), estimated using Likelihood Estimation in Graphical Analysis (LEGA) [6]; $BP_{P\text{-}RR,2TCM} = V_T\,(\mathrm{2TCM}) - V_{T\text{-}RR,2TCM}$, with V_T (2TCM) and $V_{T\text{-}RR,2TCM}$ the V_T obtained in each target region and reference region, respectively, using the two-tissue compartment model (2TCM); $BP_{P\text{-}\alpha} = V_T(\mathrm{LEGA}) - \alpha V_{T\text{-}RR,LEGA}$ and $BP_{P\text{-}d} = V_T(\mathrm{LEGA}) - (V_{T\text{-}RR,LEGA} - d)$, with α and d derived from the blocking data as described in the manuscript.

Similarly, we also calculated alternative nonparametric binding potentials:

$$BP_{ND\text{-}END} = BP_{P\text{-}END}/V_{ND}\,(\mathrm{HYDECA}) \qquad \text{(S5 Equation)}$$

and

$$BP_{ND\text{-}NP2} = BP_{P\text{-}NP2}/V_{ND} \text{ (HYDECA)} \qquad \text{(S6 Equation)}$$

and compared their test-retest percent difference (PD_{BPND}; average and SD values computed in each region as with BP_P above) to that of: $BP_{ND\text{-}RR,LEGA} = BP_{P\text{-}RR,LEGA}/V_{T\text{-}RR,LEGA}$; $BP_{ND\text{-}RR,2TCM} = BP_{P\text{-}RR,2TCM}/V_{T\text{-}RR,2TCM}$; $BP_{ND\text{-}\alpha} = BP_{P\text{-}\alpha}/\alpha V_{T\text{-}RR,LEGA}$; and $BP_{ND\text{-}d} = BP_{P\text{-}d}/(V_{T\text{-}RR,LEGA} - d)$.

S1 and **S2 Figs** summarize the reproducibility of alternative nonparametric binding potentials that can be derived using HYDECA, with β and γ optimized using either strategies, and the reproducibility obtained for all the other binding potentials. PD_{BPP} values obtained using HYDECA with either sets of optimized tuning parameters are close to each other (**S1 Fig**). Overall, average PD_{BPP} values obtained for binding potentials based on HYDECA are better or comparable to values obtained using $V_{T\text{-}RR,LEGA}$. Average percent difference values for $BP_{P\text{-}RR,2TCM}$ are consistently the worst in the case of [^{11}C]DASB, and overall in the case of [^{11}C]CUMI-101. Average percent difference values based on the scaled $\alpha V_{T\text{-}RR,LEGA}$ or corrected $V_{T\text{-}RR,LEGA} - d$ are better than corresponding values based on $V_{T\text{-}RR,LEGA}$ alone, with both tracers, and are comparable or, in some regions, better than values obtained for estimates based on HYDECA. With [^{11}C]DASB, PD_{BPP} values obtained for estimates based on HYDECA are the best in amygdala (AMY), temporal lobe (TEM) (HYDECA using $\beta_{opt\text{-}S}$, $\gamma_{opt\text{-}S}$) and ventral striatum (VST) considering $BP_{P\text{-}END}$. With [^{11}C]CUMI-101, PD_{BPP} values obtained for estimates based on HYDECA are the best in hippocampus (HIP), TEM and cingulate (CIN) considering $BP_{P\text{-}END}$; and HIP (for HYDECA using $\beta_{opt\text{-}S}$, $\gamma_{opt\text{-}S}$) considering $BP_{P\text{-}NP2}$.

For both tracers PD_{BPND} values for BP_{ND} estimates obtained using $V_{T\text{-}RR,LEGA}$ are in general, on average, better than PD_{BPND} values obtained based on HYDECA, with the exception of $BP_{ND\text{-}END}$ in AMY and TEM with [^{11}C]DASB; and $BP_{ND\text{-}END}$ in HIP (for HYDECA using $\beta_{opt\text{-}B}$ and $\gamma_{opt\text{-}B}$), and $BP_{ND\text{-}NP2}$ in TEM and occipital lobe (OCC), with [^{11}C]CUMI-101 (**S2 Fig**). Average percent difference values for $BP_{ND\text{-}RR,2TCM}$ are the worst in the case of [^{11}C]DASB, with few exceptions, but overall comparable to $BP_{ND\text{-}RR,LEGA}$ in the case of [^{11}C]CUMI-101. Average percent difference values based on the scaled $\alpha V_{T\text{-}RR,LEGA}$ or corrected $V_{T\text{-}RR,LEGA} - d$ are overall the best with both tracers. The SD values for the PD_{BPND} are comparable across methods, with exclusion of $BP_{ND\text{-}RR,2TCM}$ in the case of [^{11}C]DASB.

1. Ogden RT, Ojha A, Erlandsson K, Oquendo MA, Mann JJ, Parsey RV. In vivo quantification of serotonin transporters using [(11)C]DASB and positron emission tomography in humans: modeling considerations. Journal of cerebral blood flow and metabolism : official journal of the International Society of Cerebral Blood Flow and Metabolism. 2007;27(1):205-17. doi: 10.1038/sj.jcbfm.9600329. PubMed PMID: 16736050; PubMed Central PMCID: PMC3784003.
2. Milak MS, DeLorenzo C, Zanderigo F, Prabhakaran J, Kumar JS, Majo VJ, et al. In vivo quantification of human serotonin 1A receptor using 11C-CUMI-101, an agonist PET radiotracer. Journal of nuclear medicine : official publication, Society of Nuclear Medicine. 2010;51(12):1892-900. Epub 2010/11/26. doi: 10.2967/jnumed.110.076257. PubMed PMID: 21098796; PubMed Central PMCID: PMCPmc3856257.
3. Innis RB, Cunningham VJ, Delforge J, Fujita M, Gjedde A, Gunn RN, et al. Consensus nomenclature for in vivo imaging of reversibly binding radioligands. Journal of cerebral blood flow and metabolism : official journal of the International Society of Cerebral Blood Flow and Metabolism. 2007;27(9):1533-9. Epub 2007/05/24. doi: 10.1038/sj.jcbfm.9600493. PubMed PMID: 17519979.
4. Zanderigo F, Parsey RV, Todd Ogden R. Model-free quantification of dynamic PET data using nonparametric deconvolution. Journal of cerebral blood flow and metabolism : official journal of the International Society of Cerebral Blood Flow and Metabolism. 2015;35(8):1368-

79. doi: 10.1038/jcbfm.2015.65. PubMed PMID: 25873427; PubMed Central PMCID: PMC4528013.
5. Jiang CR, Aston JA, Wang JL. A Functional Approach to Deconvolve Dynamic Neuroimaging Data. J Am Stat Assoc. 2016;111(513):1-13. doi: 10.1080/01621459.2015.1060241. PubMed PMID: 27226673; PubMed Central PMCID: PMCPMC4867865.
6. Ogden RT. Estimation of kinetic parameters in graphical analysis of PET imaging data. Statistics in medicine. 2003;22(22):3557-68. Epub 2003/11/06. doi: 10.1002/sim.1562. PubMed PMID: 14601019.
