# Supplementary material for: A hybrid deconvolution approach for estimation of in vivo non-displaceable binding for brain PET targets without a reference region
Source: PLoS One. 2017 May 1;12(5):e0176636. doi: 10.1371/journal.pone.0176636 (PMC5411064; doi:10.1371/journal.pone.0176636)
Supplement: S2 Text — Supporting information and equations accompanying S3 and S4 Figs. (PDF) [file pone.0176636.s011.pdf]

Comparison of binding potentials and test-retest percent difference values

$BP_{P\text{-}HYBRID}$

$$BP_{P\text{-}HYBRID} = V_T\,(LEGA) - V_{ND}\,(HYDECA) \quad \text{(S7 Equation)}$$

where V_T (LEGA) is the tracer total distribution volume (V_T) obtained in each region using Likelihood Estimation in Graphical Analysis (LEGA) [1], and V_{ND} (HYDECA) is the tracer non-displaceable binding (V_{ND}) estimated by HYDECA. The estimate of V_{ND} obtained by HYDECA in a subject is always lower than the corresponding tracer V_T in the non-ideal reference region ($V_{T\text{-}RR,LEGA}$). Therefore, as shown in **S3** and **S4 Figs**, $BP_{P\text{-}HYBRID}$ values based on HYDECA V_{ND} are by definition always higher than $BP_{P\text{-}RR,LEGA} = V_T$ (LEGA) – $V_{T\text{-}RR,LEGA}$ values based on $V_{T\text{-}RR,LEGA}$.

As shown in the scatter plot on the top right of **S3 Fig** (which reports the distance between test-retest percent difference values obtained using $BP_{P\text{-}HYBRID}$ and values obtained using $BP_{P\text{-}RR,LEGA}$, versus the corresponding distance between $BP_{P\text{-}HYBRID}$ and $BP_{P\text{-}RR,LEGA}$), for [^{11}C]DASB only in 57% (HYDECA with $\beta_{opt\text{-}S}$ and $\gamma_{opt\text{-}S}$) and 58% (HYDECA with $\beta_{opt\text{-}B}$ and $\gamma_{opt\text{-}B}$) of the cases the corresponding percent difference values based on HYDECA are lower (better reproducibility) than values based on $V_{T\text{-}RR,LEGA}$. As reported in **Figure 6** in the manuscript, in the dorsal caudate (DCA) only PD_{BPP} values based on HYDECA with $\beta_{opt\text{-}B}$, $\gamma_{opt\text{-}B}$ are on average lower than PD_{BPP} values based on $V_{T\text{-}RR,LEGA}$, even though $BP_{P\text{-}HYBRID}$ values are always higher.

For [^{11}C]CUMI-101 (**S4 Fig**), only in 79% (HYDECA with $\beta_{opt\text{-}S}$, $\gamma_{opt\text{-}S}$) and 63% (HYDECA with $\beta_{opt\text{-}B}$, $\gamma_{opt\text{-}B}$) of the cases the corresponding PD_{BPP} values based on HYDECA are lower (better reproducibility) than the PD_{BPP} values based on $V_{T\text{-}RR,LEGA}$.

$BP_{P\text{-}END}$

Differently from $BP_{P\text{-}HYBRID}$, $BP_{P\text{-}END}$ (please see definition in **S1 Text** or original publications [2, 3]) estimates based on HYDECA are not systematically higher than corresponding $BP_{P\text{-}RR,LEGA}$ values based on $V_{T\text{-}RR,LEGA}$, as shown in the middle panels of **S3** and **S4 Figs**. For [^{11}C]DASB (**S3 Fig**), only in the 64% (HYDECA with $\beta_{opt\text{-}S}$, $\gamma_{opt\text{-}S}$) and 66% (HYDECA with $\beta_{opt\text{-}B}$, $\gamma_{opt\text{-}B}$) of the cases $BP_{P\text{-}END}$ values are higher than $BP_{P\text{-}RR.LEGA}$ values, and only in the 47% (HYDECA with $\beta_{opt\text{-}S}$, $\gamma_{opt\text{-}S}$) and 52% (HYDECA with $\beta_{opt\text{-}B}$, $\gamma_{opt\text{-}B}$) of these cases the corresponding PD_{BPP} values based on HYDECA are lower (better reproducibility) than values based on $V_{T\text{-}RR,LEGA}$.

For [^{11}C]CUMI-101 (**S4 Fig**), only in the 79% (HYDECA with $\beta_{opt\text{-}S}$, $\gamma_{opt\text{-}S}$) and 67% (HYDECA with $\beta_{opt\text{-}B}$, $\gamma_{opt\text{-}B}$) of the cases $BP_{P\text{-}END}$ values are higher than values based on $V_{T\text{-}RR,LEGA}$, and only in the 79% (HYDECA with $\beta_{opt\text{-}S}$, $\gamma_{opt\text{-}S}$) and 81% (HYDECA with $\beta_{opt\text{-}B}$, $\gamma_{opt\text{-}B}$) of these cases the corresponding PD_{BPP} values based on HYDECA are lower than values based on $V_{T\text{-}RR,LEGA}$.

$BP_{P\text{-}NP2}$

As with $BP_{P\text{-}END}$, $BP_{P\text{-}NP2}$ estimates (please see definition in **S1 Text** or original publications [2, 3]) based on HYDECA are not always higher than corresponding $BP_{P\text{-}RR,LEGA}$ values based on $V_{T\text{-}RR,LEGA}$, as shown in the bottom panels of **S3** and **S4 Figs**.

For [^{11}C]DASB (**S3 Fig**), in the 94% (HYDECA with $\beta_{opt\text{-}S}$, $\gamma_{opt\text{-}S}$) and 98% (HYDECA with $\beta_{opt\text{-}B}$, $\gamma_{opt\text{-}B}$) of the cases $BP_{P\text{-}NP2}$ values are higher than $BP_{P\text{-}RR,LEGA}$ values; however, only in 43% (HYDECA with $\beta_{opt\text{-}S}$, $\gamma_{opt\text{-}S}$) and 52% (HYDECA with $\beta_{opt\text{-}B}$, $\gamma_{opt\text{-}B}$) of these cases, the corresponding PD_{BPP} values based on HYDECA are lower (better reproducibility) than values based on $V_{T\text{-}RR,LEGA}$.

For [^{11}C]CUMI-101 (**S4 Fig**), in all cases $BP_{P\text{-}NP2}$ values are higher than $BP_{P\text{-}RR,LEGA}$ values; however, only in 58% (HYDECA with $\beta_{opt\text{-}S}$, $\gamma_{opt\text{-}S}$) and 50% (HYDECA with $\beta_{opt\text{-}B}$, $\gamma_{opt\text{-}B}$)

of these cases, the corresponding PD_{BPP} values based on HYDECA are lower than values based on $V_{T\text{-}RR,LEGA}$.

All together these observations suggest that the fact that BP_P estimates can be lower when based on $V_{T\text{-}RR,LEGA}$ does not necessarily lead to higher PD_{BPP} values, and that the worse test-retest reproducibility of the estimates based on $V_{T\text{-}RR,LEGA}$ is not due only to the magnitude of estimated V_{ND}.

1. Ogden RT. Estimation of kinetic parameters in graphical analysis of PET imaging data. Statistics in medicine. 2003;22(22):3557-68. Epub 2003/11/06. doi: 10.1002/sim.1562. PubMed PMID: 14601019.
2. Zanderigo F, Parsey RV, Todd Ogden R. Model-free quantification of dynamic PET data using nonparametric deconvolution. Journal of cerebral blood flow and metabolism : official journal of the International Society of Cerebral Blood Flow and Metabolism. 2015;35(8):1368-79. doi: 10.1038/jcbfm.2015.65. PubMed PMID: 25873427; PubMed Central PMCID: PMC4528013.
3. Jiang CR, Aston JA, Wang JL. A Functional Approach to Deconvolve Dynamic Neuroimaging Data. J Am Stat Assoc. 2016;111(513):1-13. doi: 10.1080/01621459.2015.1060241. PubMed PMID: 27226673; PubMed Central PMCID: PMCPMC4867865.
