# Supplementary material for: A hybrid deconvolution approach for estimation of in vivo non-displaceable binding for brain PET targets without a reference region
Source: PLoS One. 2017 May 1;12(5):e0176636. doi: 10.1371/journal.pone.0176636 (PMC5411064; doi:10.1371/journal.pone.0176636)
Supplement: S3 Text — Supporting information and equations accompanying S5–S7 Figs. (PDF) [file pone.0176636.s012.pdf]

Sensitivity to vascular correction

HYDECA, as well as all other quantification approaches used in PET, needs to correct the measured tissue time activity curves for the contribution from the tracer present in the vasculature. This applies to compartment models as well, unless the fractional blood volume value (usually indicated as V_B) is left as a free parameter to be estimated from the data along with the other parameters of the chosen compartment model. Our approach is to correct the tissue time activity curves with a pre-defined, brain-wide V_B of 5% before applying HYDECA, an approach to vascular correction commonly adopted in the Positron Emission Tomography (PET) field. If the choice of V_B is in error, then naturally the performance of any quantification procedure may be affected. An optimized V_B value should be pre-assessed before using HYDECA (or any PET quantification approach with a fixed V_B), if pathological changes in the V_B value are suspected in the population at hand.

To investigate the sensitivity of HYDECA estimates of the non-displaceable distribution volume (V_{ND}) to a potentially erroneous vascular correction of the tissue time activity curves, we ran an additional simulation study as follows. For both radiotracers, we considered for each of the subjects in the two available test-retest datasets [1, 2] (baseline scans) the time activity curves corrected with V_B = 5% in all regions that are considered simultaneously by HYDECA (these curves are denoted here as $C_{TISSUE}(t)$). Then, for each of 20 V_B values (ranging from 1% to 20% with a step of 1%), we generated for each subject 20 new sets of tissue time activity curves using the following equation: $C(t) = (1-V_B)C_{TISSUE}(t) + V_BC_B(t)$, with $C_B(t)$ the subject radiotracer total radioactivity in blood. We then corrected every new set of tissue time activity curves with a fixed V_B of 5% so that, for each subject, 19 of the new sets of tissue time activity curves were erroneously corrected for vasculature presence (with an error in V_B ranging from -4% to +15%), (and thus only one set was accurately corrected). In each subject, we then estimated V_{ND} using HYDECA in each of the 20 new sets of tissue time activity curves, and compared each of the V_{ND} estimates corresponding to erroneous vascular correction to the one corresponding to no error in vascular correction. As a measure of comparison, we used the percent difference defined as follows: $PD_{errVC} = 100[(V_{ND\text{-}errVC} - V_{ND\text{-}NOerrVC})/V_{ND\text{-}NOerrVC}]$, with $V_{ND\text{-}errVC}$ the V_{ND} value estimated at each instance with erroneously corrected time activity curves, and $V_{ND\text{-}NOerrVC}$ the V_{ND} value estimated in correspondence of the accurately corrected set of time activity curves.

S5 Fig shows, for both tracers, PD_{errVC} values (average and standard deviation, SD, across subjects within each tracer; y-axis), as a function of the difference between true V_B value (which varied from 1% to 20%, as described above) and the value adopted for correction (which is fixed at 5%) (x-axis). HYDECA estimates of V_{ND} appear to be robust to erroneous vascular correction (PD_{errVC} between -10% and +10%) for errors in V_B in the range -4% to +5% for [^{11}C]DASB, and -4% to +7% for [^{11}C]CUMI-101.

S6 and **S7 Figs** show the residue function R(t) curves deconvolved in all considered regions in correspondence of different errors in V_B, and in correspondence of no error, and the corresponding HYDECA cost functions, in 2 representative subjects, one for each tracer.

1. Ogden RT, Ojha A, Erlandsson K, Oquendo MA, Mann JJ, Parsey RV. In vivo quantification of serotonin transporters using [(11)C]DASB and positron emission tomography in humans: modeling considerations. Journal of cerebral blood flow and metabolism : official journal of the International Society of Cerebral Blood Flow and Metabolism. 2007;27(1):205-17. doi: 10.1038/sj.jcbfm.9600329. PubMed PMID: 16736050; PubMed Central PMCID: PMC3784003.
2. Milak MS, DeLorenzo C, Zanderigo F, Prabhakaran J, Kumar JS, Majo VJ, et al. In vivo quantification of human serotonin 1A receptor using 11C-CUMI-101, an agonist PET radiotracer. Journal of nuclear medicine : official publication, Society of Nuclear Medicine.

2010;51(12):1892-900. Epub 2010/11/26. doi: 10.2967/jnumed.110.076257. PubMed PMID: 21098796; PubMed Central PMCID: PMCPmc3856257.
