# Supplementary material for: A hybrid deconvolution approach for estimation of in vivo non-displaceable binding for brain PET targets without a reference region
Source: PLoS One. 2017 May 1;12(5):e0176636. doi: 10.1371/journal.pone.0176636 (PMC5411064; doi:10.1371/journal.pone.0176636)
Supplement: S4 Text — Supporting information and equations accompanying S8 and S9 Figs. (PDF) [file pone.0176636.s013.pdf]

Assumption of a mono-exponential non-displaceable residue function

To ensure identifiability of the two components of the residue function curve R(t) (non-displaceable, $R_{ND}(t)$, and specific, $R_S(t)$), HYDECA needs to assume a certain shape to describe the non-displaceable component. We chose, in part for its simplicity, a mono-exponential function, which we regard as a reasonable choice, since it would represent the impulse response function in the case of an "ideal" reference region with total distribution volume equal to the non-displaceable distribution volume (V_{ND}). Assuming a mono-exponential function for $R_{ND}(t)$ represents an approximation (if a two-tissue compartment model, 2TCM, is needed to describe the data in a given region, $R_{ND}(t)$ would be described by two exponentials). We note that a similar approximation is central in the development of the very widely used simplified reference tissue model (SRTM) [1]. Specifically, SRTM assumes that the total (non-displaceable plus specific) impulse response function of the target region (which, as well, would be a two-exponential function) can be reasonably approximated by a mono-exponential curve.

Here we report, for the interested reader, the derivation of the expression of $R_{ND}(t)$ in the case of a 2TCM. The concentration of tracer in the two tissue compartments can be described by the following system of equations

$$\begin{cases} \frac{dC_{ND}(t)}{dt} = K_1 C_P(t) - (k_2 + k_3) C_{ND}(t) + k_4 C_S(t) \\ \frac{dC_S(t)}{dt} = k_3 C_{ND}(t) - k_4 C_S(t) \end{cases} \quad \text{(S8 Equation)}$$

where $C_{ND}(t)$, $C_P(t)$, and $C_S(t)$ are the tracer concentration in the non-displaceable, plasma, and specific compartment, respectively; using Laplace transformation we obtain

$$\begin{cases} sC_{ND}(s) = K_1 C_P(s) - (k_2 + k_3) C_{ND}(s) + k_4 C_S(s) \\ sC_S(s) = k_3 C_{ND}(s) - k_4 C_S(s) \end{cases} \quad \text{(S9 Equation)}$$

where $C_{ND}(s)$, $C_P(s)$, and $C_S(s)$ are the Laplace transforms of $C_{ND}(t)$, $C_P(t)$, and $C_S(t)$, respectively; with simple substitutions the system of equations can be expressed as

$$\begin{cases} C_{ND}(s) = \frac{K_1(s+k_4)C_P(s)}{s^2+s(k_2+k_3+k_4)+k_2k_4} \\ C_S(s) = \frac{k_3 C_{ND}(s)}{s+k_4} \end{cases} \quad \text{(S10 Equation)}$$

from which it follows that

$$IRF_{ND}(s) = \frac{C_{ND}(s)}{K_1 C_P(s)} = \frac{s+k_4}{s^2+s(k_2+k_3+k_4)+k_2k_4} \quad \text{(S11 Equation)}$$

where $IRF_{ND}(s)$ is the Laplace transforms of $R_{ND}(t)$; with simple calculations $IRF_{ND}(s)$ can be expressed as

$$IRF_{ND}(s) = \frac{C_{ND}(s)}{K_1 C_P(s)} = \frac{s+k_4}{s^2+s(k_2+k_3+k_4)+k_2k_4} = \frac{A}{s+\alpha_1} + \frac{B}{s+\alpha_2} \quad \text{(S12 Equation)}$$

with

$$\begin{cases} \alpha_1 = \frac{k_2+k_3+k_4-\sqrt{(k_2+k_3+k_4)^2-4k_2k_4}}{2} \\ \alpha_2 = \frac{k_2+k_3+k_4+\sqrt{(k_2+k_3+k_4)^2-4k_2k_4}}{2} \end{cases} \quad \text{(S13 Equation)}$$

and

$$\begin{cases} A = \frac{k_4-\alpha_1}{\alpha_2-\alpha_1} \\ B = \frac{\alpha_2-k_4}{\alpha_2-\alpha_1} \end{cases} \quad \text{(S14 Equation)}$$

which leads to the following expression of $R_{ND}(t)$ in time domain

$$IRF_{ND}(t) = R_{ND}(t) = Ae^{-\alpha_1 t} + Be^{-\alpha_2 t} \quad \text{(S15 Equation)}$$

To evaluate the validity of such approximation for the two tracers at hand, we considered the K_1 and k_2 values we used for the simulation in 8 different cases (in each tracer, 2 regions, the one with the lowest and the highest tracer total distribution volume, V_T, respectively, with 2 different settings of V_{ND}, $V_{ND} = 3$ and $V_{ND} = 5$), and evaluated how much a two-exponential $R_{ND}(t)$ curve from **S15 Equation** deviated from the assumption of a mono-exponential $R_{ND}(t)$ curve (which is exactly correct when $k_3 = k_4 = 0$) as k_3 and k_4 vary in a range (0 to 3.5) that covers the kinetics of the two considered tracers. We evaluated the difference between the two curves at each combination of k_3 and k_4 values by calculating the average (across time points) square distance between the two curves. As **S8** and **S9 Figs** show in the case of $[^{11}C]$DASB and $[^{11}C]$CUMI-101, respectively, given a certain value of k_4, the approximation of a mono-exponential is less and less appropriate as the value of k_3 increases, while given a certain value of k_3, the approximation is more and more appropriate as the value of k_4 increases. Thus indicates that a mono-exponential approximation for $R_{ND}(t)$ would be problematic only in the situation in which $k_3 >> k_4$, which means that more tracer molecules transit in a given amount of time from the non-displaceable binding state into the specific binding state than vice versa.

We remind the reader: 1) that HYDECA uses data across many regions, for some of which the mono-exponential assumption may hold better than for others, and provides a brain-wide value of V_{ND} that satisfies certain constraints (via the HYDECA cost function) on average across such regions; and 2) that parts of the $R_{ND}(t)$ curve that are potentially erroneously determined in a region due to the simplifying mono-exponential assumption for $R_{ND}(t)$ are likely to be captured by the corresponding nonparametric $R_S(t)$, for which there is no assumption besides being positive and monotonic.

1. Lammertsma AA, Hume SP. Simplified reference tissue model for PET receptor studies. NeuroImage. 1996;4(3 Pt 1):153-8. Epub 1996/12/01. doi: 10.1006/nimg.1996.0066. PubMed PMID: 9345505.
